# A novel human iPSC model of COL4A1/A2 small vessel disease unveils a key pathogenic role of matrix metalloproteinases

**DOI:** 10.1016/j.stemcr.2023.10.014

**Published:** 2023-11-16

**Authors:** Maha Al-Thani, Mary Goodwin-Trotman, Steven Bell, Krushangi Patel, Lauren K. Fleming, Catheline Vilain, Marc Abramowicz, Stuart M. Allan, Tao Wang, M. Zameel Cader, Karen Horsburgh, Tom Van Agtmael, Sanjay Sinha, Hugh S. Markus, Alessandra Granata

**Affiliations:** 1 Department of Clinical Neurosciences, Victor Phillip Dahdaleh Heart and Lung Research Institute, University of Cambridge and Royal Papworth Hospital, Cambridge, UK; 2 School of Cardiovascular and Metabolic Health, University of Glasgow, Glasgow, UK; 3 Department of Genetics, Hôpital Erasme, ULB Center of Human Genetics, Universite Libre de Bruxelles, Bruxelles, Belgium; 4 Division of Neuroscience, School of Biological Sciences, Faculty of Biology, Medicine and Health, The University of Manchester, Manchester, UK; 5 Geoffrey Jefferson Brain Research Centre, Manchester Academic Health Science Centre, Northern Care Alliance NHS Foundation Trust, The University of Manchester, Manchester, UK; 6 Division of Evolution, Infection and Genomics, School of Biological Sciences, Faculty of Biology, Medicine and Health, The University of Manchester, Manchester, UK; 7 Nuffield Department of Clinical Neurosciences, Kavli Institute of Nanoscience Discovery, Dorothy Crowfoot Hodgkin Building, Sherrington Road, University of Oxford, Oxford, UK; 8 Centre for Discovery Brain Sciences, University of Edinburgh, Edinburgh, UK; 9 Wellcome-MRC Cambridge Stem Cell Institute, Jeffrey Cheah Biomedical Centre, University of Cambridge, Cambridge, UK; 10 Department of Neurology, Cambridge University Hospitals NHS Foundation Trust, Cambridge, UK

**Keywords:** Cerebral small vessel disease, COL4A1/A2, blood-brain barrier, human induced pluripotent stem cells, disease modelling, mural cells, endothelial cells, extracellular matrix, matrix metalloproteinases

## Abstract

Cerebral small vessel disease (SVD) affects the small vessels in the brain and is a leading cause of stroke and dementia. Emerging evidence supports a role of the extracellular matrix (ECM), at the interface between blood and brain, in the progression of SVD pathology, but this remains poorly characterized. To address ECM role in SVD, we developed a co-culture model of mural and endothelial cells using human induced pluripotent stem cells from patients with *COL4A1*/*A2* SVD-related mutations. This model revealed that these mutations induce apoptosis, migration defects, ECM remodeling, and transcriptome changes in mural cells. Importantly, these mural cell defects exert a detrimental effect on endothelial cell tight junctions through paracrine actions. *COL4A1*/*A2* models also express high levels of matrix metalloproteinases (MMPs), and inhibiting MMP activity partially rescues the ECM abnormalities and mural cell phenotypic changes. These data provide a basis for targeting MMP as a therapeutic opportunity in SVD.

## Highlights

• A novel human iPSC-derived model of genetic SVD due to collagen IV mutations
• SVD mural cells show ECM abnormalities and contribute to endothelial defects
• ECM and endothelial cells abnormalities can be rescued by MMP inhibition

## Graphical abstract

**Figure:**
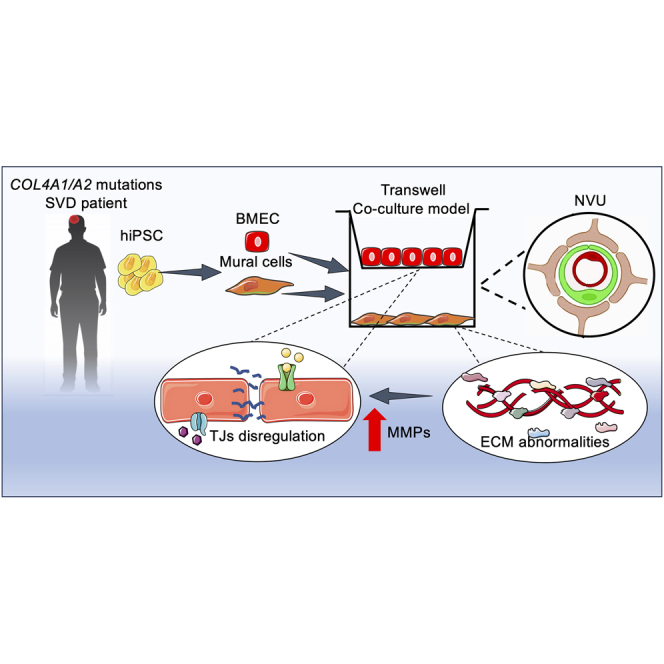

## Teaser

This paper describes the development of a novel human iPSC model for collagen IV mutations, which are a hereditary form of small vessel disease causing stroke and dementia. The authors use this model to explore blood-brain-barrier abnormalities by Transwell co-culture system and extracellular matrix changes in the mural cells, contributing to barrier dysfunction, and identify MMPs as potential therapeutic approach.

## Introduction

Cerebral small vessel disease (SVD) is a leading cause of age-related cognitive decline and contributes to up to 45% of dementia cases worldwide (Gorelick et al., 2011). SVD is also responsible for 20% of ischemic strokes and is a common pathology underlying intracerebral hemorrhage (ICH) (Wardlaw et al., 2019). SVD refers to the sum of all pathological processes that affect the small vessels of the brain, and with an aging population, SVD has major and growing global socio-economic impact (Lam et al., 2022). However, despite its importance, therapeutic approaches for SVD remain limited because of the lack of mechanistic understanding and relevant models required for target identification and drug discovery (Smith and Markus, 2020).

SVD features are associated with advancing age and several vascular risk factors (Wardlaw et al., 2014). Genetic factors have also been reported to be important, with the identification of monogenic forms of SVD (Mancuso et al., 2020) and common variants that increase the risk for sporadic SVD (Chung et al., 2021; Rannikmäe et al., 2015; Traylor et al., 2021). Dominant mutations in collagen type IV, a major component of the microvascular extracellular matrix (ECM), cause SVD presenting with both ICH and ischemia (Gould et al., 2005; Jeanne et al., 2012). *COL4A1* and *COL4A2* mutations cause highly penetrant multi-system disorders by disrupting the ECM homeostasis and leading to ICH and porencephaly in human and mouse models (van Agtmael et al., 2005; Joutel and Faraci, 2014; Murray et al., 2014). Most mutations occur in a glycine (G) residue of the G-X-Y repeat, which characterizes the collagenous domain, and the position of the mutation appeared to correlate with SVD severity (Jeanne et al., 2015). Conversely, variant within the 3′ UTR of *COL4A1* located in a putative miR-29 microRNA binding site results in *COL4A1* upregulation and causes a severe form of ischemic SVD, distinct from the *COL4A1* missense glycine mutation phenotype (Siitonen et al., 2017; Verdura et al., 2016). Patient fibroblasts with *COL4A1* and *COL4A2* gene duplications have also shown increased gene expression, supporting evidence for the pathogenicity of *COL4A1A*/*2* overexpression in SVD (Kuuluvainen et al., 2021). Importantly, both monogenic and sporadic forms of *COL4A*-related SVD are likely to share similar pathological mechanisms, as rare coding variants in *COL4A1*/*A2* also occur in sporadic form of ICH, while common *COL4A1*/*A2* non-coding variants have been identified as risk factor for sporadic lacunar stroke (Chung et al., 2019; Persyn et al., 2020; Traylor et al., 2021), sporadic ICH (Malik et al., 2018; Rannikmäe et al., 2015), and white matter hyperintensities (Persyn et al., 2020) in the general population. This suggests that insights gained from a model of monogenic *COL4A1*/*A2* are likely to be relevant to common SVD.

Although the mechanisms leading to SVD are ill defined, there is an emerging focus on the role of the ECM. The ECM of cerebral blood vessels is a key component at the interface between the cerebral microcirculation and the brain, providing structural support to the blood-brain barrier (BBB) as well as influencing cell behavior (Joutel et al., 2016). Genetic studies have revealed that most monogenic forms of SVD are caused by mutations either in genes encoding ECM proteins or in proteins regulating ECM function (Joutel et al., 2016). In addition to this, our recent work has shown that genes related to SVD, including *COL4A1* and *COL4A2*, are significantly enriched in the cerebrovascular ECM network in both mouse and human brain (Pokhilko et al., 2021). To date, the mechanisms by which these ECM defects cause disease remain poorly understood. This underscores the clear need for new models relevant to human SVD.

To provide insights into the pathological mechanisms underlying COL4A1/A2-related SVD, we established a human induced pluripotent stem cell (hiPSC)-based “disease in a dish” model from two individuals with two representative glycine substitutions in the G-X-Y repeat, one in *COL4A1* (G755R) and the other in *COL4A2* (*G702D*) gene (Murray et al., 2014; Shah et al., 2010). We differentiated the hiPSCs into mural cells (MCs) and endothelial cells (ECs) and undertook phenotypic and functional assays and transcriptomic analysis.

## Results

### Establishment and characterization of COL4A1/A2 hiPSC-derived MCs and ECs

**Figure 1:**
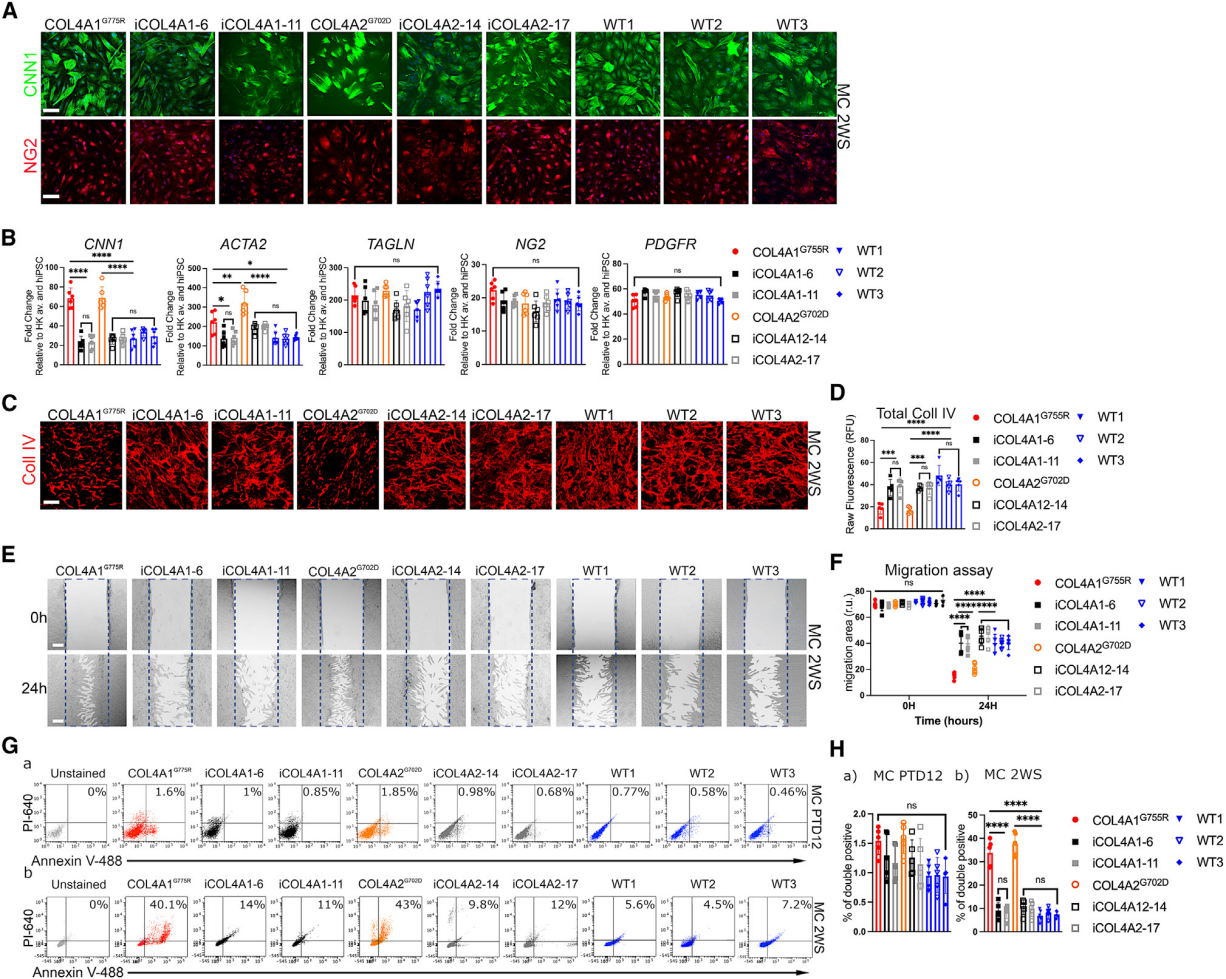
COL4A1^*G755R*^ and COL4A2^*G702D*^ hiPSC-derived mural cells (MCs) show abnormal collagen IV and phenotypic changes (A) Immunostaining for calponin (CNN1) and nerve/glial antigen 2 (NG2) in hiPSC-derived MCs cultured for 2 weeks in serum containing media (2WS) for COL4A1^*G755R*^, COL4A2^*G702D*^, 2 isogenic subclones for A1 (iCOL4A1-6 and iCOL4A1-11) and A2 (iCOL4A2-14 and iCOL4A2-17), and three healthy controls (WT1, WT2, and WT3; see also Table S1). (B) quantitative real-time PCR analysis for MC markers, including *CNN1*, *ACTA2*, *TAGLN*, *NG2*, and *PDGFR* (n = 6). (C) Immunostaining for collagen IV in the ECM of MCs show significant decreased levels in COL4A1^*G755R*^ and COL4A2^*G702D*^ when quantified as total fluorescence (D) compared with isogenic and WT controls (n = 6). (E and F) Representative images of scratch assays for hiPSC MCs (E) and (F) quantification of the areas showing increased migration rate for COL4A1/A2 mutant MC compared with controls (n = 6). (G) Flow cytometric analysis of annexin V-488 and propidium iodide (PI-640) in hiPSC MCs after 12 days of differentiation in PDGFBB + TGF-β1 (PTD12, early stage; a) and at late stage (2WS; b) show higher apoptotic rate in COL4A1/A2 mutant compared with control MC lines at 2WS (n = 5). (H) Nuclei were stained with DAPI; scale bar, 100 μm. Results are presented as mean ± SD of n independent experiments. ^∗^p < 0.05, ^∗∗^p < 0.01, ^∗∗∗^p < 0.001, and ^∗∗∗∗^p < 0.0001; ns (not significant). Statistical analysis was performed using 2-way ANOVA with Tukey’s multiple comparison test.

Two hiPSC lines with typical SVD-associated SNPs in *COL4A* genes were used in this study: a *COL4A1*^*G755R*^ with a G>A substitution in exon 30 of *COL4A1* gene resulting in a change from a glycine to arginine at position 755 from a symptomatic patient and a *COL4A2*^*G702D*^ with a G>A replacement in exon 28 of *COL4A2* gene, resulting in a change from a glycine to aspartic acid at position 702 from the asymptomatic father of a patient (Table S1) (Murray et al., 2014; Shah et al., 2010). To control for genetic background, we generated isogenic corrected lines, in which the mutant allele (A) in *COL4A1* and *COL4A2* hiPSCs were substituted with the wild-type (WT) allele (G), and two subclones were used for each CRISPRed line (Tables S1 and S2; Figure S1A). As further controls, we used three WT hiPSC lines from healthy individuals (Table S1). hiPSC lines were characterized for pluripotency marker expression by immunostaining, quantitative real-time PCR profiling, and formation of the 3-germ layers (Figures S1B–S1D). hiPSCs were successfully differentiated into MCs of neural crest origin as previously described (Cheung et al., 2012; Serrano et al., 2019) (Figure S2A) and characterized for specific marker expression for neural crest (Figures S2B and S2C) and for MC markers at day 12 of PDGFBB+TGF-β1 differentiation (PTD12) at mRNA levels (Figure S2D) and at the fully differentiated stage at 2 weeks in serum containing media (2WS) using immunohistochemistry and quantitative real-time PCR (Figures 1A, 1B, S2D, and S2E).

MCs express both specific markers for smooth muscle cells (*CNN1*, *ACTA2*, and *TAGLN*) and pericytes (*NG2* and *PDGFRA*), with the disease lines showing significantly increased expression levels for *CNN1* and *ACTA2* at a late stage of differentiation (2WS) (Figures 1B and S2D)*.* MCs are known to produce a variety of ECM proteins, including collagen IV. To assess collagen IV levels in the ECM, both *COL4A1*/*A2* disease and isogenic hiPSC-derived MCs were plated at equal density, decellularized, and stained with a specific antibody that recognized both collagen IV α1 and α2 chains (Figure 1C). There was a significant reduction in collagen IV staining in the ECM of the disease *COL4A1*/*A2* mutant lines compared with the controls, as seen in patient fibroblasts (Figure 1D) (Murray et al., 2014). Moreover, hiPSC-derived MCs with *COL4A1*^*G755R*^ and *COL4A2*^*G702D*^ have increased migration ability in a scratch assay compared with controls (Figures 1E and 1F). MCs at 2WS also exhibit higher apoptotic levels when stained for annexin V and propidium iodide (PI) using flow cytometry compared with the controls (Figures 1G and 1Hb), similar to previous findings from primary patient fibroblasts and skin biopsy (Murray et al., 2014). Interestingly, no significant changes in apoptotic rates were seen at an earlier stage (PTD12; Figures 1G and 1Ha), at which stage ECM deposition of collagen IV cannot be detected (Figures S2F and S2G). Thus, higher apoptotic rates might be a consequence of increased levels of abnormal collagen IV in the ECM. These data indicate that our hiPSC MCs recapitulate defects of *COL4A1*/*2* mutations and thus represent a valid model to explore disease mechanisms.

### MCs contribute to the barrier phenotype in co-culture and paracrine systems

**Figure 2:**
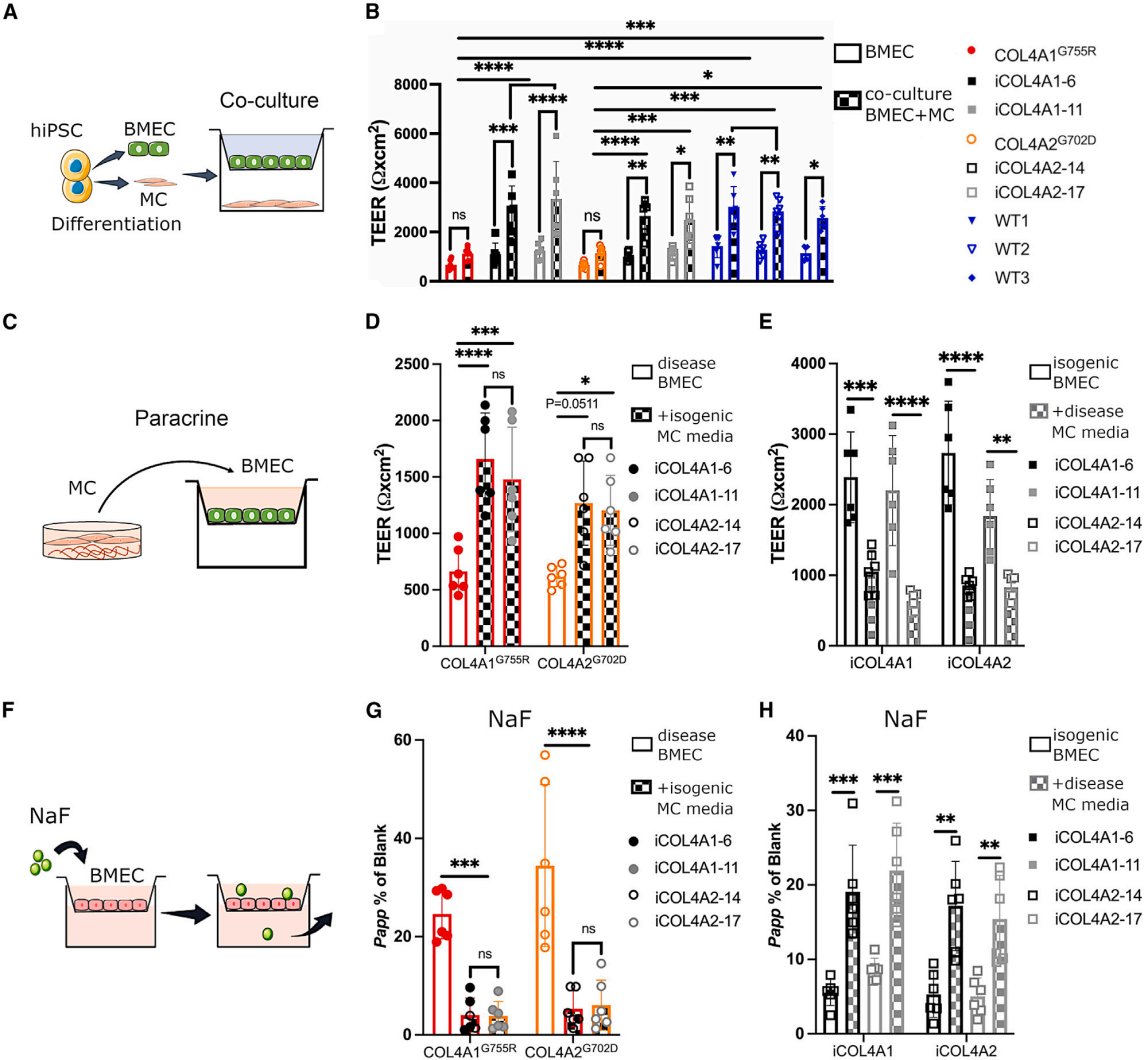
hiPSC-derived mural cells (MCs) contribute to barrier function in a Transwell co-culture system, and COL4A1/A2 lines exert a detrimental effect (A) Schematic of co-culture with hiPSC-derived MCs and brain microvascular endothelial-like cells (BMECs) in a Transwell device. (B) Transendothelial electrical resistance (TEER) peak values expressed as resistance (Ω) · cm^2^ for hiPSC-derived BMECs in co-culture with MCs increases compared with BMECs alone for isogenic iCOL4A1/A2 and WTs (n = 6). (C–E) Schematic of the MC paracrine experiment (C), with COL4A1^*G755R*^ and COL4A2^*G702D*^ BMEC TEER values benefiting from treatment with isogenic MC-conditioned media (D) (n = 6), while isogenic BMECs show decreased TEER values upon treatment with disease COL4A1/A2 MC-conditioned media (n = 6) (E). (F–H) Schematic of the sodium fluorescein (NaFl) permeability assay in Transwell setting (F). Isogenic MC paracrine effect positively reduces BMEC permeability in COL4A1^*G755R*^ and COL4A2^*G702D*^ lines after 6 days treatment (n = 6) (G), while disease MC-conditioned media-treated isogenic BMECs show increased permeability to NaFl compared with untreated BMECs (n = 6) (H). TEER, transendothelial electrical resistance; NaFl, sodium fluorescein; *Papp*, apparent permeability. Results are presented as mean ± SD of n independent experiments. ^∗^p < 0.05, ^∗∗^p < 0.01, ^∗∗∗^p < 0.001, and ^∗∗∗∗^p < 0.0001; ns (not significant). Statistical analysis was performed using 2-way ANOVA with Tukey’s multiple-comparison test.

Brain ECs are known for their barrier function in the BBB, which may be compromised in SVD pathology (Hussain et al., 2021). However, the impact of collagen IV mutations on the BBB and cross-talk between ECs and MCs remains poorly understood. To assess this, *COL4A1*/*A2* disease, isogenic, and WT lines were differentiated into brain microvascular endothelial-like cells (BMECs) using a previously established protocol (Figure S3A; (Hollmann et al., 2017)). These BMECs were characterized for expression of specific markers using flow cytometry and quantitative real-time PCR (Figures S3B–S3D). BMECs were then plated onto a two-dimensional (2D) Transwell setting alone or in presence of MC plated on the basolateral side and maintained for 6 days (Figure 2A). During this time, daily readings were taken of transendothelial electrical resistance (TEER), a robust indicator of EC barrier integrity. TEER measurements expressed as peak values relative to blank (Transwell with no cells) for BMECs alone and in co-culture with MCs were compared (Figure 2B). Isogenic and WT control MCs appear to promote barrier function by significantly increasing TEER values, while disease MCs have little effect on *COL4A1*/*A2* BMEC TEER (Figure 2B). Moreover, MCs were cultured with or without the addition of ascorbic acid in the media to promote collagen synthesis, and similar results were obtained.

Because in this setting, there is no cell-cell interaction between MCs and BMECs, we further assessed the differential MC paracrine effect on the barrier properties by treating the isogenic BMEC clones with conditioned media of *COL4A1*/*A2* MC and vice versa for 6 days (Figure 2C). Interestingly, TEER values of disease BMECs tend to benefit from the isogenic MC paracrine effect (Figure 2D). Conversely, disease MCs exert a paracrine effect by significantly decreasing TEER values in isogenic BMECs (Figure 2E). This MC-mediated paracrine effect on barrier phenotype was confirmed by sodium fluorescein (NaFl) size exclusion paracellular permeability assay (Figure 2F), with the isogenic MCs decreasing NaFl permeability, thus promoting barrier tightness (Figure 2G), while disease MCs appear to increase barrier permeability in isogenic BMECs (Figure 2H). Collectively, these data show the secretome of disease MCs to have detrimental effects on barrier function in *COL4A1*/*A2* SVD models.

### COL4A1/A2 MCs affect endothelial tight junction levels and distribution through a paracrine effect

**Figure 3:**
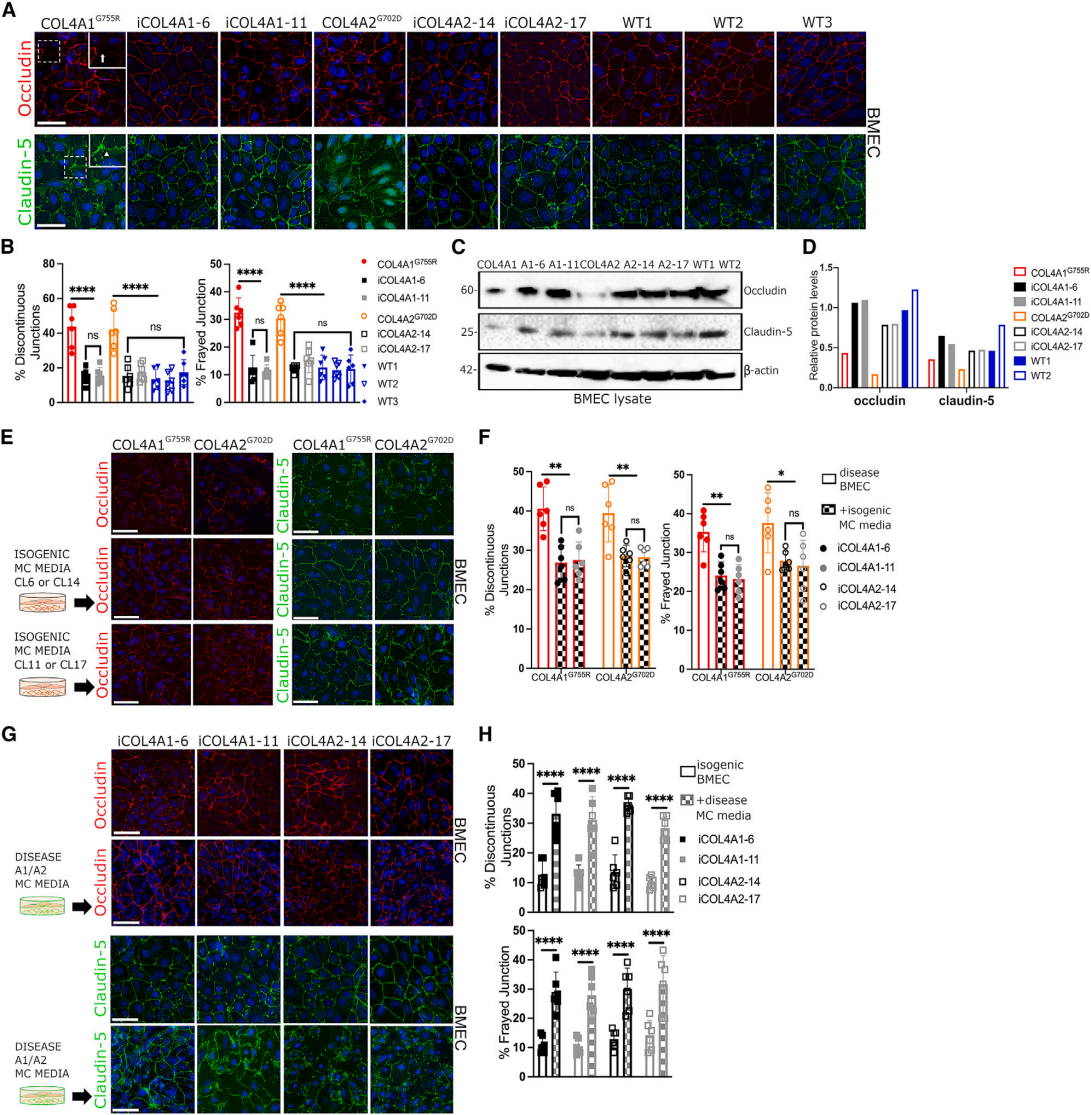
COL4A1/A2 mural cells contribute to endothelial tight junction abnormalities by paracrine effect (A) Junctional staining for occludin and claudin-5 in hiPSC-derived BMEC lines cultured alone showing discontinuous junction (white arrow) and frayed junction (white arrowed) in zoomed-in insert. (B) Quantification of discontinuous and frayed junctions show higher percentage in COL4A1^*G755R*^ and COL4A2^*G702D*^ lines compared with controls (n = 6). (C and D) Western blotting analysis and bands quantification show decreased total protein levels for occludin and claudin-5 in COL4A1^*G755R*^- and COL4A2^*G702D*^-derived BMECs compared with controls (A1 and A2 ISO) and WT1 and WT2. β-Actin was used as loading control (representative blot of n = 3). (E and F) Immunostaining analysis of COL4A1^*G755R*^ and COL4A2^*G702D*^ BMEC tight junctions (occludin and claudin-5) upon 4 days’ treatment with isogenic MC-conditioned media show less discontinuous and frayed junctions (n = 6). (G and H) Isogenic BMECs show increased percentage of junction abnormalities upon treatment with disease MC-conditioned media (n = 6). Nuclei were stained with DAPI; scale bar, 100 μm. Results are presented as mean ± SD of n independent experiments. ^∗^p < 0.05, ^∗∗^p < 0.01, ^∗∗∗^p < 0.001, and ^∗∗∗∗^p < 0.0001; ns (not significant). Statistical analysis was performed using 2-way ANOVA with Tukey’s multiple-comparison test.

The integrity of tight junctions is essential for the BBB properties of brain ECs (Nitta et al., 2003; Pan et al., 2017). Thus, to assess if tight junctions are affected in *COL4A1*/*A2* hiPSC-derived BMECs, we performed immunostaining analysis for the tight junction proteins occludin and claudin-5 (Figure 3A). We observed striking discontinuities in occludin staining (Figure 3A, white arrow) and frayed junctions evident with claudin-5 staining (Figure 3A, white arrowhead) in *COL4A1*^*G755R*^ and *COL4A2*^*G702D*^ BMECs cultured alone. These abnormalities were significantly more frequently in the mutant lines compared with controls (Figure 3B). Moreover, this was associated with reduced occludin and claudin-5 total protein levels (Figures 3C and 3D).

To independently validate these findings and exclude that they were due to the differentiation protocol, we adopted an alternative endothelial differentiation protocol to generate hiPSC-derived ECs (iECs; Figure S3E) (Orlova et al., 2014a, 2014b). These iECs were characterized for expression of specific markers at mRNA levels by quantitative real-time PCR compared with human umbilical vein ECs (HUVECs) as positive control (Figure S3F) and flow cytometry (Figure S3G) and were found to have increased discontinued/frayed junctions as well as lower levels of occludin and claudin-5 proteins in disease lines versus controls (Figures S4A–S4D), validating our findings in BMECs.

To assess if the levels and the distribution of occludin and claudin-5 in ECs is regulated by the MC secretome, *COL4A1*^*G755R*^ and *COL4A2*^*G702D*^ hiPSC BMECs were treated with isogenic MC-conditioned media for 4 days prior to immunostaining (Figure 3E). Notably, treatment with isogenic MC media significantly improved the presence of discontinuous and frayed junctions (Figure 3F). Conversely, a greater number of discontinuous and frayed junctions were observed when both isogenic BMEC clones were treated with conditioned media from mutant *COL4A1*/*A2* MC (Figures 3G and 3H). These data clearly support that COL4 SVD includes tight junction defects in ECs that are determined at least in part by a paracrine effect exerted by the MCs.

### Transcriptomic analysis highlights ECM abnormalities in COL4A1/A2 MC lines

**Figure 4:**
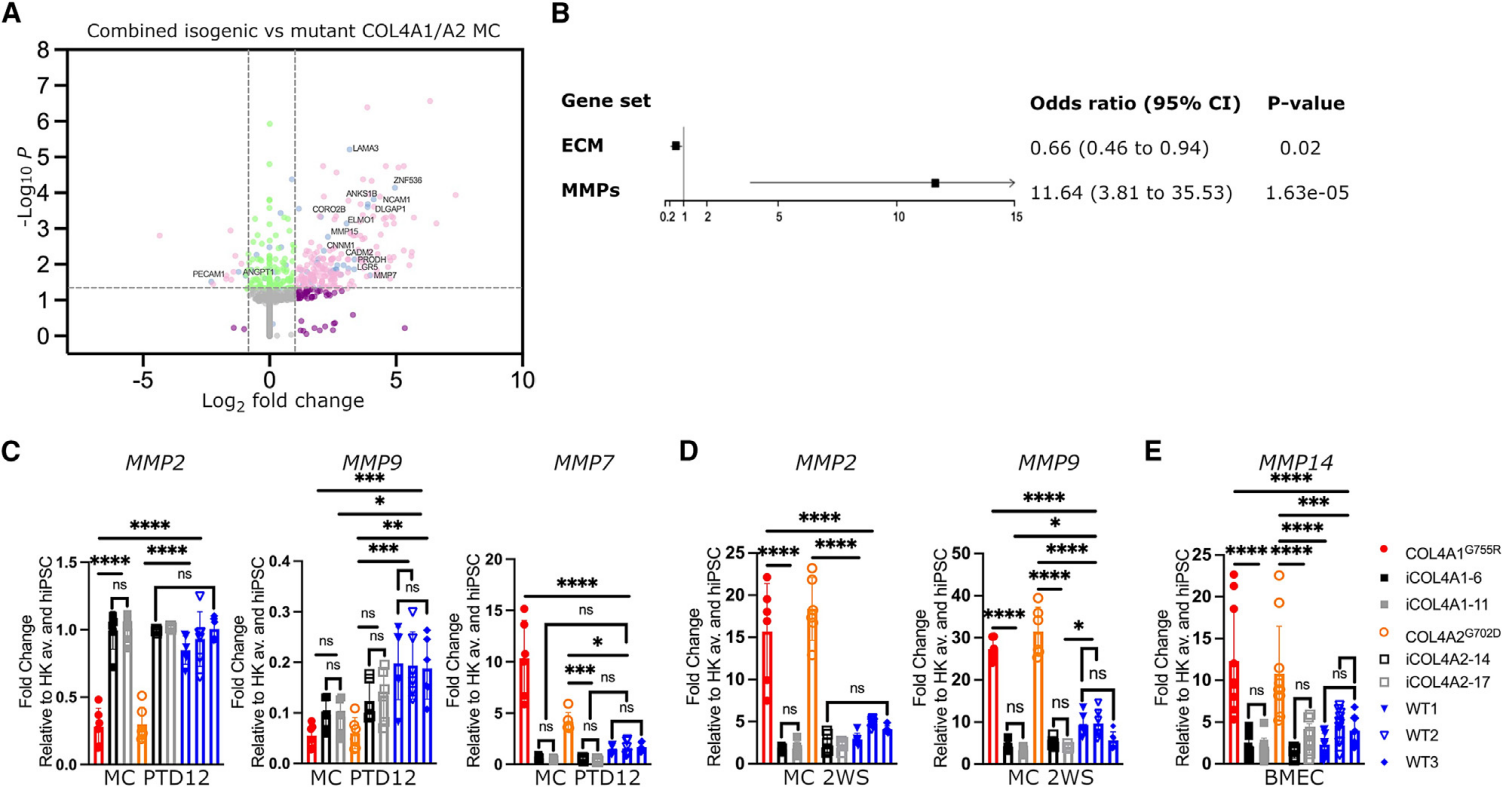
Transcriptomic analysis shows ECM abnormalities in COL4A1/A2 MC lines and MMP upregulation (A and B) Volcano plot depicting differentially expressed genes in combined COL4A1^*G755R*^ and COL4A2^*G702D*^ compared with isogenic MCs (A); matrisome proteins with larger fold changes are labeled, and (B) forest plot shows significant enrichment for ECM and MMPs in diseased MC. (C and D) quantitative real-time PCR analysis performed at PTD12 and 2WS shows biphasic expression for *MMP2* at mRNA levels in COL4A1^*G755R*^ and COL4A2^*G702D*^ MCs and higher levels for MMP7 and MMP9 at PTD12 and 2WS, respectively, in COL4A1^*G755R*^ and COL4A2^*G702D*^ MCs compared with the isogenic and WT controls (n = 6). (E) *MMP14* mRNA was found to be higher in COL4A1/A2 compared with isogenic and WT hiPSC BMEC lines (n = 10). Results are presented as mean ± SD of n independent experiments. ^∗^p < 0.05, ^∗∗^p < 0.01, ^∗∗∗^p < 0.001, and ^∗∗∗∗^p < 0.0001; ns (not significant). Statistical analysis was performed using 2-way ANOVA with Tukey’s multiple-comparison test.

To identify potential mediators of the MC paracrine effects reported above, we performed a transcriptomic analysis on *COL4A1*^*G755R*^ and *COL4A2*^*G702D*^ and corresponding isogenic hiPSC MCs in culture in serum containing media for a week (Figure 4A). From the bulk RNA sequencing (RNA-seq) data, we identified 374 differentially expressed genes (DEGs). No significant difference was observed for *COL4A1* and *COL4A2* mRNA levels between disease and control lines. Importantly, it emerged that 56 DEGs were ECM proteins, and that matrix metalloproteinases (MMPs) were among the proteins misregulated (Figure 4B; Tables S5 and S6). It is known that changes in MMPs levels are associated with barrier disruption and stroke (Candelario-Jalil et al., 2011; Clark et al., 1997; Wallin et al., 2017). To validate the transcriptomics findings, we perform quantitative real-time PCR in early MCs (PTD12) and late MCs (2WS) to profile MMP genes expression (Figures 4C and 4D). We observed a biphasic expression for *MMP2*, which appears to be downregulated at PTD12 and upregulated at the late stage (2WS). *MMP9* mRNA levels were also found to be upregulated in both *COL4A1* and *COL4A2* MCs at the late stage (Figure 4D). *MMP7* shows high expression levels at PTD12 (Figure 4C). In addition, we also found a significant increase in *MMP14* mRNA levels in *COL4A1*/*A2* BMECs (Figure 4E). Interestingly, MMP14, which activates pro-MMP2, was also previously reported to be upregulated in aorta of mice with a *Col4a1* glycine mutation (*Col4a1*^+/SVC^ G1064D) that is a well-established model of Col4a1-associated SVD (Figures S5A and S5B) (van Agtmael et al., 2005; Jones et al., 2016, 2019). The *MMP14* increase was also validated at protein levels in *COL4A1*/*A2* BMEC and iECs (Figures S5C and S5D). These data clearly support that the ECM and MMPs are dysregulated in *COL4A1*/*A2* MCs.

### MMP inhibition rescues phenotypic alterations, ECM, and tight junction defects

**Figure 5:**
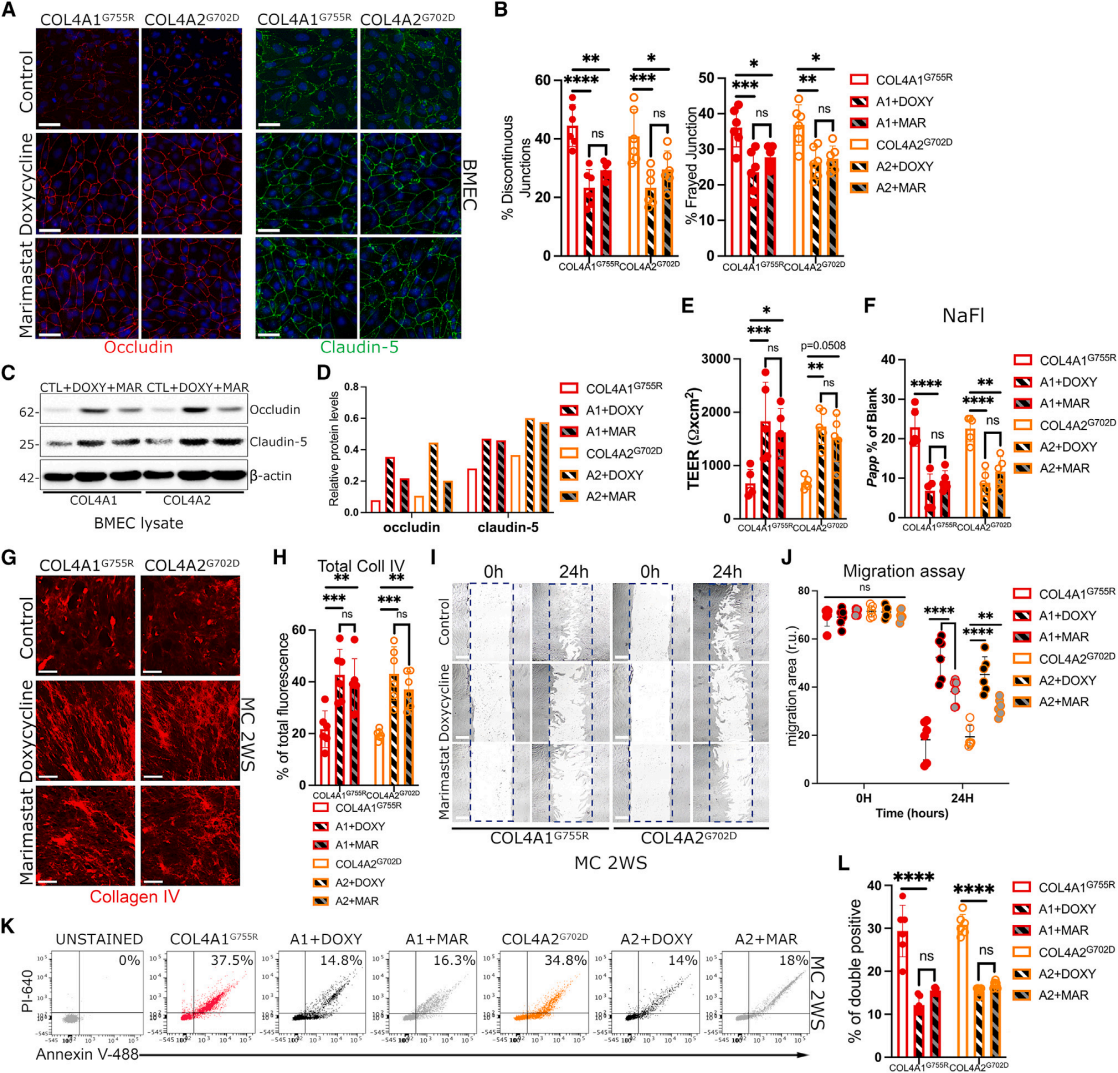
Doxycycline (DOXY) treatment ameliorates tight junction abnormalities and reverts COL4A1/A2 MC collagen IV defect and phenotypic changes (A and B) Immunostaining analysis quantification of occludin and claudin-5 in COL4A1^*G755R*^ and COL4A2^*G702D*^ BMECs treated with DOXY or marimastat (MAR) for 4 days shows lower percentage of tight junction abnormalities (discontinuous and frayed junctions) compared with untreated control (DMSO) (n = 6). (C and D) Protein blot analysis and band quantification show increased occludin and claudin-5 levels upon treatment with DOXY (+DOXY) and MAR (+MAR) (n = 2). β-Actin was used as loading control. (E and F) DOXY and MAR treatments improve both TEER (E) and NaFl (F) readouts in *COL4A1*/*A2* mutant BMECs compared with untreated controls (n = 6). (G) Immunostaining analysis of collagen IV in the decellularized ECM of *COL4A1*^*G755R*^ and *COL4A2*^*G702D*^ MCs at late stage (2WS) upon 4 days treatment with DOXY (10 μM) or MAR (1 μM), and total fluorescence quantification (H) show higher fluorescence in ECM compared with control (n = 6). (I and J) Representative image of scratch assay for *COL4A1*/*A2* hiPSC-derived MC control (DMSO), DOXY treated and MAR treated at 0 and 24 h (I), and scratch area quantification (J) show lower migration rate upon treatment with DOXY or MAR compared with controls (n = 6). (K and L) DOXY (+DOXY) and MAR (+MAR) treatments improve apoptotic levels in *COL4A1*^*G755R*^ and *COL4A2*^*G702D*^ MCs at 2WS compared with untreated (n = 5). Nuclei were stained with DAPI; scale bar, 100 μm. Results are presented as mean ± SD of n independent experiments. ^∗^p < 0.05, ^∗∗^p < 0.01, ^∗∗∗^p < 0.001, and ^∗∗∗∗^p < 0.0001; ns (not significant). Statistical analysis was performed using 2-way ANOVA with Tukey’s multiple-comparison test.

Because MMPs are important for matrix remodeling, including collagens, and because they also target tight junctions for degradation, we hypothesized that MMPs could mediate the *COL4A1*/*A2* ECM phenotype seen in our *in vitro* model. Thus, we proceeded to treat *COL4A1*/*A2* hiPSC-derived BMECs with the pan-MMP inhibitor doxycycline (DOXY), which appears to successfully represses MMP2 and MMP9 activity after 72 h treatment by zymography (Figure S5E). However, as DOXY is a broad-spectrum MMP inhibitor with potentially significant side effects, we also tested in our system a small-molecule inhibitor, marimastat (MAR), which specifically targets the MMPs seen dysregulated in our model (including MMP2, MMP9, MMP14, and MMP7). Upon 4 days’ treatment with 8 μM DOXY or 1 μM MAR, disease BMECs stained for occludin and claudin-5 show a significant reduction of discontinuous and frayed junction compared with control (DMSO) (Figures 5A and 5B). Similar effects were seen in iECs treated with DOXY or MAR (Figures S5F and S5G).

In addition, treatment with DOXY or MAR appeared to increase total occludin and claudin-5 protein levels by western blotting (Figures 5C and 5D). Remarkably, both DOXY and MAR treatment benefited on BMEC barrier properties, as evidenced by significantly increasing TEER values (Figure 5E) and reducing the NaFl permeability percentage (Figure 5F).

We also looked at the effect of inhibiting MMPs by DOXY and MAR treatments on collagen IV deposition in *COL4A1*/*A2* hiPSC-derived MCs, and we found that collagen IV fluorescence levels detected by immunostaining in the *COL4A1*/*A2* decellularized ECM increased upon treatments compared with controls (Figures 5G and 5H). DOXY- and MAR-treated disease MCs also show a significant decreased migration rate at 24 h (Figures 5I and 5J) and lower apoptotic levels (Figures 5K and 5L) comparable with controls (Figure 1G). These data establish a role for ECM remodeling due to MMPs caused by *COL4A1*/*A2* mutations and provide *in vitro* evidence that modulating specific MMPs may represent therapeutic targets for SVD.

## Discussion

There is a critical need to develop new models relevant to human SVD to provide mechanistic insights as well as a foundation to test potential treatments for this debilitating disorder. To address this, we characterized a novel *in vitro* model of human SVD produced by differentiating iPSCs generated from patients with *COL4A1*- or *COL4A2* SVD-related mutations into MCs.

We used *COL4A1*/*A2* patient-derived hiPSC MCs in a co-culture system with BMECs to mimic the changes seen in patients’ small vessels and to investigate underlying pathological mechanisms. First, we observed increased expression of smooth muscle cells markers, such as *CNN1* and *ACTA2*, in *COL4A1*/*A2* MCs at late stage of differentiation, which may suggest hypermuscularization, as previously shown in a Col4a1 mouse model (Ratelade et al., 2020). Disease MCs also showed an ECM defect, including lower levels of extracellular collagen IV, in agreement with previous findings from patient cells, indicating that SNPs in the triple-helix-forming domain are likely to affect the protein conformation, which in turn may destabilize collagen IV deposition in the ECM (Jeanne et al., 2015; Murray et al., 2014). We also determined phenotypic changes in disease MCs, including increased migration and apoptotic rates that parallel previous studies using primary patient fibroblasts (Murray et al., 2014). A loss of MCs has been reported before and could be caused by several mechanisms, including ECM remodeling and endoplasmic reticular stress (Ratelade et al., 2018, 2020).

Given the key strategic location of the ECM at the interface between blood and brain, a central aim of the study was to determine where COL4 disease influences barrier-related properties. Interestingly, *COL4A1*/*A2* patient-derived MCs exerted a detrimental effect on the endothelial barrier functions by a paracrine effect, evidenced by our Transwell setup and with MC-conditioned media treatment.

In this study, for the first time, we provided insight into the transcriptional features of *COL4A1*/*A2* patient-derived MCs. Strikingly 15% of changes affected ECM proteins, including MMPs. Collagen IV is a substrate for the proteolytic activity of the gelatinases MMP2 and MMP9 and the matrilysin MMP7. Increased MMP2 and MMP9 expression has been associated with breakdown of collagen type IV in both human and animal models (Roach et al., 2002; Rosell et al., 2008), as well as with degradation and cellular rearrangement of the endothelial tight junctions (Bauer et al., 2010; Liu et al., 2012; Yang et al., 2007). Recently, MMP7 has been found to correlate with BBB dysfunction following traumatic brain injury (Nichols et al., 2021). Moreover, MMPs are known to play a role in smooth muscle migratory behavior and may facilitate MC migration in our *COL4A1*/*A2* model by promoting ECM proteins proteolysis (Underly et al., 2017). Interestingly, we observed a biphasic change for *MMP2* mRNA with expression levels increasing at later differentiation stage, which corresponds with greater ECM deposition. This suggests that abnormal collagen IV deposition may contribute to higher MMP activity, which in turn could lead to increased cell death seen in our disease models.

These findings suggest MMPs could play a role in the ECM alterations in *COL4A1*/*A2*-related SVD and could present a novel therapeutic opportunity. In support of this, targeting MMPs using the pan-MMP inhibitor DOXY partially rescued the disease MC phenotypes, including promoting collagen IV extracellular levels, reducing migration and apoptotic levels, and improving BMEC/iEC tight junction abnormalities. In other studies, DOXY was shown to reduced vascular remodeling and damage induced by cerebral ischemia in a stroke animal model, the stroke-prone spontaneously hypertensive rats (Pires et al., 2011). However, DOXY is a broad-spectrum MMP inhibitor with potentially significant side effects. For this reason, we tested the small-molecule inhibitor MAR, which specifically targets the MMPs seen dysregulated in our models. MAR was the first MMP inhibitor to be tested in clinical trials and is now used for patients with different types of cancer (Thomas and Steward, 2005). Importantly, it was well tolerated by patients with short-term treatment.

Overall, major strengths of this work are as follows: (1) we generated a new hiPSC-derived disease model for SVD, which replicates phenotypic changes observed in patients and *Col4a1* animal model, including ECM abnormalities, and (2) this disease-relevant model can be used as new tool for the analysis of signaling pathways to identify therapeutic targets, such as specific MMP, and (3) to screen and test for potential drugs against SVD.

Our work has limitations. First, the *COL4A2* hiPSC line was generated from the asymptomatic father of the patient. Previously, it has been shown that the father’s fibroblasts lack some of the properties seen in the patient’s fibroblasts. However, in our model, *COL4A2* phenotypic changes are comparable with the *COL4A1* line (symptomatic), and this may be due to the use of relevant cell types to investigate these changes.

Second, generating representative brain ECs that possess endothelial identity while replicating the BBB properties, including elevated TEER and small-molecule low permeability, has been a challenge highlighted in recent hiPSC work (Lu et al., 2021). We initially used the protocol of Hollman et al., which originated from the Lippman lab (Hollmann et al., 2017). These cells display high TEER, but they do also express epithelium-related genes and lack angiogenic properties (Lu et al., 2021). In view of these limitations, we then successfully validated our results using a generic endothelial protocol (Orlova et al., 2014b), but this lacks barrier-like functions. Further research is required to improve the current protocols for generation of BMECs, on the basis of the emerging understanding of the BBB from single-cell sequencing studies (Garcia et al., 2022).

In conclusion, our novel hiPSC-derived MC model of *COL4A1*/*A2* mutations supports a key role of the ECM in SVD and suggests that targeting ECM-related proteins such as MMPs may be a promising potential therapeutic option.

## Experimental procedures

### Resource availability

#### Corresponding author

Further information and requests for resources and reagents should be directed to and will be fulfilled by the corresponding author, Alessandra Granata (ag686@cam.ac.uk).

#### Materials availability

This study did not generate new unique reagents. Materials are listed in supplemental experimental procedures in the supplemental information and can be requested from the corresponding author.

#### Data and code availability

The RNA-seq analysis data generated during this study have been deposited on Apollo - University of Cambridge Repository: https://doi.org/10.17863/CAM.100127 and is publicly available.

### Experimental methods

#### HiPSC culture

All the hiPSC lines use for this study are listed in Table S1. Full culture condition and medium formulation can be found in the supplemental information.

#### HiPSC differentiation into MCs

hiPSCs were differentiated into MCs of neural crest origin using a previously described protocol (Cheung et al., 2012; Serrano et al., 2019). Full culture condition and medium formulation can be found in the supplemental information.

#### HiPSC differentiation into BMECs and iECs

hiPSCs were differentiated to BMECs as previously described, with minor modifications (Hollmann et al., 2017). iECs were differentiated using a previously reported protocol with minor modifications (Orlova et al., 2014b). Full culture conditions and medium formulation can be found in the supplemental information.

#### Transwell co-culture

Either 12- or 24-well Transwells (Corning 0.4 μm pore; Sigma-Aldrich) were coated on the apical and basolateral side with collagen IV/fibronectin. hiPSC MCs were dissociated with TrypLE and seeded onto the plate bottom of the Transwell coated with 0.1% gelatin. After incubation for 1 h, hiPSC BMECs were dissociated and the seeded onto the apical side. The next day, Transwells with BMECs with(out) MCs were maintained without any further medium changes for up to 6 days before analyses.

##### Paracrine

MCs were serum starved for 4 days. At day 5, the MC serum-starved conditioned media was added to BMEC seeded onto collagen IV/fibronectin coated 24-well Transwells for TEER and NaFl analyses or 24-well plates for immunostaining assay. The initial TEER measurement was taken after 24 h and afterward on a daily basis. For NaFl and immunostaining assays, BMECs were treated with condition media, refreshed every other day, for 6 days.

#### BMEC functional assays

##### TEER

TEER measurements were taken every 24 h, from day 1 to day 6 of subculture of BMECs onto Transwells using an EVOM2 Voltohmmeter/STX2 electrodes (World Precision Instruments). The STX2 electrode was positioned within the well and the resistance (Ω) was recorded three times to calculate the mean resistance. All values are given as Ω · cm^2^ after subtracting the resistance of an empty coated Transwell maintained in the same culture media (blank) and multiplying by the surface area (0.33 cm^2^), as described previously (Lee et al., 2018). TEER was expressed as peak value.

##### NaFl

At 2 days post-subculture of BMECs onto 24-well Transwells, spent medium was removed from the upper chamber of the Transwell and replaced with 600 μL NaFl (1 mg/mL; Sigma-Aldrich) diluted 1:100 in endothelial serum-free media with B27. Samples of 100 μL were taken from the basolateral side every two hours for eight hours. Raw fluorescence was measured with a TECAN Infinite M200 Pro plate reader (excitation wavelength 460 nm and emission wavelength 515 nm; gain of 50, 25 flashes; z-position 20,000). Quantification was represented as percentage of total fluorescence relative to empty coated Transwell (blank), as previously described (Lee et al., 2018).

#### DOXY and MAR treatments

hiPSC-derived MCs were treated with DOXY (10 μM; Sigma-Aldrich) or MAR (AstraZeneca; 1 μM in DMSO) in DMEM + 10% fetal bovine serum (FBS) for 4 days, with media change every other day, and then harvested for analyses. BMECs/iECs were treated with 8 μM DOXY or MAR (1 μM) in EC medium, with media change every other day, and collected at 24 and 72 h for zymography and at day 6 for immunostaining and western blotting analysis. TEER measurements were taken every 24 h from day 1 to day 6 of subculture of BMECs onto Transwells in media supplemented with DOXY or MAR. NaFl permeability assay was performed after 6 days of DOXY or MAR treatment.

#### RNA-seq

##### Sample preparation

Three sets (biological replicates) of hiPSC-derived MCs grown in DMEM + 10% serum for 1 week were harvested. Total RNA was isolated from cells using the RNeasy Mini Kit (QIAGEN). Upon ribosomal RNA depletion, libraries were prepared using a NEBNext RNA library Prep kit (Illumina). The samples were run on a Novaseq6000 S4 lane, and 150 bp paired-end reads were generated.

##### Data analysis

The resulting base call files were converted to fastq files using the bcl2fastq program. Alignment in STAR (version 2.7.10a) using a modified version of the ENCODE-DCC RNA-seq pipeline annotated using GENCODE version 39 (hg38) was performed (Dobin et al., 2013). Gene-level RNA expression quantification was performed using RSEM (Li et al., 2011).

Differential expression analyses were carried out using DESeq2 in R version 4.0.4 (Love et al., 2014). We specified a false discovery rate of 5% and applied a Bayesian shrinkage estimator to effect sizes using approximation of the posterior for individual coefficients. Results were visualized using the EnhancedVolcano package.

Enrichment of gene sets of interest was calculated using logistic regression. We used data from human samples to categorize genes associated with the ECM (Pokhilko et al., 2021) and MMPs (Table S5). Pathways enrichment analysis was performed using the Reactome (Table S6) (Wu and Haw, 2017). We chose a 5% false discovery rate (FDR) to indicate statistical significance.

#### Statistical analysis

Data, expressed as mean ± SD, were analyzed statistically using SPSS version 22.0. Unpaired Student’s t test for two-group comparisons or one-way ANOVA followed by least significantly different (LSD) multiple comparisons was performed using Prism version 9.00 (GraphPad Software, Inc.) to analyze the significant difference, which was indicated as ns (not significant; p > 0.05), ^∗^p < 0.05, ^∗∗^p < 0.01, ^∗∗∗^p < 0.001, and ^∗∗∗∗^p < 0.0001. The n noted in the figure legends represents the replicated number of biological experiments. All data are representative of at least three independent experiments.

## Supplemental information

Document S1. Figures S1–S5, Tables S1–S6, and supplemental experimental procedures

Document S2. Article plus supplemental information
