# Supplementary material for: A novel human iPSC model of COL4A1/A2 small vessel disease unveils a key pathogenic role of matrix metalloproteinases
Source: Stem Cell Reports. 2023 Nov 16;18(12):2386–99. doi: 10.1016/j.stemcr.2023.10.014 (PMC10724071; doi:10.1016/j.stemcr.2023.10.014)
Supplement: Document S1. Figures S1–S5, Tables S1–S6, and supplemental experimental procedures [file mmc1.pdf]

Stem Cell Reports, Volume *18*

Supplemental Information

A novel human iPSC model of COL4A1/A2 small vessel disease unveils a key pathogenic role of matrix metalloproteinases

Maha Al-Thani, Mary Goodwin-Trotman, Steven Bell, Krushangi Patel, Lauren K. Fleming, Catheline Vilain, Marc Abramowicz, Stuart M. Allan, Tao Wang, M. Zameel Cader, Karen Horsburgh, Tom Van Agtmael, Sanjay Sinha, Hugh S. Markus, and Alessandra Granata

Supplemental Information

Supplemental Figures and Legends

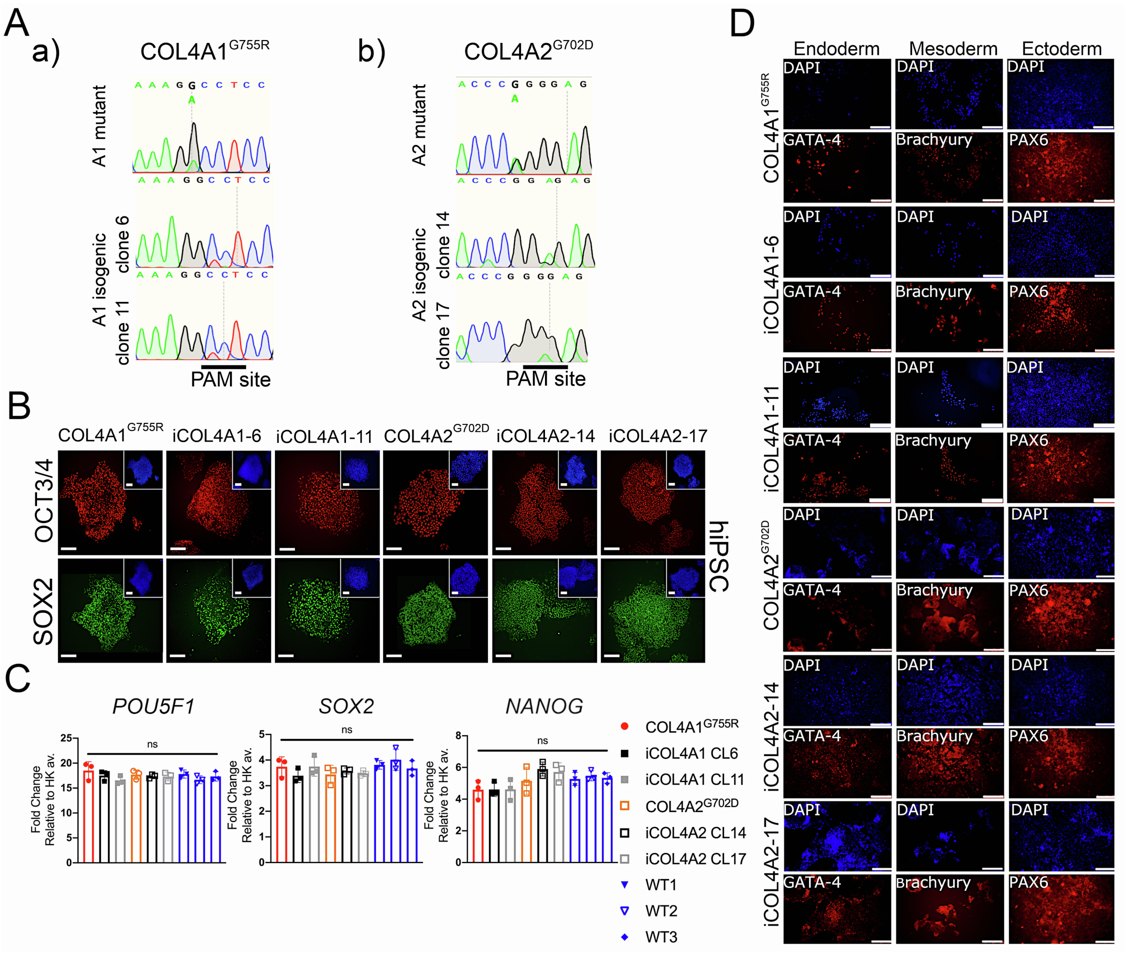

Figure S1. Characterization for COL4A1^{G755R}, COL4A2^{G702D} and isogenic hiPSC lines.

A) Sanger sequencing output for **(a)** *COL4A1* heterozygous mutation (G775R) and CRISPR-corrected isogenic A1 clone 6 and clone 11 and **(b)** *COL4A2* heterozygous mutation (G702D) and CRISPR-corrected isogenic A2 clone 14 and clone 17. **B)** Immunostaining analysis for hiPSC markers (OCT3/4 and SOX2) for *COL4A1^{G755R}*, *COL4A2^{G702D}* and isogenic A1 (iCOL4A1-6 and -11) and A2 (iCOL4A2-14 and -17); nuclei were stained with DAPI (insert); scale bar=100µm. **C)** Quantitative real-time PCR analysis for pluripotency markers expression (*POU5F1*, *SOX2* and *NANOG*); the results are presented as means ± SD of 3 independent experiments. **D)** Immunostaining analysis for each of the three germ layers (GATA-4, endoderm; BRACHYURY, mesoderm; PAX6, ectoderm); nuclei were stained with DAPI; scale bar=100µm. hiPSC= induced pluripotent stem cells. PAM site= protospacer adjacent motif sequence. The results are presented as means ± SD of 3 independent experiments; ns (not significant). Statistical analysis was performed by 2-way ANOVA with Tukey's multiple comparison test.

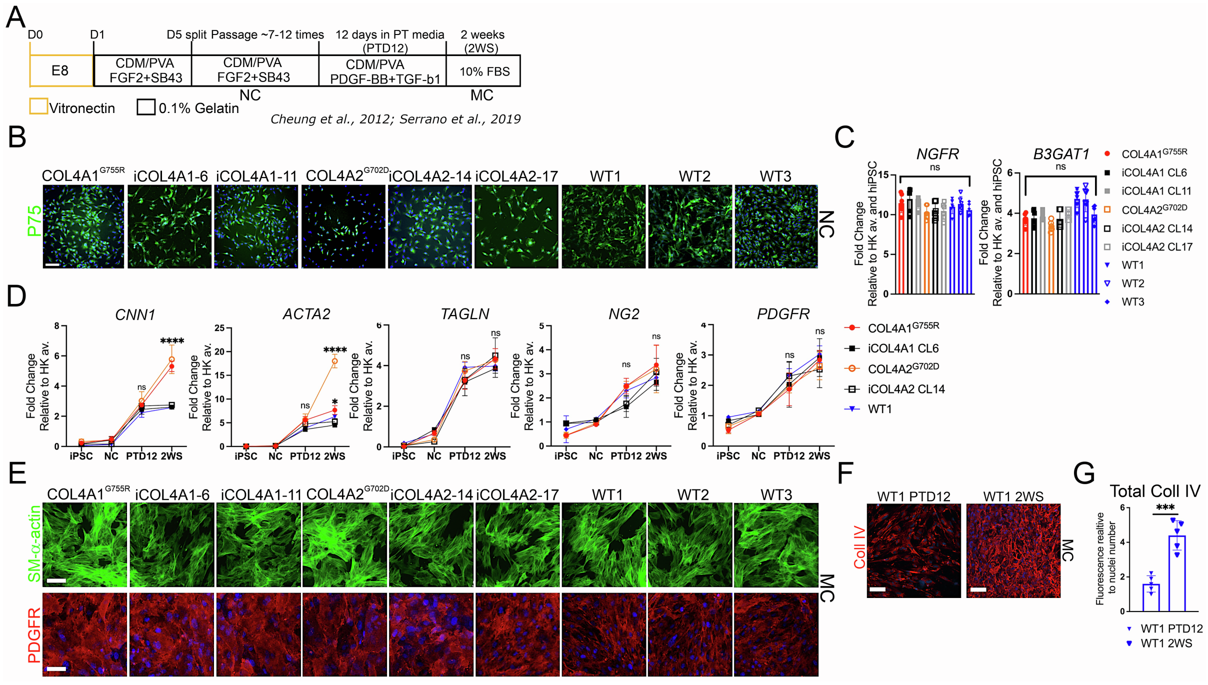

Figure S2. Neural-crest derived mural cells differentiation and characterization. **A)** Schematic of neural crest (NC) derived mural cells (MC) differentiation. Characterization of NC intermediate population by **(B)** immunostaining for specific marker (p75) and **(C)** quantitative real-time PCR for *NGFR* (P75) and *B3GAT1* (HNK1) for COL4A1^{G755R}, COL4A2^{G702D}, two isogenic sub-clones for A1 (iCOL4A1-CL6 and CL11) and A2 (iCOL4A2-CL14 and Cl-17) and three independent healthy controls (WT1, WT2 and WT3; see also **Table S1**) (n=6). **D)** Representative time-course quantitative real-time PCR of hiPSC-MC differentiation for specific markers: *CNN1, ACTA2, TAGLN, NG2, PDGFR* at hiPSC, NC and MC at day 12 of PDGFBB+TFG-β1 differentiation (PTD12) and after 2 weeks of culture in serum-containing media (2WS) for COL4A1/2, iCOL4A1-CL6, iCOL4A2-CL14 and WT1 (n=3). **E)** Immunostaining for smooth muscle alpha actin (SM α-actin) and PDGF Receptor Β (PDGFRB) in hiPSC-derived MC for COL4A1^{G755R}, COL4A2^{G702D}, 2 isogenic sub-clones and WT controls. **F)** Immunostaining for collagen IV in MC at early stage (PTD12) of differentiation and at late stage (2WS) and **(G)** quantification of n= 6 biological replicates. Nuclei were stained with DAPI; scale bar=100μm. NC=neural crest; MC=mural cells. The results are presented as means ± SD of n independent experiments; *P<0.05; ***P<0.001; ****P<0.0001; ns (not significant). Statistical analysis was performed by unpaired Student's t test for two-group comparisons or 2-way ANOVA with Tukey's multiple comparison test.

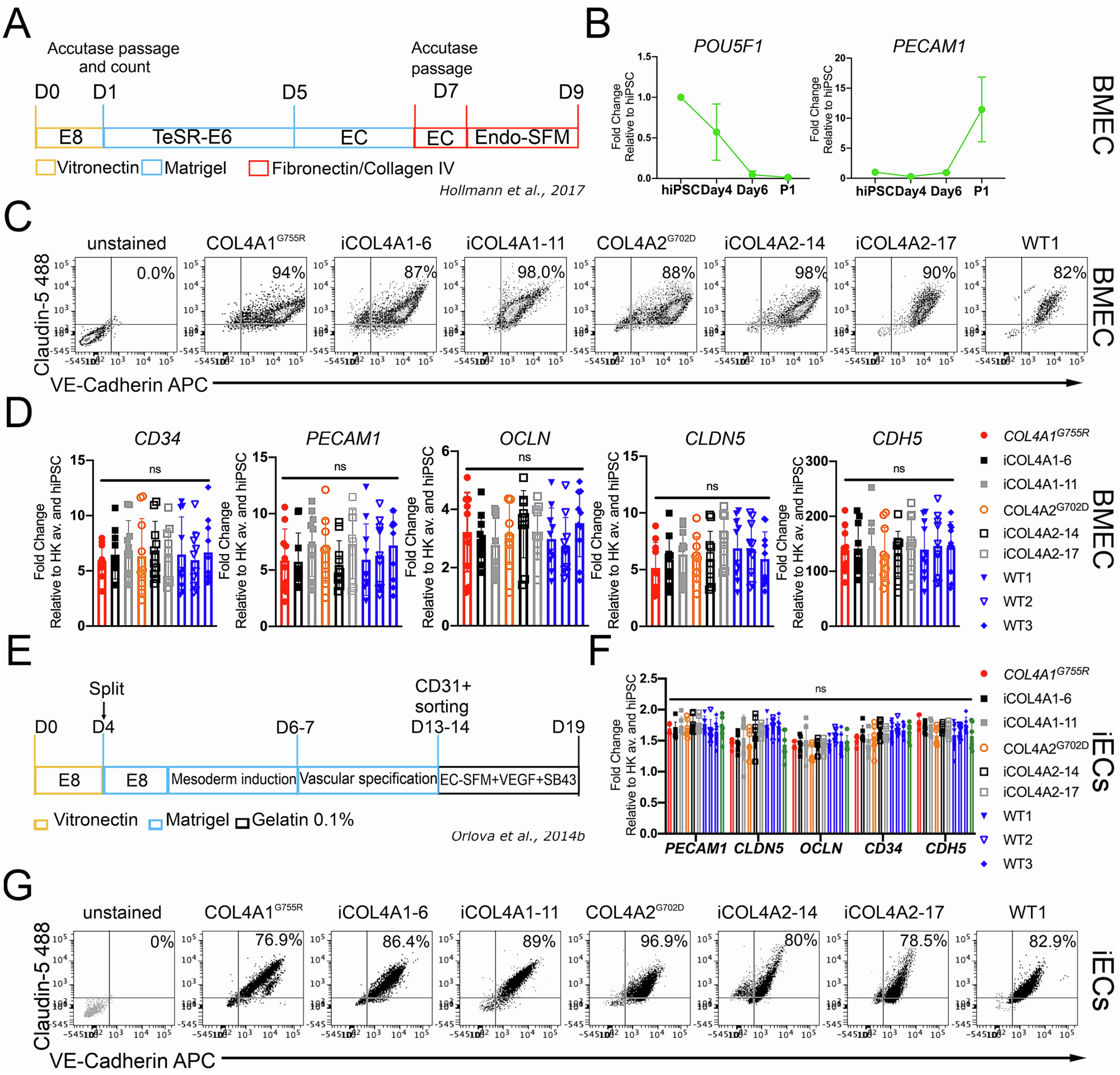

Figure S3. hiPSC-derived brain microvascular endothelial-like cells and endothelial cells differentiation and characterization.

A) Schematic of brain microvascular endothelial-like cells (BMEC) differentiation from hiPSC. **B)** Representative time-course quantitative real-time PCR of WT1 hiPSC-BMEC differentiation for pluripotent marker (*POU5F1*) and endothelial marker (*PECAM1*). **C)** Flow cytometric analysis of hiPSC-BMEC for VE-cadherin (APC conjugated) and claudin-5 (488 conjugated) for *COL4A1*^{G755R}, *COL4A2*^{G702D}, isogenic sub-clones and WT1. **D)** mRNA profile of hiPSC-BMEC by quantitative real-time PCR for specific markers (*CD34, PECAM1, OCLN, CDH5 and CLDN5*) (n=10). **E)** Schematic of hiPSC-endothelial cells (iECs) differentiation and characterization by **(F)** quantitative real-time PCR for endothelial markers: *PECAM1, CLDN5, OCLN, CD34* and *CDH5* for iECs lines and HUVEC (n=6) and **(G)** flow cytometry for VE-cadherin APC and claudin-5 488 double staining. HUVEC = Human umbilical vein endothelial cells. The results are presented as means ± SD of n independent experiments; ns (not significant).

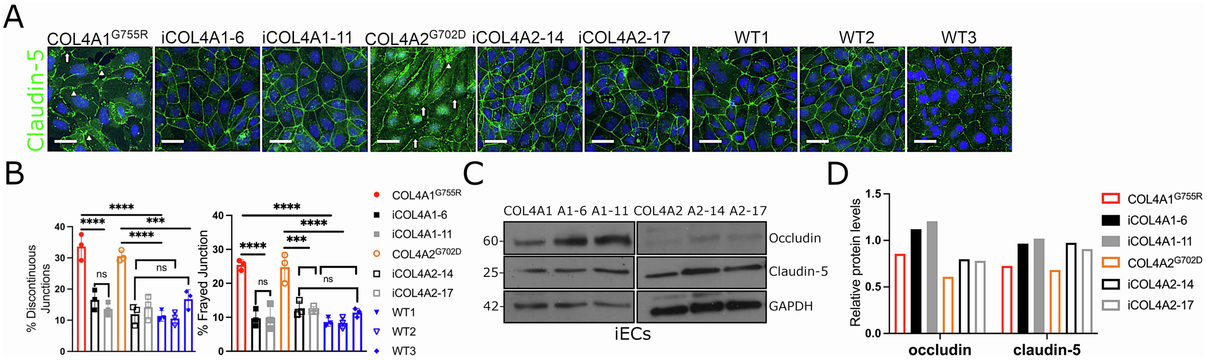

Figure S4. hiPSC-derived ECs showing tight junction abnormalities.
A-B) Immunostaining for claudin-5 shows increased discontinuity and frayed junctions in *COL4A1/A2* hiPSC-ECs compared to isogenic sub-clones and WT controls (n=3). Nuclei were stained with DAPI; scale bar=100µm. **C)** Western blotting for occludin and claudin-5 in iECs and (**D**) quantification relative to housekeeping (GAPDH) shows decreased protein levels in mutant lines compared to the isogenic control (representative blot of n=2). The results are presented as means ± SD of n independent experiments; ***P<0.001; ****P<0.0001; ns (not significant). Statistical analysis was performed by 2-way ANOVA with Tukey’s multiple comparison test.

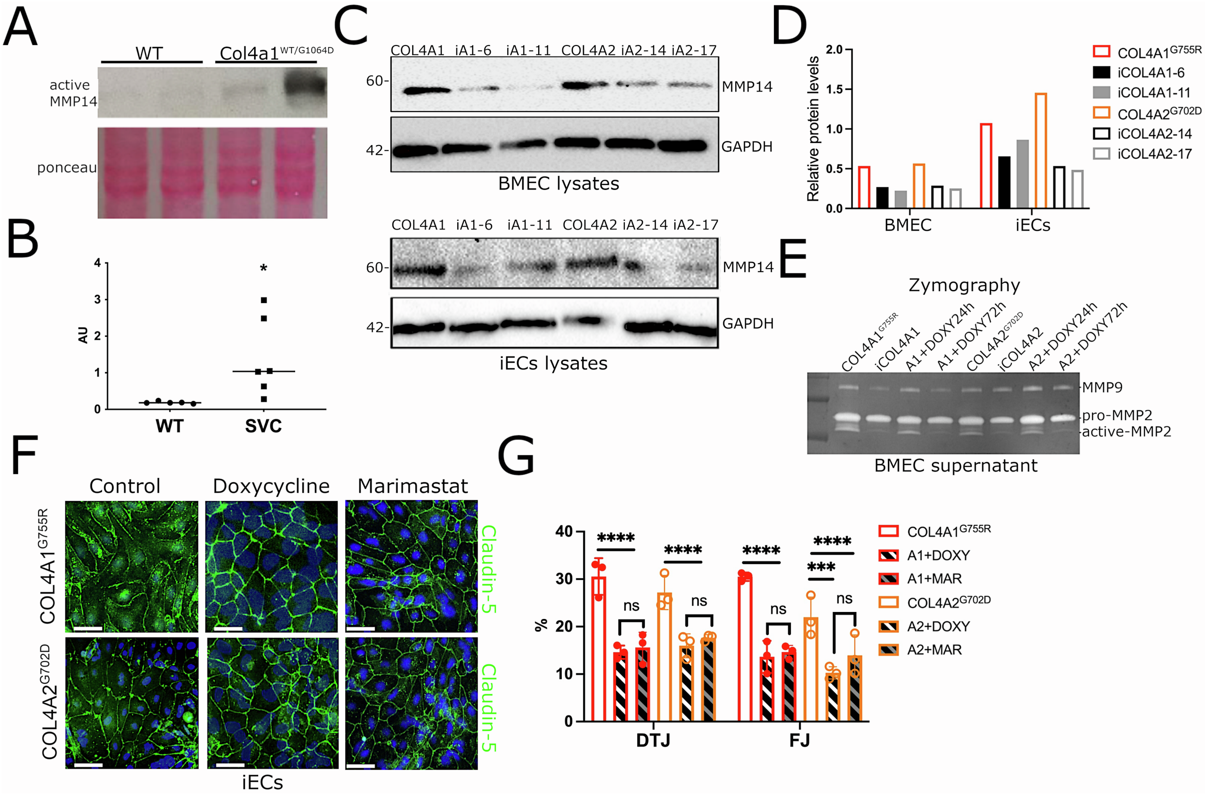

Figure S5. MMP14 is increased in Col4a1 mouse aorta and hiPSC-derived BMEC and iECs

A-B) Total Mmp14 protein level found higher in Col4a1 mice aorta (n=6) compared to WT mice (n=5). **C-D)** Protein blots and quantification showing MMP14 upregulation in *COL4A1/A2* BMEC and iECs compared to isogenic sub-clones (representative blots of n=4). **E)** Zymography analysis of BMEC supernatants show higher MMP9 and MMP2 activity levels in mutant *COL4A1/A2* compared to isogenics, which decrease in response to doxycycline (8 uM) mediated MMPs inhibition at 72 hours (DOXY72h) treatment. **F-G)** Discontinuity (DTJ) and frayed junctions (FJ) in *COL4A1/A2* iECs showed by staining for claudin-5 is reverted upon doxycycline and marimastat (MAR; n=3) treatment compared to control (DMSO). Nuclei were stained with DAPI; scale bar=100μm. The results are presented as means ± SD of n independent experiments *P<0.05; ***P<0.001; ****P<0.0001; ns (not significant). Statistical analysis was performed by 2-way ANOVA with Tukey's multiple comparison test.

Tables

Table S1. List of hiPSC lines used in this study.

Name	Vendor or Source	Sex and age	Individual	URL and Reference	Reprogram method/gene editing
WT1 (HPSI0414i-seru_7)	HIPSCI Consortium	F<br>65-69		https://www.hipsci.org/lines/#/lines/HPSI0414i-seru_7	Sendai virus
WT2 (HPSI0314i-sojd_3)	HIPSCI Consortium	F<br>45-49		https://www.hipsci.org/lines/#/lines/HPSI0314i-sojd_3	Sendai virus
WT3 (HPSI0214i-wibj_2)	HIPSCI Consortium	F<br>55-59		https://www.hipsci.org/lines/#/lines/HPSI0214i-wibj_2	Sendai virus
COL4A1^{G755R} Clone 4 and 5	iPS Core Facility, Cambridge	F<br>65	SVD patient	(Shah et al)	Sendai virus
COL4A2^{G702D}	-	M<br>75	Father of SVD patient	(Murray et al)	Sendai virus
iCOL4A1 clone 6 and 11	-	F<br>65		-	CRISPR/Cas9 edited
iCOL4A2 clone 14 and 17	-	M<br>75		-	CRISPR/Cas9 edited

Table S2. CRISPR sgRNA guide and ssODN related to Figure S1.

Gene/Mutation	Sequencing primers sequence 5'-3'	gRNA	Donor sequence 5'-3' (ssODN)<br>Corrected base<br>Mutated PAM
COL4A1^{G755R}	GCTTGAAAAGGGTTGAGCAG	CCGGCATTCCTGGCACACCC	G*A*C*TCAAAGGTTTGCCAGGTCTTCCCGGCATTCCTGGCACA CCC**G**G**A**GAGAAGGGGAGCATTGGGGTACCAGGCGTTCCTGGAGAAC*A*T*G
COL4A2^{G702D}	TCCAGTCCGTAAACAGGATTT	CGAAGCCUGGGAUUCCUCGG	G*C*C*TGATGTGGTTTGTGGTTTATTTGGTTATTTAGGTGCCAAAG**GT**CTCCGAGGAATCCCAGGCTTCGCAGGAGCTGATGGAGGAC*C*A*G

Table S3. Quantitative real-time PCR Primers set used in this study, related to Figure 1, Figure 4, Figure S1, Figure S2 and Figure S3.

**Gene Target**	**Forward Sequence 5'-3'**	**Reverse Sequence 5'-3'**
GAPDH	AACAGCCTCAAGATCATCAGC	GGATGATGTTCTGGAGAGCC
HMBS (PBGD)	GGAGCCATGTCTGGTAACGG	CCACGCGAATCACTCTCATCT
POU5F1 (OCT4)	AGGGCAAGCGATCAAGCA	GGAAAGGGACCGAGGAGTA
SOX2	ATGCACCGCTACGACGTGA	CTTTTGCACCCCTCCCATTT
NANOG	ACTAACATGAGTGTGGATCC	TCATCTTCACACGTCTTTCAG
PECAM1	CAGGCGCCGGGAGAAGTGAC	CGTCCAGTCCGGCAGGCTCT
CD34	CACAGGAGAAAGGCTGGGCGA	TGGCCGTTTCTGGAGGTGGC
OCLN	GGAGTGAACCCAACTGCTCA	CTCCTGGGGATCCACAACAC
CDH5	GGTCAAACTGCCCATACTTG	CGCAATAGACAAGGACATAACAC
CLDN5	CAGTACCGCAGGAAGAGGAG	ATCCCATGGCAAACAGAGAG
CHD5	CTCTGGGAGTGAGTGGAAGC	CCTGAGGATGATGGGAAAGA
MMP2	TCTCCTGACATTGACCTTGGC	CAAGGTGCTGGCTGAGTAGATC
MMP9	TTGACAGCGACAAGAAGTGG	GCCATTCACGTCGTCCTTAT
MMP14	CAGAGAAGGCACACAAACGA	CACTGGTGAGACAGGCTTGA
CNN1	GTCCACCCTCCTGGCTTT	AAACTTGTTGGTGCCCATCT
P75	ACAAGACCTCATAGCCAGCAC	CTGTTGGCTCCTTGCTTGTTC
CSPG4 (NG2)	TTCCAGCTGAGCATGTCTGA	TCCTCCCGATCTGAAACCAC
PDGFRB	GCTTAAATCCACAGCCCGCA	AGGTAGTCCACCAGGTCTC

Table S4. Primary antibodies list used in this study, related to Figure1, Figure 3, Figure 5, Figure S1, Figure S2, Figure S3, Figure S4 and Figure S5.

Target antigen	Species	Supplier	Catalogue n.	Use
OCT3/4	Mouse	Santa Cruz	SC-5279	Immunofluorescence
SOX2	Mouse	Abcam	sc-21705	Immunofluorescence
TRA-1-60	Rabbit	R&D Systems	AF2018-SP	Immunofluorescence
GATA-4	Mouse	Santa Cruz	sc-25310	Immunofluorescence
Brachyury	Mouse	Santa Cruz	sc-166962	Immunofluorescence
Occludin	Mouse	Thermo Fisher	331500	Immunofluorescence Western blotting
claudin-5	Rabbit	Abcam	ab15106	Immunofluorescence Western blotting
P75	Rabbit	Abcam	ab8874	Immunofluorescence
Smooth Muscle Actin	Mouse	Agilent	M085101-2	Immunofluorescence
SM22	Rabbit	Abcam	ab14106	Immunofluorescence
Calponin	Mouse	Sigma-Aldrich	C-2687	Immunofluorescence
NG2	Rabbit	Sigma-Aldrich	AB5320	Immunofluorescence
PDGF Receptor beta	Rabbit	Cell Signaling	3169	Immunofluorescence
Collagen IV	Rabbit	Abcam	ab6586	Immunofluorescence
claudin-5 pre-conjugated AF488	Mouse	Thermo Fisher	352588	Flow cytometry
CD144 (VE-cadherin) APC-conjugated	Mouse	Thermo Fisher	17-1441-80	Flow cytometry
IgG1 Isotype Control FITC-conjugated	Mouse	Thermo Fisher	GM4992	Flow cytometry
IgG1kappa Isotype Control APC-conjugated	Mouse	R&D Systems	IC002A	Flow cytometry
Annexin V-488	-	Life technologies	V13241	Flow cytometry
Propidium Iodide (PI)	-	Life technologies		Flow cytometry
β-Actin	Mouse	Sigma-Aldrich	A1978	Western blotting
MMP14	Rabbit	Abcam	ab51074	Western blotting
GAPDH	Mouse	Abcam	Ab8245	Western blotting

Table S5. List of identified ECM differentially expressed genes (DEGs) in *COL4A1/A2* vs isogenic MC, related to Figure 4.

GENE	log2FoldChange	LOG FDR	ECM
ZNF536	4.95529102016929	4.14E+00	1
NCAM1	4.11174320070316	3.82E+00	1
MMP7	3.97688493423821	1.69E+00	1
ANKS1B	3.88805632439505	3.68E+00	1
DLGAP1	3.87359008701633	3.60E+00	1
PRODH	3.35816928153035	2.14E+00	1
LGR5	3.34382833835286	1.86E+00	1
LAMA3	3.16127049352719	5.22E+00	1
CADM2	3.11452675789334	1.88E+00	1
ELMO1	3.04557087794061	3.14E+00	1
KCNA2	2.92787394337378	1.98E+00	1
ABCB1	2.80072080613374	1.71E+00	1
GPR158	2.67672768137061	1.98E+00	1
CNTN1	2.64145308178325	1.86E+00	1
LIMCH1	2.6119812320724	1.86E+00	1
ALDH1L1	2.52924831679259	1.83E+00	1
TAGLN3	2.40089796135524	1.79E+00	1
MMP15	2.3017540760174	2.77E+00	1
CNNM1	2.14528940341127	2.38E+00	1
ATP1A2	2.07014110537751	1.37644255215566	1
SH3GL2	2.02674427698518	3.32709887309457	1
SH3GL3	1.96493467263382	1.33503127079419	1
AP3B2	1.91281198134199	2.14678343169756	1
LONRF2	1.86610002336086	2.04730604450956	1
LAMA1	1.74955128715685	1.58968870650893	1
MMP24	1.72542160915493	1.36283160917736	1
DOCK3	1.7093082692694	1.66463712498211	1
SBSPON	1.62860413058366	1.50518527608545	1
CLU	1.61895548373455	1.58564688586111	1
PPP1R9A	1.48785411210524	1.79003207873103	1
GAD1	1.40416565564864	1.40405181192663	1
COL4A6	1.37761556652196	1.33709183759027	1
SPTBN2	1.34273321873787	1.46793184590654	1
DCLK1	1.30508213285984	1.40108978598792	1
LGI3	1.23212136740195	1.33709183759027	1
RBFOX3	1.2301953062032	1.84310680196793	1
CORO2B	1.15992146465967	3.55721027028743	1
ANK2	0.882039273617303	4.37869352390178	1
CASK	0.837906889789319	1.54614802467899	1
NBEA	0.708993913453233	1.326775846766	1
ASAH1	0.643114654921668	1.97718682074561	1
ITPR2	0.456699641255665	2.47578039450843	1
DNAJC9	0.431760286525865	3.43480323008621	1
PHIP	0.405822504148944	1.32937067329303	1
PRDX6	0.383723822	1.30369531898155	1
ATP6V1F	0.315755603918921	1.58314577498045	1
NFS1	0.220249262501158	1.78682423843528	1
SHROOM2	0.206055478537332	0.981122182373769	1
STX12	0.123459940152881	0.330434873025838	1
RTN1	0.0799153826051542	1.43005411626271	1
MYOF	0.275804043	1.66463713293907	1
SELENBP1	-2.96E-07	1.44731570190812	1

**LAMP5**	1.56E-06	1.85572241272765	1
**ELN**	-1.52E-06	2.47578039450843	1
**ICAM5**	-0.175606968002257	1.60858811632863	1
**MMP2**	-0.716063799342786	1.84392735543628	1
**MYL9**	-0.948504723731799	1.68499291515606	1
**ANGTP1**	-1.4204553	1.79003208	1
**PECAM1**	-2.30097954443201	1.51805900595662	1

Table S6. The Reactome pathways analysis of the identified ECM DEGs, related to Figure 4.

Pathway identifier	Pathway name	pValue	Gene ID
**R-HSA-1592389**	Activation of Matrix Metalloproteinases	1.7E-08	MMP24;MMP7;MMP15;MMP2
**R-HSA-1474244**	Extracellular matrix organization	3.3E-08	MMP24;MMP7;MMP15;LAMA1;MMP2;LAMA3; COL4A6;PECAM1;CASK;NCAM1
**R-HSA-1474228**	Degradation of the extracellular matrix	4.37E-07	MMP24;MMP7;MMP15;MMP2;LAMA3;COL4A6
**R-HSA-1442490**	Collagen degradation	8.81E-07	MMP7;MMP15;MMP2;COL4A6
**R-HSA-2022090**	Assembly of collagen fibrils and other multimeric structures	1.57E-05	MMP7;LAMA3;COL4A6
**R-HSA-373760**	L1CAM interactions	3.17E-05	LAMA1;CNTN1;NCAM1;ANK2;SH3GL2;SPTBN2
**R-HSA-1474290**	Collagen formation	1.24E-04	MMP7;LAMA3;COL4A6
**R-HSA-3000171**	Non-integrin membrane-ECM interactions	1.91E-04	LAMA1;LAMA3;COL4A6;CASK
**R-HSA-9022927**	MECP2 regulates transcription of genes involved in GABA signaling	3.68E-04	GAD1
**R-HSA-3000157**	Laminin interactions	4.16E-04	LAMA1;LAMA3;COL4A6
**R-HSA-6785807**	Interleukin-4 and Interleukin-13 signaling	4.33E-04	MMP7;MMP2
**R-HSA-3000178**	ECM proteoglycans	5.04E-04	LAMA1;LAMA3;COL4A6;NCAM1
**R-HSA-6806834**	Signaling by MET	7.52E-04	SH3GL3;LAMA1;LAMA3;SH3GL2
**R-HSA-9009391**	Extra-nuclear estrogen signaling	0.00176009 9	MMP7;MMP2
**R-HSA-70688**	Proline catabolism	0.00195505 1	PRODH
**R-HSA-9006934**	Signaling by Receptor Tyrosine Kinases	0.00217141 8	SH3GL3;DOCK3;LAMA1;LAMA3;ELMO1;ITPR2; SH3GL2;ATP6V1F
**R-HSA-2214320**	Anchoring fibril formation	0.00223768 5	LAMA3;COL4A6
**R-HSA-5578775**	Ion homeostasis	0.00328660 5	ITPR2;ATP1A2
**R-HSA-8875360**	InlB-mediated entry of Listeria monocytogenes into host cell	0.00354800 8	SH3GL3;SH3GL2
**R-HSA-422475**	Axon guidance	0.00534499 4	LAMA1;MMP2;CNTN1;NCAM1;ANK2; SH3GL2;SPTBN2
**R-HSA-6807004**	Negative regulation of MET activity	0.00603478 6	SH3GL3;SH3GL2
**R-HSA-8876384**	Listeria monocytogenes entry into host cells	0.00699757 5	SH3GL3;SH3GL2
**R-HSA-9675108**	Nervous system development	0.00756573 7	LAMA1;MMP2;CNTN1;NCAM1;ANK2;SH3GL2;SPTBN2
**R-HSA-8874081**	MET activates PTK2 signaling	0.00968552 2	LAMA1;LAMA3
**R-HSA-445095**	Interaction between L1 and Ankyrins	0.01027005 6	ANK2;SPTBN2
**R-HSA-182971**	EGFR downregulation	0.01275969 3	SH3GL3;SH3GL2

**R-HSA-6807878**	COPI-mediated anterograde transport	0.013357171	USO1;ANK2;SPTBN2
**R-HSA-9768919**	NPAS4 regulates expression of target genes	0.015484716	RBFOX3
**R-HSA-8875878**	MET promotes cell motility	0.01843598	LAMA1;LAMA3
**R-HSA-9609736**	Assembly and cell surface presentation of NMDA receptors	0.021604588	NBEA;CASK
**R-HSA-1500931**	Cell-Cell communication	0.02404666	CADM2;LAMA3;CASK
**R-HSA-1489509**	DAG and IP3 signaling	0.024981882	NBEA;ITPR2
**R-HSA-3928665**	EPH-ephrin mediated repulsion of cells	0.026746145	MMP2
**R-HSA-888568**	GABA synthesis	0.027188048	GAD1
**R-HSA-5576891**	Cardiac conduction	0.03044768	ITPR2;ATP1A2
**R-HSA-8939211**	ESR-mediated signaling	0.030796928	MMP7;MMP2
**R-HSA-6794361**	Neurexins and neuroligins	0.031369098	CASK;DLGAP1
**R-HSA-9634815**	Transcriptional Regulation by NPAS4	0.031369098	RBFOX3
**R-HSA-9032759**	NTRK2 activates RAC1	0.031647996	DOCK3
**R-HSA-177929**	Signaling by EGFR	0.032329074	SH3GL3;SH3GL2
**R-HSA-166665**	Terminal pathway of complement	0.036087788	CLU
**R-HSA-112043**	PLC beta mediated events	0.038326293	NBEA;ITPR2
**R-HSA-199977**	ER to Golgi Anterograde Transport	0.040039399	USO1;ANK2;SPTBN2
**R-HSA-2161517**	Abacavir transmembrane transport	0.040507513	ABCB1
**R-HSA-164944**	Nef and signal transduction	0.040507513	ELMO1
**R-HSA-447043**	Neurofascin interactions	0.040507513	CNTN1
**R-HSA-166520**	Signaling by NTRKs	0.041257591	SH3GL3;DOCK3;SH3GL2
**R-HSA-375165**	NCAM signaling for neurite out-growth	0.041472104	NCAM1;SPTBN2
**R-HSA-936837**	Ion transport by P-type ATPases	0.04362168	ATP1A2
**R-HSA-112040**	G-protein mediated events	0.044711834	NBEA;ITPR2
**R-HSA-446107**	Type I hemidesmosome assembly	0.049287121	LAMA3
**R-HSA-9032500**	Activated NTRK2 signals through FYN	0.049287121	DOCK3
**R-HSA-216083**	Integrin cell surface interactions	0.04976213	COL4A6;PECAM1

Supplemental Experimental procedures
HiPSC culture

All the hiPSC lines use for this study are listed in **Table S1.** Wild-type (WT) hiPSC lines were purchased from the HiPSci Human stem cell initiative cell bank (https://www.hipsci.org). $COL4A1^{G755R}$ hiPSC line was generated from skin biopsy from a SVD patient, recruited at the Stroke Research Group at the University of Cambridge (Ethics REC NO 16/EE/0118) and reprogrammed by the Cambridge iPSC core. $COL4A2^{G702D}$ hiPSC line was obtained by Professor Tom Van Agtmael (Murray et al., 2014). Isogenic control lines for $COL4A1^{G755R}$ (iCOL4A1) and $COL4A2^{G702D}$ (iCOL4A2) were generated by CRISPR-gene editing method as described and two independent clones were used for each line (**Figure S1A)**. *COL4A1/A2* mutant and isogenic hiPSC lines were characterized for pluripotency markers expression by immunostaining and quantitative real-time PCR (**Figure S1B,C)** and by formation of the three germ-layers (**Figure S1D).** All hiPSC lines were cultured in TeSR™-E8 media (STEMCELL Technologies) or E8 media (Dulbecco's Modified Eagle Medium/Nutrient Mixture F-12 (DMEM/F-12) with Insulin-Transferrin-Selenium (Thermo Fisher Scientific), Sodium Bicarbonate (Thermo Fisher Scientific), and L-ascorbic acid (Merck) supplemented with FGF2 (4 ug/mL; Biochemistry Department, University of Cambridge) and TGF- β1 (1.74 ug/mL; R&D Systems) using Vitronectin XF (STEMCELL Technologies) as chemically defined xenofree cell culture matrix. All hiPSC lines were validated by the Cambridge Biomedical Research Centre iPSC core and routinely tested for presence of mycoplasma contamination by Mycoplasma Experience LTD.

HiPSC differentiation into mural cells
For Neural Crest (NC) differentiation, hiPSC were detached from Vitronectin coated plates using ReLeSR (STEMCELL Technologies) as previously described (Cheung et al., 2012; Serrano et al., 2019). Clumps were plated at a density of 300 in 0.1% gelatin-coated six well plates in CDM-polyvinyl alcohol (PVA) for 4 days without splitting. CDM was composed of Iscove's modified Dulbecco's medium plus Ham's F12 NUT-MIX (Thermo Fisher Scientific) medium in a 1:1 ratio, supplemented with chemically defined lipid concentrate (Thermo Fisher Scientific), transferrin (Roche Diagnostics), insulin (Roche Diagnostics), and monothioglycerol (Sigma) supplemented with FGF2 (12 ng/mL; R&D Systems) and SB-431542 (10 mmol/L; Tocris), referred as FSB. At day 4, hiPSC was dissociated using TrypLE Express (Thermo Fisher) and seeded as single cells at a 1:3 ratio on 0.1% gelatin-coated plates in FSB. NC cells were passaged every time reached confluence, up to 12 passages.
For mural cells (MC) differentiation, NC cells were dissociated using TrypLE Express and cultured in MC differentiation medium (CDM-PVA supplemented with PDGF-BB (10 ng/ml, Peprotech) and TGF-β1 (2ng/ml, Peprotech) for 12 days (PTD12). For long-term cultures, MC were subsequently grown in MEM (Sigma-Aldrich M5650) containing 10% fetal bovine serum (FBS; Sigma-Aldrich F7524) up to 4 weeks. The majority of the experiments were performed after mural cells were culture in serum-containing media for 2 weeks (2WS).

HiPSC differentiation into BMEC and iECs
hiPSCs were differentiated to brain microvascular endothelial-like cells (BMEC) as previously described, with minor modifications (Hollmann et al., 2017). hiPSCs were washed once with 1X PBS (Corning®), dissociated with StemPro™ Accutase™ Cell Dissociation Reagent (Thermo Fisher Scientific) for 4 minutes, and collected by

centrifugation. hiPSCs were then resuspended in E8 medium containing 10 μM Y27632 (Tocris Bioscience) and seeded onto Matrigel-coated 6-well plates at a density of 1.56×10^4 /cm^2. The following day, the cells were switched to TeSR™-E6 medium (Stem Cell Technologies) to initiate the differentiation. Media was changed every day for 4 days. On day 5, the cells were switched to Endothelial media (EC), which consisted of a basal human endothelial serum-free media (SFM; Thermo Fisher Scientific), supplemented with B27 (Fisher Scientific), basic fibroblast growth factor (bFGF; 10ng/ml; R&D Systems) and all-trans retinoic acid (RA; 10μM; Sigma-Aldrich). Cells were then left to incubate for 48 hours in EC medium without a media exchange. On day 6, resultant BMEC cells were washed with PBS and dissociated with accutase to single cells and plated at a density of 1.1×10^6 /well on 12 well culture plate or 3.3×10^5 /well on 24-well Transwells (CLS3470, Corning®) coated with collagen IV (from human placenta, 1mg/ml, Bornstein, and Traub Type IV; C5533, Sigma Aldrich) and fibronectin (from bovine plasma, 1mg/ml, F1141, Sigma Aldrich). 24 hours after plating, media was refreshed to EC medium without bFGF and RA. Subsequent media changes were performed every 2 days for 6 days.

hiPSC-ECs (iECs) were differentiated using a previously reported protocol with minor modifications (Orlova et al., 2014b). Briefly, hiPSCs were maintained in TeSR™-E8 medium on vitronectin-coated 6-well plates and seeded at day-1. Twenty-four hours after seeding E8 medium was replaced with B(P)EL medium supplemented with 8 μM CHIR. On day 3, the medium was replaced with B(P)EL medium supplemented with VEGF-A (50 ng/ml; Peprotech) and SB431542 (10 μM; Tocris Bioscience) and refreshed on days 6–9. iECs were isolated on day 10 by sorting using MiniMACS separator and CD34 MicroBead kit (Miltenyi Biotec).
iECs from cryo-preserved batches were used in all further experiments.
hiPSC-EC cells were thawed, resuspended in complete Endothelial cell serum-free medium (Gibco), and plated on a 0.1% gelatine-coated culture flask, as previously described. Cells were used for experiments when nearly confluent by visual inspection, typically on day 4. Cells were harvested using TrypLE™ according to the manufacturer's instructions.

HUVEC

HUVEC (Gibco™ C0035C, Thermo Fisher) were plated at a density of 2.5×10^3/cm^2 in culture basal media (Thermo Fisher) in a T75 flask pre-coated overnight with collagen I (0.1% Type 1 collagen from calf skin, MERCK). Medium was changed daily and cells were passaged every 4-7 days using TrypLE express.

CRISPR-mediated gene editing.

To generate the isogenic line for *COL4A1*^{G755R} (iCOL4A1) and *COL4A2*^{G702D} (iCOL4A2), a CRISPR-gene editing method was performed using single guide synthetic RNA (sgRNA; Synthego), SpCas9 protein (Biochemistry Department, University of Cambridge), and a 90-nt single-stranded oligodeoxynucleotide (ssODN; IDT) for homology-directed repair (**Table S2**). To avoid ssODN cleavage by Cas9, a silent mutation was introduced in the NGG codon upstream of the correction site (**Figure S1A**). For gene targeting, 200,000 cells were electroporated with Cas9/sgRNA together with ssODN using the Amaxa 4DNucleofector CA-137 program code (Lonza). Transfected cells were plated onto vitronectin coated-plates in TeSR™-E8 media with 10 μM Y-27632 and CloneR (STEMCELL Technologies). After 48h, the pool of transfected cells was sequenced to test recombination efficiency. Positive

clones were selected by serial dilution and manual selection. Two sub-clones for each isogenic line were used for this study (**Table S1**).

Quantitative real-time polymerase chain reaction.

Complementary DNA (cDNA) was synthesized from 250 ng total RNA using the Maxima First Strand cDNA Synthesis Kit (Thermo Fisher Scientific). Quantitative real-time polymerase chain reaction (PCR) mixtures were prepared with the FAST-SYBR Green Master Mix (Thermo Fisher Scientific) and analyzed using the QuantStudio 7 Flex (Applied Biosystems, Thermo Fisher). Data are expressed as fold change (ΔΔCT) relative to the mean of GAPDH and PBGD housekeeping genes and to the level in hiPSCs of the same cell line.

Primer sequences are listed in **Table S3.**

Immunofluorescence staining and quantification

Adherent cells were fixed using 4% PFA (Boster) for 5 minutes at RT (hiPSC and MC) or 100% ice cold Methanol (BMEC and iECs) for 15 minutes at -20°C and then washed 3 times with 1X PBS containing Calcium and Magnesium (Oxoid). Cells were permeabilized with 0.05% Triton X-100 (Sigma) in PBS and blocked with PBS +3% BSA or 10% FBS for 60 min at RT.

For the detection of collagen IV in the ECM, 250,000 mural cells were plated per wells in a 12-wells plate and after 2 weeks of culture in serum-containing media, ECM was isolated by performing decellularization using 20mM of ammonium hydroxide to lyse the cells. Upon decellularization, matrix was fixed with 4% PFA and then incubated in blocking solution without permeabilization. Primary antibodies (1:200; **Table S4**) incubations were performed at 4°C overnight and Alexa Fluor tagged secondary antibodies (1:400, Molecular Probes Invitrogen) and DAPI (Sigma-Aldrich) applied for 1 hour at room temperature the following day. Images were acquired on a Zeiss LSM 700 confocal and Leica TCS SP5 microscopes and analyzed with Fiji-ImageJ software.

Quantification of fluorescence intensity for collagen IV was performed by taking the mean pixel intensity (Integrated Density, threshold 75-170) from an average of 3-5 fields of view from the same well. For tight junctions quantification in hiPSC-BMEC and iECs, following immunostaining with occludin or claudin-5 antibodies, cells that lacked at least one continuous junction or show one frayed area were classified as discontinuous as previously described (Lee et al., 2018). Images were processed in Fiji-ImageJ software with a minimum of 5 fields with approximately 30 cells/field from three separate differentiations were quantified and all experimental groups remained blinded until completion of the study. All images are representative images.

Western blotting

Cells were lysed in RIPA buffer with added phosphatase inhibitor cocktail (Sigma) and protease inhibitor cocktail (Sigma) on ice for 15min. Protein content was quantified by Pierce Bicinchoninic Acid (BCA) Protein Assay Kit (Thermo Fisher Scientific). Samples (10-20ng) was resolved by electrophoresis on 10-15% Tris-HCl precast sodium dodecyl sulfate (SDS)-polyacrylamide gel (Bio-Rad), then transferred to polyvinylidine difluoride membranes (PVDF; Millipore). Membranes were blocked for 1 h at room temperature with 5% BSA in Tris-Buffered Saline containing 0.1% Tween-20 (TBS-T; Sigma) and incubated overnight with primary antibodies (**Table S4**) at 4°C. Membranes were washed with TBS-T, incubated with horseradish peroxidase (HRP)-conjugated secondary antibodies for 1 h at room temperature and developed with the

Pierce ECL2 western blotting substrate (Thermo Fisher Scientific) using X-ray Developer OPTIMAX (PROTEC GmbH & co) or the Gel Doc™ XR+ system (BioRad). The ImageLab™ Software (v5.2, BioRad) High Resolution programme with Signal Accumulation Mode was used to capture images at incremental exposure times. Anti-β-actin and GAPDH antibody was used as control for equal loading and transfer of the samples. Quantification of bands were performed by Fiji/ImageJ.

Zymography

Gelatin zymography was performed in 10% acrylamide gels containing gelatin (4mg/ml; Novex 10% Zymogram gels; ThermoFisher). SDS-PAGE was performed using Tris-glycine SDS sample and running buffers as described by the manufacturer. After electrophoresis, SDS was replaced by Triton X-100 (2.5%) to renaturate the gelatinases. Gels were incubated in Tris buffer containing NaCl and $ZnCl_2$ at 37°C for 24 h. Gels were then stained with Coomassie Blue solution, followed by de-staining (Methanol, Acetic Acid solution) and visualized by using Gel Doc™ XR+ system (BioRad).

BMEC/iECs Flow Cytometry

Confluent wells were disassociated using Accutase and filtered to a single cell solution through a 40µm cell strainer (Corning®, Fisher Scientific). The cell suspension was fixed using Fixation/Permeabilisation solution (BD Biosciences) at 4ºC for 10 minutes and washed twice in PBS + 10% FBS. Cells were re-suspended with primary antibodies or pre-conjugated antibodies and incubated for 30 minutes at 4ºC. Cells were then resuspended in PBS and measured with a BD LRSFortessa or Canto II Flow Cytometer (BD Bioscience). Flow cytometric data were analyzed with FCSalyzer 0.9.15-alpha software.

Annexin V apoptosis assay. 1×10^6 cells/ml were harvested and resuspended in 1× annexin-binding buffer and incubated with 5 µl of Annexin V–488 (Alexa Fluor 488 Annexin V/Dead Cell Apoptosis Kit; Life technologies) for 15 min at room temperature. Cells were then resuspended in PBS with the addition of propidium iodide (PI 1:300) and measured with a BD LRSFortessa Flow cytometer. Flow cytometric data were analyzed with FCSalyzer 0.9.15-alpha software.

Scratch migration assay

Cells were plated onto 12-well plates and allowed to form a confluent monolayer. The cell monolayer was then scratched in a straight line to make a ‘‘scratch wound’’ with a 1-mL pipette tip. Cells were maintained in DMEM and images of the closure of the scratch were captured at different time points as indicated. Cells were tracked using the Wound_healing_size_tool macro for Fiji/ImageJ.

Mouse aorta dissection and analysis

Animal studies were performed in accordance with UK Home Office regulations (Project license 70/8604). Animals were sacrificed using an increasing gradient of CO_2 according to UK Home Office guidelines, and the thoracic aorta was collected and snap frozen on dry ice. Tissue samples were homogenized using steel beads (Qiagen) in TissueLyser (Qiagen) in RIPA buffer containing protease (Complete Mini, Roche) and phosphatase inhibitors (PhosSTOP, Roche). Protein concentrations were assessed via Pierce BCA Protein Assay (ThermoFisher) and protein were separated

by SDS-PAGE (Mini-Protein Biorad). Membranes were blocked with 5% milk before incubation with primary and secondary antibodies and development using chemiluminescense (Millipore). Protein levels were corrected for Coomassie staining of total protein gels ran or protein stain on membrane (Memcode, Pierce). Densitometry was performed using Image Fiji/ImageJ.

Reference

Cheung, C., Bernardo, A.S., Trotter, M.W.B., Pedersen, R.A., and Sinha, S. (2012). Generation of human vascular smooth muscle subtypes provides insight into embryological origin-dependent disease susceptibility. Nat Biotechnol *30*, 165–173.

Hollmann, E.K., Bailey, A.K., Potharazu, A. v, Neely, M.D., Bowman, A.B., and Lippmann, E.S. (2017). Accelerated differentiation of human induced pluripotent stem cells to blood-brain barrier endothelial cells. Fluids Barriers CNS *14*, 9.

Lee, C.A.A., Seo, H.S., Armien, A.G., Bates, F.S., Tolar, J., and Azarin, S.M. (2018). Modeling and rescue of defective blood-brain barrier function of induced brain microvascular endothelial cells from childhood cerebral adrenoleukodystrophy patients. Fluids Barriers CNS *15,* 9.

Murray, L.S., Lu, Y., Taggart, A., Van Regemorter, N., Vilain, C., Abramowicz, M., Kadler, K.E., and Van Agtmael, T. (2014). Chemical chaperone treatment reduces intracellular accumulation of mutant collagen IV and ameliorates the cellular phenotype of a COL4A2 mutation that causes haemorrhagic stroke. Hum Mol Genet *23*, 283–292.

Orlova, V. v., Drabsch, Y., Freund, C., Petrus-Reurer, S., van den Hil, F.E., Muenthaisong, S., ten Dijke, P., and Mummery, C.L. (2014a). Functionality of endothelial cells and pericytes from human pluripotent stem cells demonstrated in cultured vascular plexus and zebrafish xenografts. Arterioscler Thromb Vasc Biol *34*, 177–186.

Serrano, F., Bernard, W.G., Granata, A., Iyer, D., Steventon, B., Kim, M., Vallier, L., Gambardella, L., and Sinha, S. (2019). A Novel Human Pluripotent Stem Cell-Derived Neural Crest Model of Treacher Collins Syndrome Shows Defects in Cell Death and Migration. Stem Cells Dev *28*.

Shah, S., Kumar, Y., McLean, B., Churchill, A., Stoodley, N., Rankin, J., Rizzu, P., van der Knaap, M., and Jardine, P. (2010). A dominantly inherited mutation in collagen IV A1 (COL4A1) causing childhood onset stroke without porencephaly. Eur J Paediatr Neurol *14*, 182–187. https://doi.org/10.1016/J.EJPN.2009.04.010.
