# Supplementary material for: A novel human iPSC model of COL4A1/A2 small vessel disease unveils a key pathogenic role of matrix metalloproteinases
Source: Stem Cell Reports. 2023 Nov 16;18(12):2386–99. doi: 10.1016/j.stemcr.2023.10.014 (PMC10724071; doi:10.1016/j.stemcr.2023.10.014)
Supplement: Document S2. Article plus supplemental information [file mmc2.pdf]

Stem Cell Reports
Article
OPEN ACCESS

A novel human iPSC model of COL4A1/A2 small vessel disease unveils a key pathogenic role of matrix metalloproteinases

Maha Al-Thani,[1,11] Mary Goodwin-Trotman,[1,11] Steven Bell,[1] Krushangi Patel,[1] Lauren K. Fleming,[2] Catheline Vilain,[3] Marc Abramowicz,[3] Stuart M. Allan,[4] Tao Wang,[5,6] M. Zameel Cader,[7] Karen Horsburgh,[8] Tom Van Agtmael,[2] Sanjay Sinha,[9] Hugh S. Markus,[10] and Alessandra Granata[1,*]

[1]Department of Clinical Neurosciences, Victor Phillip Dahdaleh Heart and Lung Research Institute, University of Cambridge and Royal Papworth Hospital, Cambridge, UK
[2]School of Cardiovascular and Metabolic Health, University of Glasgow, Glasgow, UK
[3]Department of Genetics, Hôpital Erasme, ULB Center of Human Genetics, Universite Libre de Bruxelles, Bruxelles, Belgium
[4]Division of Neuroscience, School of Biological Sciences, Faculty of Biology, Medicine and Health, The University of Manchester, Manchester, UK
[5]Geoffrey Jefferson Brain Research Centre, Manchester Academic Health Science Centre, Northern Care Alliance NHS Foundation Trust, The University of Manchester, Manchester, UK
[6]Division of Evolution, Infection and Genomics, School of Biological Sciences, Faculty of Biology, Medicine and Health, The University of Manchester, Manchester, UK
[7]Nuffield Department of Clinical Neurosciences, Kavli Institute of Nanoscience Discovery, Dorothy Crowfoot Hodgkin Building, Sherrington Road, University of Oxford, Oxford, UK
[8]Centre for Discovery Brain Sciences, University of Edinburgh, Edinburgh, UK
[9]Wellcome-MRC Cambridge Stem Cell Institute, Jeffrey Cheah Biomedical Centre, University of Cambridge, Cambridge, UK
[10]Department of Neurology, Cambridge University Hospitals NHS Foundation Trust, Cambridge, UK
[11]These authors contributed equally
*Correspondence: ag686@cam.ac.uk
https://doi.org/10.1016/j.stemcr.2023.10.014

SUMMARY

Cerebral small vessel disease (SVD) affects the small vessels in the brain and is a leading cause of stroke and dementia. Emerging evidence supports a role of the extracellular matrix (ECM), at the interface between blood and brain, in the progression of SVD pathology, but this remains poorly characterized. To address ECM role in SVD, we developed a co-culture model of mural and endothelial cells using human induced pluripotent stem cells from patients with *COL4A1*/*A2* SVD-related mutations. This model revealed that these mutations induce apoptosis, migration defects, ECM remodeling, and transcriptome changes in mural cells. Importantly, these mural cell defects exert a detrimental effect on endothelial cell tight junctions through paracrine actions. *COL4A1*/*A2* models also express high levels of matrix metalloproteinases (MMPs), and inhibiting MMP activity partially rescues the ECM abnormalities and mural cell phenotypic changes. These data provide a basis for targeting MMP as a therapeutic opportunity in SVD.

INTRODUCTION

Cerebral small vessel disease (SVD) is a leading cause of age-related cognitive decline and contributes to up to 45% of dementia cases worldwide (Gorelick et al., 2011). SVD is also responsible for 20% of ischemic strokes and is a common pathology underlying intracerebral hemorrhage (ICH) (Wardlaw et al., 2019). SVD refers to the sum of all pathological processes that affect the small vessels of the brain, and with an aging population, SVD has major and growing global socio-economic impact (Lam et al., 2022). However, despite its importance, therapeutic approaches for SVD remain limited because of the lack of mechanistic understanding and relevant models required for target identification and drug discovery (Smith and Markus, 2020).

SVD features are associated with advancing age and several vascular risk factors (Wardlaw et al., 2014). Genetic factors have also been reported to be important, with the identification of monogenic forms of SVD (Mancuso et al., 2020) and common variants that increase the risk for sporadic SVD (Chung et al., 2021; Rannikmäe et al., 2015; Traylor et al., 2021). Dominant mutations in collagen type IV, a major component of the microvascular extracellular matrix (ECM), cause SVD presenting with both ICH and ischemia (Gould et al., 2005; Jeanne et al., 2012). *COL4A1* and *COL4A2* mutations cause highly penetrant multi-system disorders by disrupting the ECM homeostasis and leading to ICH and porencephaly in human and mouse models (van Agtmael et al., 2005; Joutel and Faraci, 2014; Murray et al., 2014). Most mutations occur in a glycine (G) residue of the G-X-Y repeat, which characterizes the collagenous domain, and the position of the mutation appeared to correlate with SVD severity (Jeanne et al., 2015). Conversely, variant within the 3′ UTR of *COL4A1* located in a putative miR-29 microRNA binding site results in *COL4A1* upregulation and causes a severe form of ischemic SVD, distinct from the *COL4A1* missense glycine mutation phenotype (Siitonen et al., 2017; Verdura et al., 2016). Patient fibroblasts with *COL4A1* and *COL4A2* gene duplications have also shown increased gene expression, supporting evidence for the pathogenicity of *COL4A1A/2* overexpression in SVD (Kuuluvainen et al., 2021). Importantly, both monogenic

This is an open access article under the CC BY license (http://creativecommons.org/licenses/by/4.0/).

and sporadic forms of *COL4A*-related SVD are likely to share similar pathological mechanisms, as rare coding variants in *COL4A1/A2* also occur in sporadic form of ICH, while common *COL4A1/A2* non-coding variants have been identified as risk factor for sporadic lacunar stroke (Chung et al., 2019; Persyn et al., 2020; Traylor et al., 2021), sporadic ICH (Malik et al., 2018; Rannikmäe et al., 2015), and white matter hyperintensities (Persyn et al., 2020) in the general population. This suggests that insights gained from a model of monogenic *COL4A1/A2* are likely to be relevant to common SVD.

Although the mechanisms leading to SVD are ill defined, there is an emerging focus on the role of the ECM. The ECM of cerebral blood vessels is a key component at the interface between the cerebral microcirculation and the brain, providing structural support to the blood-brain barrier (BBB) as well as influencing cell behavior (Joutel et al., 2016). Genetic studies have revealed that most monogenic forms of SVD are caused by mutations either in genes encoding ECM proteins or in proteins regulating ECM function (Joutel et al., 2016). In addition to this, our recent work has shown that genes related to SVD, including *COL4A1* and *COL4A2*, are significantly enriched in the cerebrovascular ECM network in both mouse and human brain (Pokhilko et al., 2021). To date, the mechanisms by which these ECM defects cause disease remain poorly understood. This underscores the clear need for new models relevant to human SVD.

To provide insights into the pathological mechanisms underlying COL4A1/A2-related SVD, we established a human induced pluripotent stem cell (hiPSC)-based "disease in a dish" model from two individuals with two representative glycine substitutions in the G-X-Y repeat, one in *COL4A1* (G755R) and the other in *COL4A2* (*G702D*) gene (Murray et al., 2014; Shah et al., 2010). We differentiated the hiPSCs into mural cells (MCs) and endothelial cells (ECs) and undertook phenotypic and functional assays and transcriptomic analysis.

RESULTS

Establishment and characterization of COL4A1/A2 hiPSC-derived MCs and ECs

Two hiPSC lines with typical SVD-associated SNPs in *COL4A* genes were used in this study: a *COL4A1*^{G755R} with a G>A substitution in exon 30 of *COL4A1* gene resulting in a change from a glycine to arginine at position 755 from a symptomatic patient and a *COL4A2*^{G702D} with a G>A replacement in exon 28 of *COL4A2* gene, resulting in a change from a glycine to aspartic acid at position 702 from the asymptomatic father of a patient (Table S1) (Murray et al., 2014; Shah et al., 2010). To control for genetic background, we generated isogenic corrected lines, in which the mutant allele (A) in *COL4A1* and *COL4A2* hiPSCs were substituted with the wild-type (WT) allele (G), and two subclones were used for each CRISPRed line (Tables S1 and S2; Figure S1A). As further controls, we used three WT hiPSC lines from healthy individuals (Table S1). hiPSC lines were characterized for pluripotency marker expression by immunostaining, quantitative real-time PCR profiling, and formation of the 3-germ layers (Figures S1B–S1D). hiPSCs were successfully differentiated into MCs of neural crest origin as previously described (Cheung et al., 2012; Serrano et al., 2019) (Figure S2A) and characterized for specific marker expression for neural crest (Figures S2B and S2C) and for MC markers at day 12 of PDGFBB+TGF-β1 differentiation (PTD12) at mRNA levels (Figure S2D) and at the fully differentiated stage at 2 weeks in serum containing media (2WS) using immunohistochemistry and quantitative real-time PCR (Figures 1A, 1B, S2D, and S2E).

MCs express both specific markers for smooth muscle cells (*CNN1*, *ACTA2*, and *TAGLN*) and pericytes (*NG2* and *PDGFRA*), with the disease lines showing significantly increased expression levels for *CNN1* and *ACTA2* at a late stage of differentiation (2WS) (Figures 1B and S2D). MCs are known to produce a variety of ECM proteins, including collagen IV. To assess collagen IV levels in the ECM, both *COL4A1/A2* disease and isogenic hiPSC-derived MCs were plated at equal density, decellularized, and stained with a specific antibody that recognized both collagen IV α1 and α2 chains (Figure 1C). There was a significant reduction in collagen IV staining in the ECM of the disease *COL4A1/A2* mutant lines compared with the controls, as seen in patient fibroblasts (Figure 1D) (Murray et al., 2014). Moreover, hiPSC-derived MCs with *COL4A1*^{G755R} and *COL4A2*^{G702D} have increased migration ability in a scratch assay compared with controls (Figures 1E and 1F). MCs at 2WS also exhibit higher apoptotic levels when stained for annexin V and propidium iodide (PI) using flow cytometry compared with the controls (Figures 1G and 1Hb), similar to previous findings from primary patient fibroblasts and skin biopsy (Murray et al., 2014). Interestingly, no significant changes in apoptotic rates were seen at an earlier stage (PTD12; Figures 1G and 1Ha), at which stage ECM deposition of collagen IV cannot be detected (Figures S2F and S2G). Thus, higher apoptotic rates might be a consequence of increased levels of abnormal collagen IV in the ECM. These data indicate that our hiPSC MCs recapitulate defects of *COL4A1/2* mutations and thus represent a valid model to explore disease mechanisms.

MCs contribute to the barrier phenotype in co-culture and paracrine systems

Brain ECs are known for their barrier function in the BBB, which may be compromised in SVD pathology (Hussain et al., 2021). However, the impact of collagen IV mutations

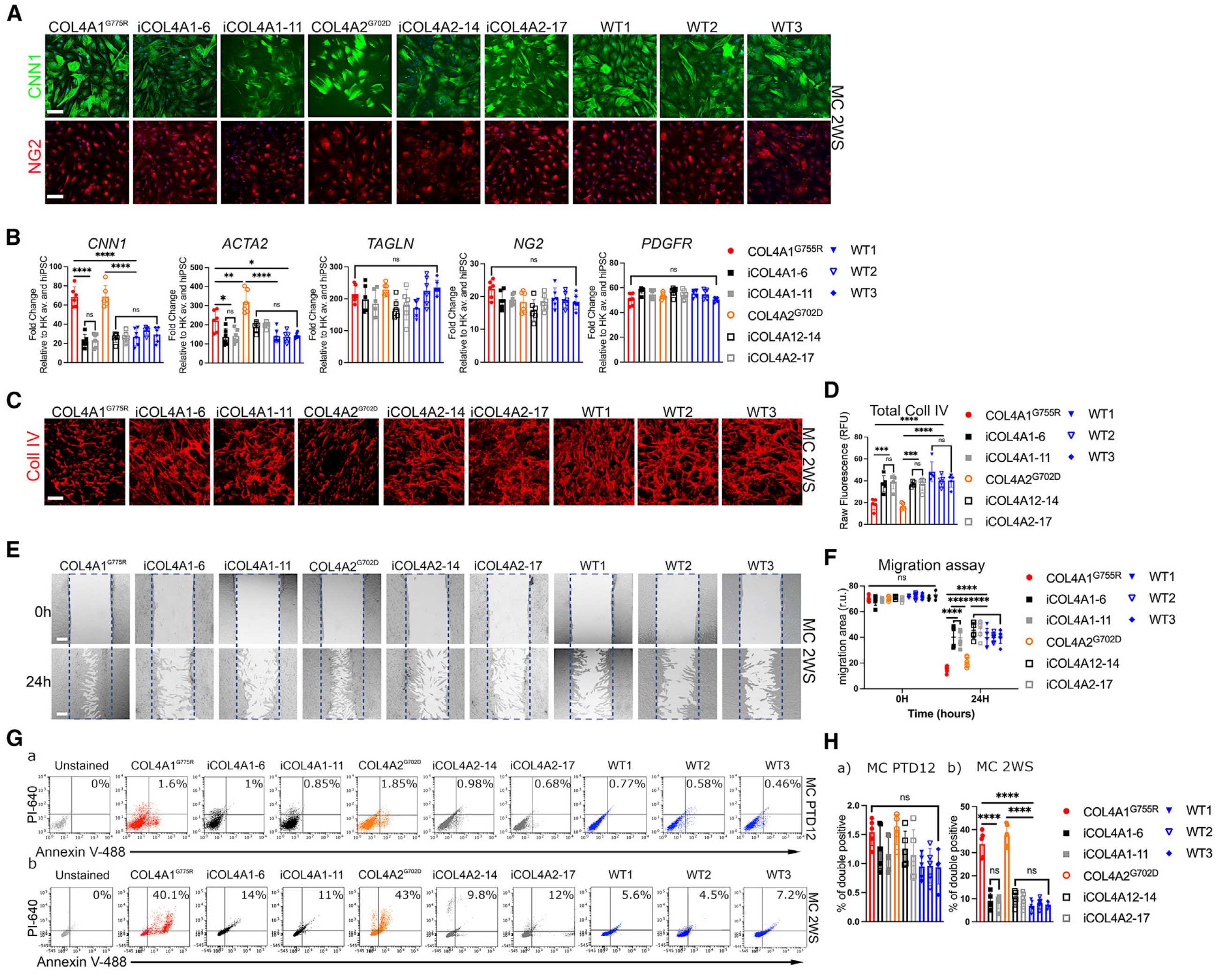

Figure 1. COL4A1^{G755R} and COL4A2^{G702D} hiPSC-derived mural cells (MCs) show abnormal collagen IV and phenotypic changes

(A) Immunostaining for calponin (CNN1) and nerve/glial antigen 2 (NG2) in hiPSC-derived MCs cultured for 2 weeks in serum containing media (2WS) for COL4A1^{G755R}, COL4A2^{G702D}, 2 isogenic subclones for A1 (iCOL4A1-6 and iCOL4A1-11) and A2 (iCOL4A2-14 and iCOL4A2-17), and three healthy controls (WT1, WT2, and WT3; see also Table S1).

(B) quantitative real-time PCR analysis for MC markers, including *CNN1*, *ACTA2*, *TAGLN*, *NG2*, and *PDGFR* (n = 6).

(C) Immunostaining for collagen IV in the ECM of MCs show significant decreased levels in COL4A1^{G755R} and COL4A2^{G702D} when quantified as total fluorescence (D) compared with isogenic and WT controls (n = 6).

(E and F) Representative images of scratch assays for hiPSC MCs (E) and (F) quantification of the areas showing increased migration rate for COL4A1/A2 mutant MC compared with controls (n = 6).

(G) Flow cytometric analysis of annexin V-488 and propidium iodide (PI-640) in hiPSC MCs after 12 days of differentiation in PDGFBB + TGF-β1 (PTD12, early stage; a) and at late stage (2WS; b) show higher apoptotic rate in COL4A1/A2 mutant compared with control MC lines at 2WS (n = 5).

(H) Nuclei were stained with DAPI; scale bar, 100 μm.

Results are presented as mean ± SD of n independent experiments. *p < 0.05, **p < 0.01, ***p < 0.001, and ****p < 0.0001; ns (not significant). Statistical analysis was performed using 2-way ANOVA with Tukey's multiple comparison test.

on the BBB and cross-talk between ECs and MCs remains poorly understood. To assess this, *COL4A1/A2* disease, isogenic, and WT lines were differentiated into brain microvascular endothelial-like cells (BMECs) using a previously established protocol (Figure S3A; (Hollmann et al., 2017)). These BMECs were characterized for expression of specific markers using flow cytometry and quantitative real-time PCR (Figures S3B–S3D). BMECs were then plated

onto a two-dimensional (2D) Transwell setting alone or in presence of MC plated on the basolateral side and maintained for 6 days (Figure 2A). During this time, daily readings were taken of transendothelial electrical resistance (TEER), a robust indicator of EC barrier integrity. TEER measurements expressed as peak values relative to blank (Transwell with no cells) for BMECs alone and in co-culture with MCs were compared (Figure 2B). Isogenic and WT control MCs appear to promote barrier function by significantly increasing TEER values, while disease MCs have little effect on *COL4A1*/*A2* BMEC TEER (Figure 2B). Moreover, MCs were cultured with or without the addition of ascorbic acid in the media to promote collagen synthesis, and similar results were obtained.

Because in this setting, there is no cell-cell interaction between MCs and BMECs, we further assessed the differential MC paracrine effect on the barrier properties by treating the isogenic BMEC clones with conditioned media of *COL4A1*/*A2* MC and vice versa for 6 days (Figure 2C). Interestingly, TEER values of disease BMECs tend to benefit from the isogenic MC paracrine effect (Figure 2D). Conversely, disease MCs exert a paracrine effect by significantly decreasing TEER values in isogenic BMECs (Figure 2E). This MC-mediated paracrine effect on barrier phenotype was confirmed by sodium fluorescein (NaFl) size exclusion paracellular permeability assay (Figure 2F), with the isogenic MCs decreasing NaFl permeability, thus promoting barrier tightness (Figure 2G), while disease MCs appear to increase barrier permeability in isogenic BMECs (Figure 2H). Collectively, these data show the secretome of disease MCs to have detrimental effects on barrier function in *COL4A1*/*A2* SVD models.

COL4A1/A2 MCs affect endothelial tight junction levels and distribution through a paracrine effect

The integrity of tight junctions is essential for the BBB properties of brain ECs (Nitta et al., 2003; Pan et al., 2017). Thus, to assess if tight junctions are affected in *COL4A1*/*A2* hiPSC-derived BMECs, we performed immunostaining analysis for the tight junction proteins occludin and claudin-5 (Figure 3A). We observed striking discontinuities in occludin staining (Figure 3A, white arrow) and frayed junctions evident with claudin-5 staining (Figure 3A, white arrowhead) in *COL4A1*^{G755R} and *COL4A2*^{G702D} BMECs cultured alone. These abnormalities were significantly more frequently in the mutant lines compared with controls (Figure 3B). Moreover, this was associated with reduced occludin and claudin-5 total protein levels (Figures 3C and 3D).

To independently validate these findings and exclude that they were due to the differentiation protocol, we adopted an alternative endothelial differentiation protocol to generate hiPSC-derived ECs (iECs; Figure S3E) (Orlova et al., 2014a, 2014b). These iECs were characterized for expression of specific markers at mRNA levels by quantitative real-time PCR compared with human umbilical vein ECs (HUVECs) as positive control (Figure S3F) and flow cytometry (Figure S3G) and were found to have increased discontinued/frayed junctions as well as lower levels of occludin and claudin-5 proteins in disease lines versus controls (Figures S4A–S4D), validating our findings in BMECs.

To assess if the levels and the distribution of occludin and claudin-5 in ECs is regulated by the MC secretome, *COL4A1*^{G755R} and *COL4A2*^{G702D} hiPSC BMECs were treated with isogenic MC-conditioned media for 4 days prior to immunostaining (Figure 3E). Notably, treatment with isogenic MC media significantly improved the presence of discontinuous and frayed junctions (Figure 3F). Conversely, a greater number of discontinuous and frayed junctions were observed when both isogenic BMEC clones were treated with conditioned media from mutant *COL4A1*/*A2* MC (Figures 3G and 3H). These data clearly support that COL4 SVD includes tight junction defects in ECs that are determined at least in part by a paracrine effect exerted by the MCs.

Transcriptomic analysis highlights ECM abnormalities in COL4A1/A2 MC lines

To identify potential mediators of the MC paracrine effects reported above, we performed a transcriptomic analysis on *COL4A1*^{G755R} and *COL4A2*^{G702D} and corresponding isogenic hiPSC MCs in culture in serum containing media for a week (Figure 4A). From the bulk RNA sequencing (RNA-seq) data, we identified 374 differentially expressed genes (DEGs). No significant difference was observed for *COL4A1* and *COL4A2* mRNA levels between disease and control lines. Importantly, it emerged that 56 DEGs were ECM proteins, and that matrix metalloproteinases (MMPs) were among the proteins misregulated (Figure 4B; Tables S5 and S6). It is known that changes in MMPs levels are associated with barrier disruption and stroke (Candelario-Jalil et al., 2011; Clark et al., 1997; Wallin et al., 2017). To validate the transcriptomics findings, we perform quantitative real-time PCR in early MCs (PTD12) and late MCs (2WS) to profile MMP genes expression (Figures 4C and 4D). We observed a biphasic expression for *MMP2*, which appears to be downregulated at PTD12 and upregulated at the late stage (2WS). *MMP9* mRNA levels were also found to be upregulated in both *COL4A1* and *COL4A2* MCs at the late stage (Figure 4D). *MMP7* shows high expression levels at PTD12 (Figure 4C). In addition, we also found a significant increase in *MMP14* mRNA levels in *COL4A1*/*A2* BMECs (Figure 4E). Interestingly, MMP14, which activates pro-MMP2, was also previously reported to be upregulated in aorta of mice with a *Col4a1* glycine mutation (*Col4a1*$^{+/SVC}$ G1064D) that is a well-established model of Col4a1-associated SVD (Figures S5A and S5B) (van Agtmael et al., 2005; Jones et al., 2016, 2019). The *MMP14* increase was also

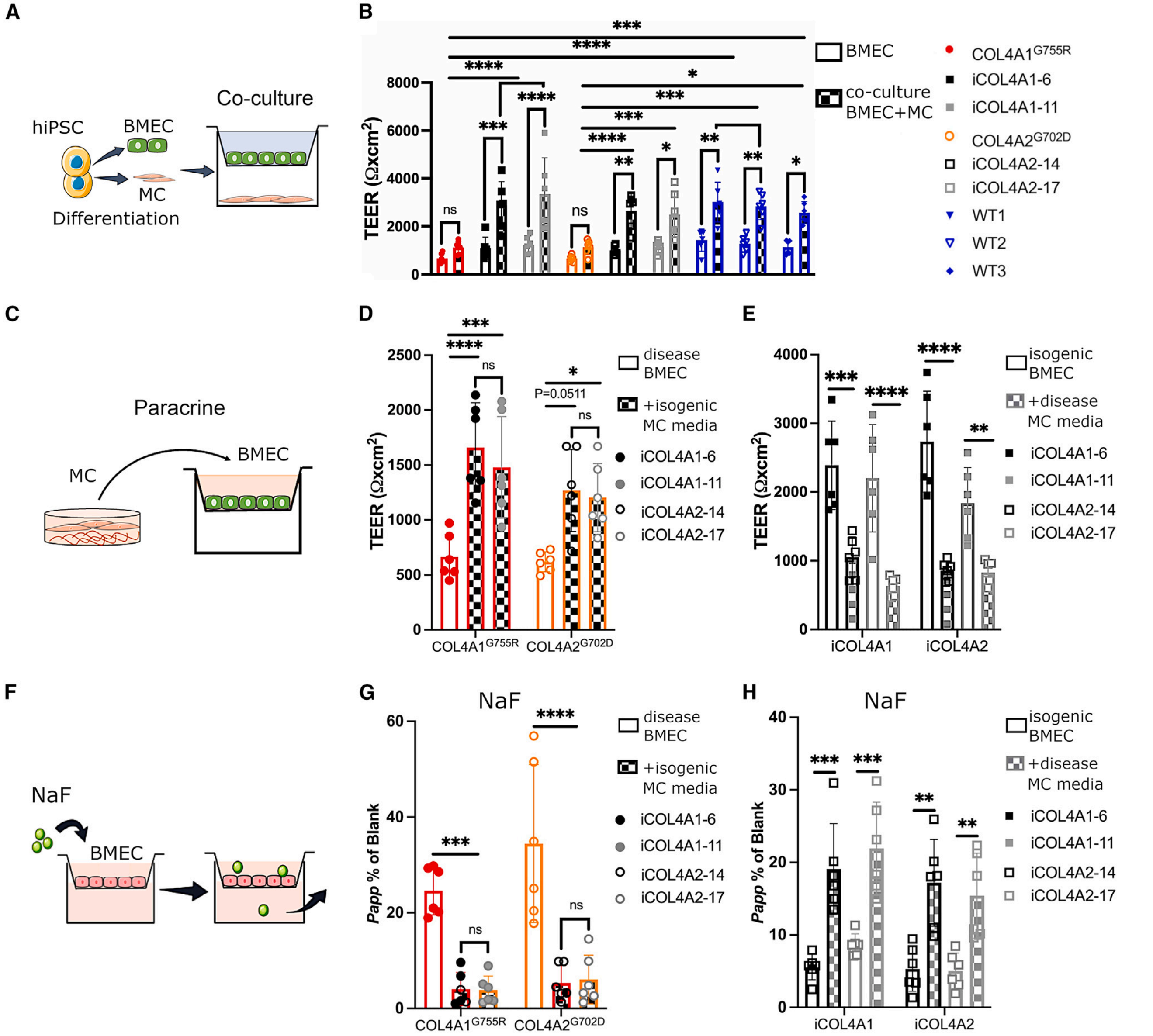

Figure 2. hiPSC-derived mural cells (MCs) contribute to barrier function in a Transwell co-culture system, and COL4A1/A2 lines exert a detrimental effect

(A) Schematic of co-culture with hiPSC-derived MCs and brain microvascular endothelial-like cells (BMECs) in a Transwell device.

(B) Transendothelial electrical resistance (TEER) peak values expressed as resistance (Ω) · cm^2 for hiPSC-derived BMECs in co-culture with MCs increases compared with BMECs alone for isogenic iCOL4A1/A2 and WTs (n = 6).

(C–E) Schematic of the MC paracrine experiment (C), with COL4A1^{G755R} and COL4A2^{G702D} BMEC TEER values benefiting from treatment with isogenic MC-conditioned media (D) (n = 6), while isogenic BMECs show decreased TEER values upon treatment with disease COL4A1/A2 MC-conditioned media (n = 6) (E).

(F–H) Schematic of the sodium fluorescein (NaFl) permeability assay in Transwell setting (F). Isogenic MC paracrine effect positively reduces BMEC permeability in COL4A1^{G755R} and COL4A2^{G702D} lines after 6 days treatment (n = 6) (G), while disease MC-conditioned media-treated isogenic BMECs show increased permeability to NaFl compared with untreated BMECs (n = 6) (H).

TEER, transendothelial electrical resistance; NaFl, sodium fluorescein; *Papp*, apparent permeability. Results are presented as mean ± SD of n independent experiments. *p < 0.05, **p < 0.01, ***p < 0.001, and ****p < 0.0001; ns (not significant). Statistical analysis was performed using 2-way ANOVA with Tukey's multiple-comparison test.

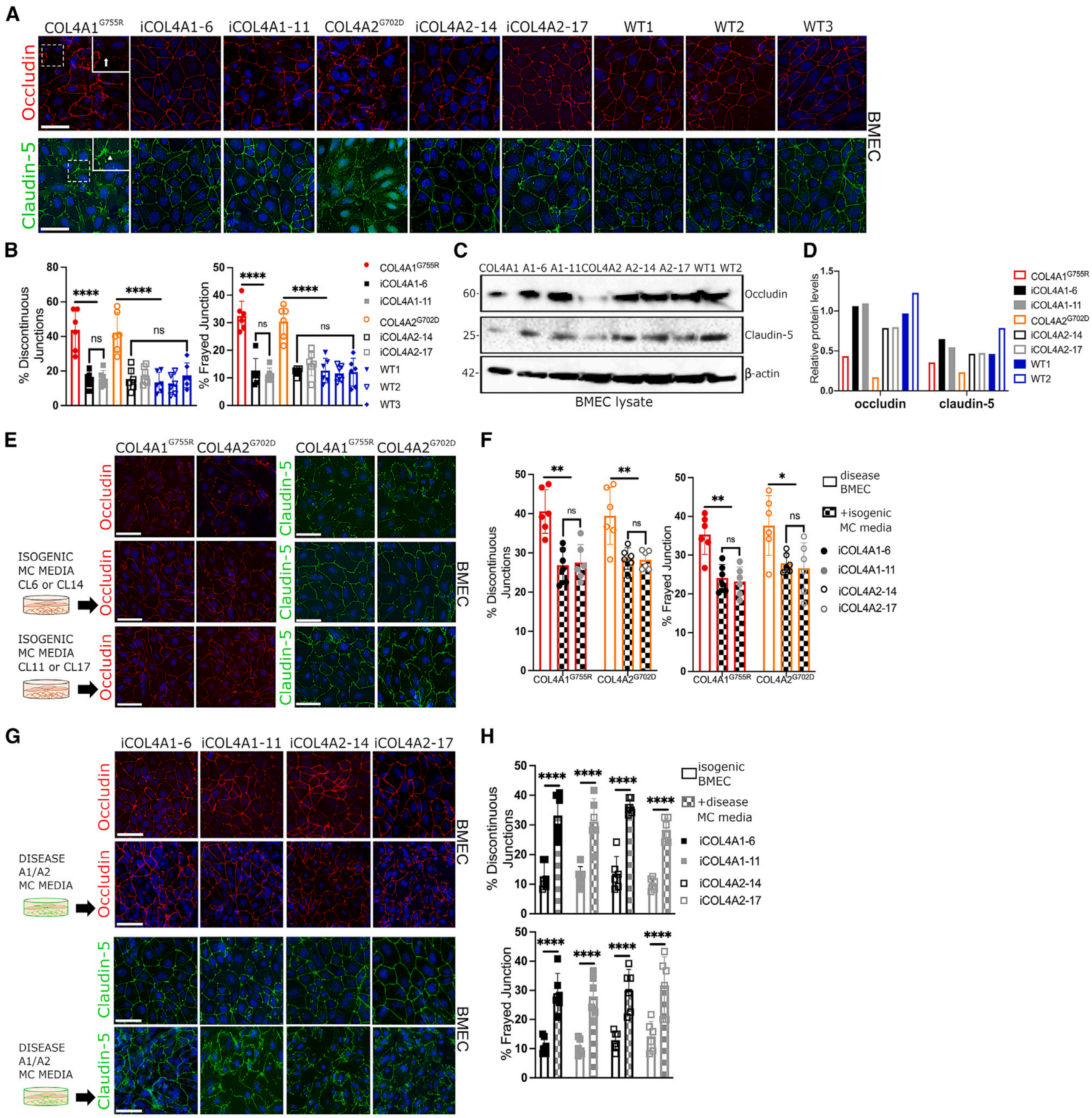

Figure 3. COL4A1/A2 mural cells contribute to endothelial tight junction abnormalities by paracrine effect

(A) Junctional staining for occludin and claudin-5 in hiPSC-derived BMEC lines cultured alone showing discontinuous junction (white arrow) and frayed junction (white arrowed) in zoomed-in insert.

(B) Quantification of discontinuous and frayed junctions show higher percentage in COL4A1^{G755R} and COL4A2^{G702D} lines compared with controls (n = 6).

(C and D) Western blotting analysis and bands quantification show decreased total protein levels for occludin and claudin-5 in COL4A1^{G755R}- and COL4A2^{G702D}-derived BMECs compared with controls (A1 and A2 ISO) and WT1 and WT2. β-Actin was used as loading control (representative blot of n = 3).

(E and F) Immunostaining analysis of COL4A1^{G755R} and COL4A2^{G702D} BMEC tight junctions (occludin and claudin-5) upon 4 days' treatment with isogenic MC-conditioned media show less discontinuous and frayed junctions (n = 6).

(legend continued on next page)

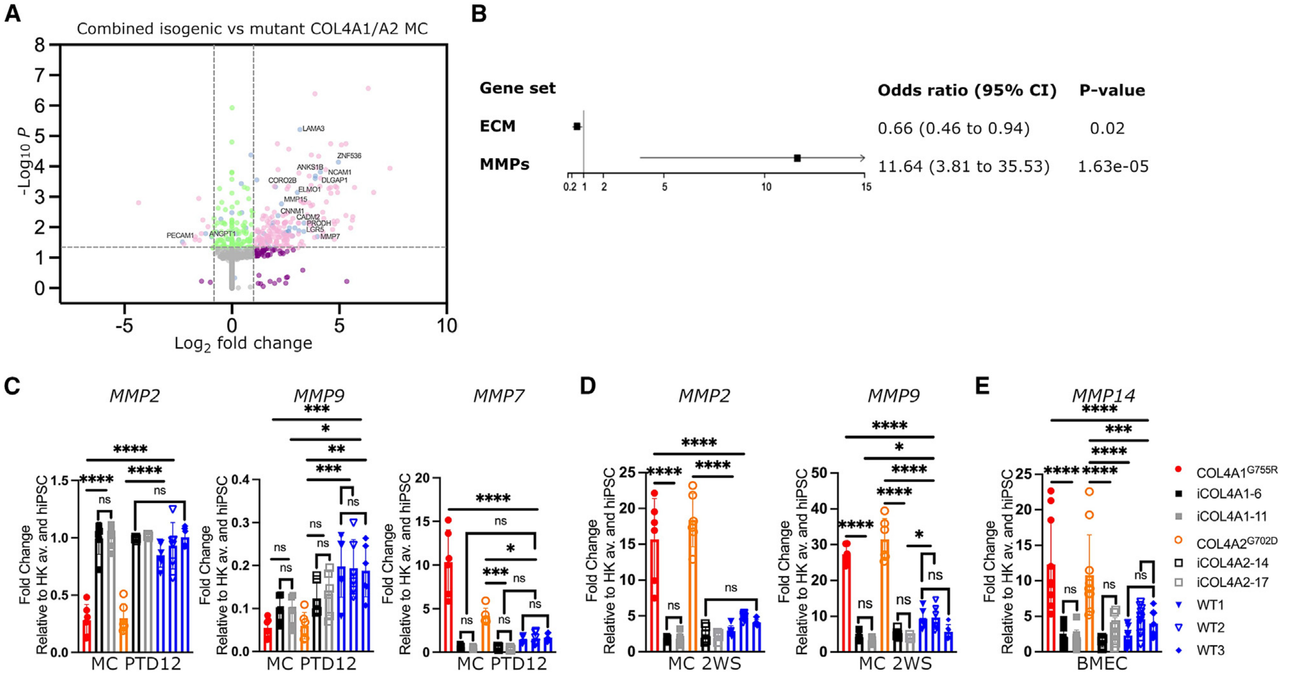

Figure 4. Transcriptomic analysis shows ECM abnormalities in COL4A1/A2 MC lines and MMP upregulation

(A and B) Volcano plot depicting differentially expressed genes in combined COL4A1^{G755R} and COL4A2^{G702D} compared with isogenic MCs (A); matrisome proteins with larger fold changes are labeled, and (B) forest plot shows significant enrichment for ECM and MMPs in diseased MC.

(C and D) quantitative real-time PCR analysis performed at PTD12 and 2WS shows biphasic expression for *MMP2* at mRNA levels in COL4A1^{G755R} and COL4A2^{G702D} MCs and higher levels for MMP7 and MMP9 at PTD12 and 2WS, respectively, in COL4A1^{G755R} and COL4A2^{G702D} MCs compared with the isogenic and WT controls (n = 6).

(E) *MMP14* mRNA was found to be higher in COL4A1/A2 compared with isogenic and WT hiPSC BMEC lines (n = 10).

Results are presented as mean ± SD of n independent experiments. *p < 0.05, **p < 0.01, ***p < 0.001, and ****p < 0.0001; ns (not significant). Statistical analysis was performed using 2-way ANOVA with Tukey's multiple-comparison test.

validated at protein levels in *COL4A1/A2* BMEC and iECs (Figures S5C and S5D). These data clearly support that the ECM and MMPs are dysregulated in *COL4A1/A2* MCs.

MMP inhibition rescues phenotypic alterations, ECM, and tight junction defects

Because MMPs are important for matrix remodeling, including collagens, and because they also target tight junctions for degradation, we hypothesized that MMPs could mediate the *COL4A1/A2* ECM phenotype seen in our *in vitro* model. Thus, we proceeded to treat *COL4A1/A2* hiPSC-derived BMECs with the pan-MMP inhibitor doxycycline (DOXY), which appears to successfully represses MMP2 and MMP9 activity after 72 h treatment by zymography (Figure S5E). However, as DOXY is a broad-spectrum MMP inhibitor with potentially significant side effects, we also tested in our system a small-molecule inhibitor, marimastat (MAR), which specifically targets the MMPs seen dysregulated in our model (including MMP2, MMP9, MMP14, and MMP7). Upon 4 days' treatment with 8 μM DOXY or 1 μM MAR, disease BMECs stained for occludin and claudin-5 show a significant reduction of discontinuous and frayed junction compared with control (DMSO) (Figures 5A and 5B). Similar effects were seen in iECs treated with DOXY or MAR (Figures S5F and S5G).

In addition, treatment with DOXY or MAR appeared to increase total occludin and claudin-5 protein levels by western blotting (Figures 5C and 5D). Remarkably, both DOXY and MAR treatment benefited on BMEC barrier properties, as evidenced by significantly increasing TEER

(G and H) Isogenic BMECs show increased percentage of junction abnormalities upon treatment with disease MC-conditioned media (n = 6). Nuclei were stained with DAPI; scale bar, 100 μm.

Results are presented as mean ± SD of n independent experiments. *p < 0.05, **p < 0.01, ***p < 0.001, and ****p < 0.0001; ns (not significant). Statistical analysis was performed using 2-way ANOVA with Tukey's multiple-comparison test.

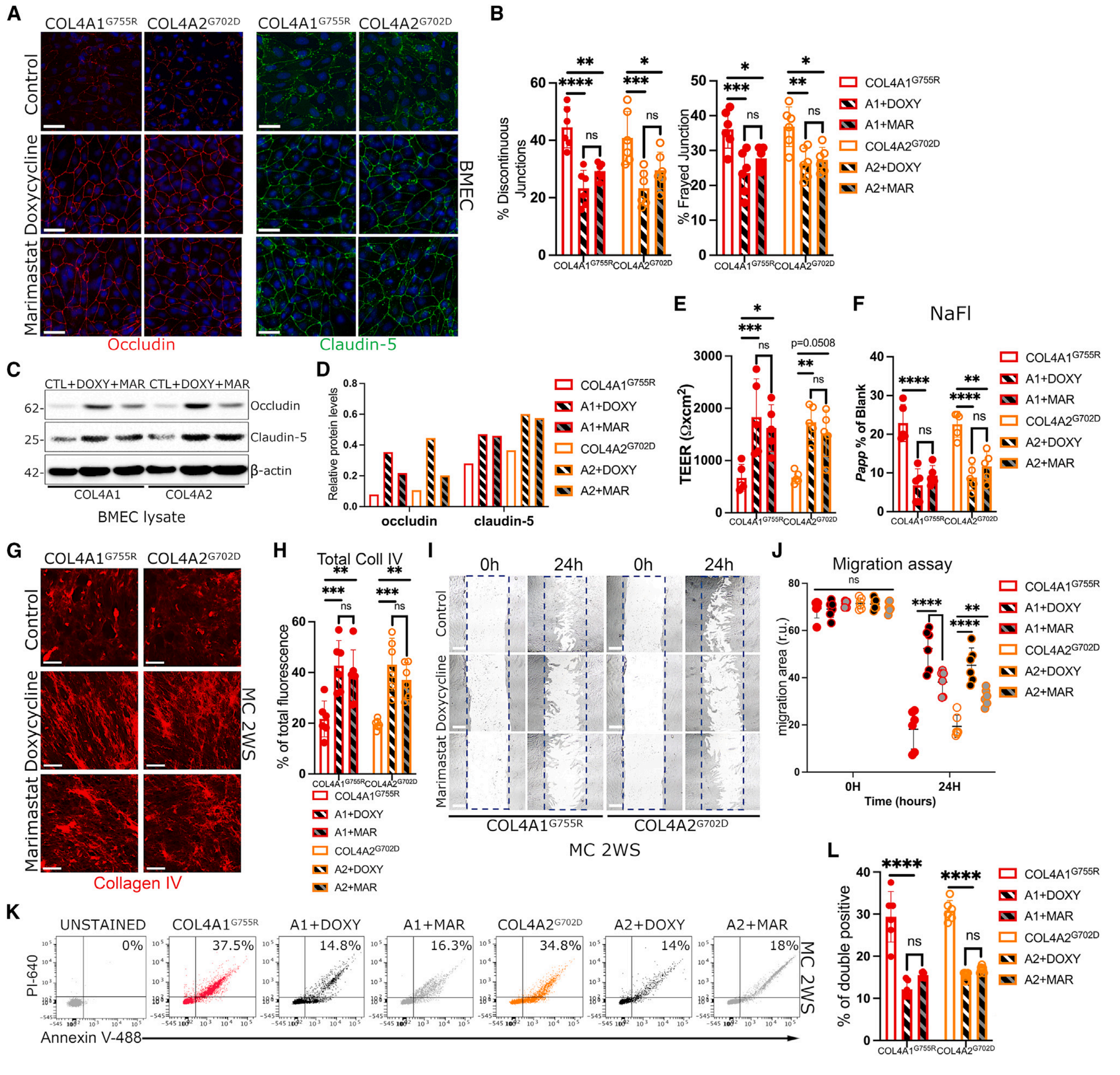

Figure 5. Doxycycline (DOXY) treatment ameliorates tight junction abnormalities and reverts COL4A1/A2 MC collagen IV defect and phenotypic changes

(A and B) Immunostaining analysis quantification of occludin and claudin-5 in COL4A1^{G755R} and COL4A2^{G702D} BMECs treated with DOXY or marimastat (MAR) for 4 days shows lower percentage of tight junction abnormalities (discontinuous and frayed junctions) compared with untreated control (DMSO) (n = 6).

(C and D) Protein blot analysis and band quantification show increased occludin and claudin-5 levels upon treatment with DOXY (+DOXY) and MAR (+MAR) (n = 2). β-Actin was used as loading control.

(E and F) DOXY and MAR treatments improve both TEER (E) and NaFl (F) readouts in *COL4A1/A2* mutant BMECs compared with untreated controls (n = 6).

(G) Immunostaining analysis of collagen IV in the decellularized ECM of *COL4A1^{G755R}* and *COL4A2^{G702D}* MCs at late stage (2WS) upon 4 days treatment with DOXY (10 μM) or MAR (1 μM), and total fluorescence quantification (H) show higher fluorescence in ECM compared with control (n = 6).

(I and J) Representative image of scratch assay for *COL4A1/A2* hiPSC-derived MC control (DMSO), DOXY treated and MAR treated at 0 and 24 h (I), and scratch area quantification (J) show lower migration rate upon treatment with DOXY or MAR compared with controls (n = 6).

(legend continued on next page)

values (Figure 5E) and reducing the NaFl permeability percentage (Figure 5F).

We also looked at the effect of inhibiting MMPs by DOXY and MAR treatments on collagen IV deposition in *COL4A1/A2* hiPSC-derived MCs, and we found that collagen IV fluorescence levels detected by immunostaining in the *COL4A1/A2* decellularized ECM increased upon treatments compared with controls (Figures 5G and 5H). DOXY- and MAR-treated disease MCs also show a significant decreased migration rate at 24 h (Figures 5I and 5J) and lower apoptotic levels (Figures 5K and 5L) comparable with controls (Figure 1G). These data establish a role for ECM remodeling due to MMPs caused by *COL4A1/A2* mutations and provide *in vitro* evidence that modulating specific MMPs may represent therapeutic targets for SVD.

DISCUSSION

There is a critical need to develop new models relevant to human SVD to provide mechanistic insights as well as a foundation to test potential treatments for this debilitating disorder. To address this, we characterized a novel *in vitro* model of human SVD produced by differentiating iPSCs generated from patients with *COL4A1*- or *COL4A2* SVD-related mutations into MCs.

We used *COL4A1/A2* patient-derived hiPSC MCs in a co-culture system with BMECs to mimic the changes seen in patients' small vessels and to investigate underlying pathological mechanisms. First, we observed increased expression of smooth muscle cells markers, such as *CNN1* and *ACTA2*, in *COL4A1/A2* MCs at late stage of differentiation, which may suggest hypermuscularization, as previously shown in a Col4a1 mouse model (Ratelade et al., 2020). Disease MCs also showed an ECM defect, including lower levels of extracellular collagen IV, in agreement with previous findings from patient cells, indicating that SNPs in the triple-helix-forming domain are likely to affect the protein conformation, which in turn may destabilize collagen IV deposition in the ECM (Jeanne et al., 2015; Murray et al., 2014). We also determined phenotypic changes in disease MCs, including increased migration and apoptotic rates that parallel previous studies using primary patient fibroblasts (Murray et al., 2014). A loss of MCs has been reported before and could be caused by several mechanisms, including ECM remodeling and endoplasmic reticular stress (Ratelade et al., 2018, 2020).

Given the key strategic location of the ECM at the interface between blood and brain, a central aim of the study was to determine where COL4 disease influences barrier-related properties. Interestingly, *COL4A1/A2* patient-derived MCs exerted a detrimental effect on the endothelial barrier functions by a paracrine effect, evidenced by our Transwell setup and with MC-conditioned media treatment.

In this study, for the first time, we provided insight into the transcriptional features of *COL4A1/A2* patient-derived MCs. Strikingly 15% of changes affected ECM proteins, including MMPs. Collagen IV is a substrate for the proteolytic activity of the gelatinases MMP2 and MMP9 and the matrilysin MMP7. Increased MMP2 and MMP9 expression has been associated with breakdown of collagen type IV in both human and animal models (Roach et al., 2002; Rosell et al., 2008), as well as with degradation and cellular rearrangement of the endothelial tight junctions (Bauer et al., 2010; Liu et al., 2012; Yang et al., 2007). Recently, MMP7 has been found to correlate with BBB dysfunction following traumatic brain injury (Nichols et al., 2021). Moreover, MMPs are known to play a role in smooth muscle migratory behavior and may facilitate MC migration in our *COL4A1/A2* model by promoting ECM proteins proteolysis (Underly et al., 2017). Interestingly, we observed a biphasic change for *MMP2* mRNA with expression levels increasing at later differentiation stage, which corresponds with greater ECM deposition. This suggests that abnormal collagen IV deposition may contribute to higher MMP activity, which in turn could lead to increased cell death seen in our disease models.

These findings suggest MMPs could play a role in the ECM alterations in *COL4A1/A2*-related SVD and could present a novel therapeutic opportunity. In support of this, targeting MMPs using the pan-MMP inhibitor DOXY partially rescued the disease MC phenotypes, including promoting collagen IV extracellular levels, reducing migration and apoptotic levels, and improving BMEC/iEC tight junction abnormalities. In other studies, DOXY was shown to reduced vascular remodeling and damage induced by cerebral ischemia in a stroke animal model, the stroke-prone spontaneously hypertensive rats (Pires et al., 2011). However, DOXY is a broad-spectrum MMP inhibitor with potentially significant side effects. For this reason, we tested the small-molecule inhibitor MAR, which specifically targets the MMPs seen dysregulated in our models. MAR was the first MMP inhibitor to be tested in clinical trials and is now used for patients with different types of

(K and L) DOXY (+DOXY) and MAR (+MAR) treatments improve apoptotic levels in *COL4A1*^{G755R} and *COL4A2*^{G702D} MCs at 2WS compared with untreated (n = 5).

Nuclei were stained with DAPI; scale bar, 100 μm.

Results are presented as mean ± SD of n independent experiments. *p < 0.05, **p < 0.01, ***p < 0.001, and ****p < 0.0001; ns (not significant). Statistical analysis was performed using 2-way ANOVA with Tukey's multiple-comparison test.

cancer (Thomas and Steward, 2005). Importantly, it was well tolerated by patients with short-term treatment.

Overall, major strengths of this work are as follows: (1) we generated a new hiPSC-derived disease model for SVD, which replicates phenotypic changes observed in patients and *Col4a1* animal model, including ECM abnormalities, and (2) this disease-relevant model can be used as new tool for the analysis of signaling pathways to identify therapeutic targets, such as specific MMP, and (3) to screen and test for potential drugs against SVD.

Our work has limitations. First, the *COL4A2* hiPSC line was generated from the asymptomatic father of the patient. Previously, it has been shown that the father's fibroblasts lack some of the properties seen in the patient's fibroblasts. However, in our model, *COL4A2* phenotypic changes are comparable with the *COL4A1* line (symptomatic), and this may be due to the use of relevant cell types to investigate these changes.

Second, generating representative brain ECs that possess endothelial identity while replicating the BBB properties, including elevated TEER and small-molecule low permeability, has been a challenge highlighted in recent hiPSC work (Lu et al., 2021). We initially used the protocol of Hollman et al., which originated from the Lippman lab (Hollmann et al., 2017). These cells display high TEER, but they do also express epithelium-related genes and lack angiogenic properties (Lu et al., 2021). In view of these limitations, we then successfully validated our results using a generic endothelial protocol (Orlova et al., 2014b), but this lacks barrier-like functions. Further research is required to improve the current protocols for generation of BMECs, on the basis of the emerging understanding of the BBB from single-cell sequencing studies (Garcia et al., 2022).

In conclusion, our novel hiPSC-derived MC model of *COL4A1*/*A2* mutations supports a key role of the ECM in SVD and suggests that targeting ECM-related proteins such as MMPs may be a promising potential therapeutic option.

EXPERIMENTAL PROCEDURES

Resource availability

Corresponding author

Further information and requests for resources and reagents should be directed to and will be fulfilled by the corresponding author, Alessandra Granata (ag686@cam.ac.uk).

Materials availability

This study did not generate new unique reagents. Materials are listed in supplemental experimental procedures in the supplemental information and can be requested from the corresponding author.

Data and code availability

The RNA-seq analysis data generated during this study have been deposited on Apollo - University of Cambridge Repository: https://doi.org/10.17863/CAM.100127 and is publicly available.

Experimental methods

HiPSC culture

All the hiPSC lines use for this study are listed in Table S1. Full culture condition and medium formulation can be found in the supplemental information.

HiPSC differentiation into MCs

hiPSCs were differentiated into MCs of neural crest origin using a previously described protocol (Cheung et al., 2012; Serrano et al., 2019). Full culture condition and medium formulation can be found in the supplemental information.

HiPSC differentiation into BMECs and iECs

hiPSCs were differentiated to BMECs as previously described, with minor modifications (Hollmann et al., 2017). iECs were differentiated using a previously reported protocol with minor modifications (Orlova et al., 2014b). Full culture conditions and medium formulation can be found in the supplemental information.

Transwell co-culture

Either 12- or 24-well Transwells (Corning 0.4 μm pore; Sigma-Aldrich) were coated on the apical and basolateral side with collagen IV/fibronectin. hiPSC MCs were dissociated with TrypLE and seeded onto the plate bottom of the Transwell coated with 0.1% gelatin. After incubation for 1 h, hiPSC BMECs were dissociated and the seeded onto the apical side. The next day, Transwells with BMECs with(out) MCs were maintained without any further medium changes for up to 6 days before analyses.

Paracrine. MCs were serum starved for 4 days. At day 5, the MC serum-starved conditioned media was added to BMEC seeded onto collagen IV/fibronectin coated 24-well Transwells for TEER and NaFl analyses or 24-well plates for immunostaining assay. The initial TEER measurement was taken after 24 h and afterward on a daily basis. For NaFl and immunostaining assays, BMECs were treated with condition media, refreshed every other day, for 6 days.

BMEC functional assays

TEER. TEER measurements were taken every 24 h, from day 1 to day 6 of subculture of BMECs onto Transwells using an EVOM2 Voltohmmeter/STX2 electrodes (World Precision Instruments). The STX2 electrode was positioned within the well and the resistance (Ω) was recorded three times to calculate the mean resistance. All values are given as $\Omega \cdot cm^2$ after subtracting the resistance of an empty coated Transwell maintained in the same culture media (blank) and multiplying by the surface area ($0.33\ cm^2$), as described previously (Lee et al., 2018). TEER was expressed as peak value.

NaFl. At 2 days post-subculture of BMECs onto 24-well Transwells, spent medium was removed from the upper chamber of the Transwell and replaced with 600 μL NaFl (1 mg/mL; Sigma-Aldrich) diluted 1:100 in endothelial serum-free media with B27. Samples of 100 μL were taken from the basolateral side every two hours for eight hours. Raw fluorescence was measured with a TECAN Infinite M200 Pro plate reader (excitation wavelength 460 nm and emission wavelength 515 nm; gain of 50, 25 flashes; z-position 20,000). Quantification was represented as percentage of total fluorescence relative to empty coated Transwell (blank), as previously described (Lee et al., 2018).

DOXY and MAR treatments

hiPSC-derived MCs were treated with DOXY (10 μM; Sigma-Aldrich) or MAR (AstraZeneca; 1 μM in DMSO) in DMEM + 10% fetal bovine serum (FBS) for 4 days, with media change every other

day, and then harvested for analyses. BMECs/iECs were treated with 8 μM DOXY or MAR (1 μM) in EC medium, with media change every other day, and collected at 24 and 72 h for zymography and at day 6 for immunostaining and western blotting analysis. TEER measurements were taken every 24 h from day 1 to day 6 of subculture of BMECs onto Transwells in media supplemented with DOXY or MAR. NaFl permeability assay was performed after 6 days of DOXY or MAR treatment.

RNA-seq

Sample preparation. Three sets (biological replicates) of hiPSC-derived MCs grown in DMEM + 10% serum for 1 week were harvested. Total RNA was isolated from cells using the RNeasy Mini Kit (QIAGEN). Upon ribosomal RNA depletion, libraries were prepared using a NEBNext RNA library Prep kit (Illumina). The samples were run on a Novaseq6000 S4 lane, and 150 bp paired-end reads were generated.

Data analysis. The resulting base call files were converted to fastq files using the bcl2fastq program. Alignment in STAR (version 2.7.10a) using a modified version of the ENCODE-DCC RNA-seq pipeline annotated using GENCODE version 39 (hg38) was performed (Dobin et al., 2013). Gene-level RNA expression quantification was performed using RSEM (Li et al., 2011).

Differential expression analyses were carried out using DESeq2 in R version 4.0.4 (Love et al., 2014). We specified a false discovery rate of 5% and applied a Bayesian shrinkage estimator to effect sizes using approximation of the posterior for individual coefficients. Results were visualized using the EnhancedVolcano package.

Enrichment of gene sets of interest was calculated using logistic regression. We used data from human samples to categorize genes associated with the ECM (Pokhilko et al., 2021) and MMPs (Table S5). Pathways enrichment analysis was performed using the Reactome (Table S6) (Wu and Haw, 2017). We chose a 5% false discovery rate (FDR) to indicate statistical significance.

Statistical analysis

Data, expressed as mean ± SD, were analyzed statistically using SPSS version 22.0. Unpaired Student's t test for two-group comparisons or one-way ANOVA followed by least significantly different (LSD) multiple comparisons was performed using Prism version 9.00 (GraphPad Software, Inc.) to analyze the significant difference, which was indicated as ns (not significant; $p > 0.05$), *$p < 0.05$, **$p < 0.01$, ***$p < 0.001$, and ****$p < 0.0001$. The n noted in the figure legends represents the replicated number of biological experiments. All data are representative of at least three independent experiments.

SUPPLEMENTAL INFORMATION

Supplemental information can be found online at https://doi.org/10.1016/j.stemcr.2023.10.014.

ACKNOWLEDGMENTS

We thank the National Institute for Health and Care Research (NIHR) Cambridge Biomedical Research Centre (BRC) Cell Phenotyping Hub and the Flow Cytometry Core facilities at the Cambridge Institute for Medical Research (CIMR). We thank Dr. David Smith, principal scientist at AstraZeneca R&D, for providing MAR through the Open Innovation platform. We also thank Professor L. Vallier and the hiPSC core facility for generating the *COL4A1* hiPSC line. The present work was supported by a Stroke Association priority program award in Advancing Care and Treatment of Vascular Dementia (grant 16VAD_04) in partnership with the British Heart Foundation and Alzheimer's Society to K.H., S.A., T.V.A., A.G., H.S.M., S.S., M.Z.C., and T.W. A.G. is supported by the Medical Research Foundation mid-career fellowship (RG98759). This research was funded by the British Heart Foundation via the Cambridge British Heart Foundation Centre of Research Excellence (RE/18/1/34212) and a British Heart Foundation program grant (RG/F/22/110052). Infrastructural support was provided by the Cambridge University Hospitals NIHR BRC (BRC-1215-20014). M.Z.C. is supported by the Innovative Medicine Initiative 2 Joint Undertaking (grant agreement No 807015). This joint undertaking receives support from the European Union's Horizon 2020 research and innovation program and the European Federation of Pharmaceutical Industries and Associations (EFPIA). S.S. is supported by a BHF Senior Clinical Fellowship (FS/18/46/33663). H.S.M. is supported by a NIHR Senior Investigator Award. The views expressed are those of the authors and not necessarily those of the NIHR or the Department of Health and Social Care.

AUTHOR CONTRIBUTIONS

A.G. contributed to the conception, design, and interpretation of the hiPSC model data and to the drafting of the article. M.A. and M.G.-T. equally contributed to the acquisition and analysis of the data. S.B. undertook the transcriptomic data analysis. K.P. helped with hiPSC differentiation and establishing the co-culture and paracrine model. T.V.A. and L.K.F. undertook the mouse aorta dissection and analysis. K.H., H.S.M. and T.V.A. provided critical reading of the manuscript. C.V. and M.A. are the clinicians for the patient with the *COL4A2* mutation. H.S.M. is the clinician for the patient with *COL4A1* mutation and contributed to the revision of the article and the supervision of all studies. All authors contributed to the article and approved the submitted version.

DECLARATION OF INTERESTS

The authors declare no competing interests.

Received: February 17, 2023
Revised: October 19, 2023
Accepted: October 20, 2023
Published: November 16, 2023

REFERENCES

van Agtmael, T., Schlötzer-Schrehardt, U., McKie, L., Brownstein, D.G., Lee, A.W., Cross, S.H., Sado, Y., Mullins, J.J., Pöschl, E., and Jackson, I.J. (2005). Dominant mutations of Col4a1 result in basement membrane defects which lead to anterior segment dysgenesis and glomerulopathy. Hum. Mol. Genet. *14*, 3161–3168.

Bauer, A.T., Bürgers, H.F., Rabie, T., and Marti, H.H. (2010). Matrix metalloproteinase-9 mediates hypoxia-induced vascular leakage in the brain via tight junction rearrangement. J. Cerebr. Blood Flow Metabol. *30*, 837–848.

Candelario-Jalil, E., Thompson, J., Taheri, S., Grossetete, M., Adair, J.C., Edmonds, E., Prestopnik, J., Wills, J., and Rosenberg, G.A. (2011). Matrix metalloproteinases are associated with increased blood-brain barrier opening in vascular cognitive impairment. Stroke *42*, 1345–1350.

Cheung, C., Bernardo, A.S., Trotter, M.W.B., Pedersen, R.A., and Sinha, S. (2012). Generation of human vascular smooth muscle subtypes provides insight into embryological origin-dependent disease susceptibility. Nat. Biotechnol. *30*, 165–173.

Chung, J., Brown, D.L., Pera, J., Marini, S., Jimenez-Conde, J., Norrving, B., Fernandez-Cadenas, I., Roquer, J., Selim, M., Tirschwell, D.L., et al. (2019). Genome-wide association study of cerebral small vessel disease reveals established and novel loci. Brain *142*, 3176–3189.

Chung, J., Marini, S., Pera, J., Norrving, B., Jimenez-Conde, J., Roquer, J., Fernandez-Cadenas, I., Tirschwell, D.L., Selim, M., Brown, D.L., et al. (2021). Genome-wide association study of cerebral small vessel disease reveals established and novel loci on behalf of the International Stroke Genetics Consortium. Brain *142*, 3176–3189. https://doi.org/10.1093/brain/awz233.

Clark, A.W., Krekoski, C.A., Bou, S.S., Chapman, K.R., and Edwards, D.R. (1997). Increased gelatinase A (MMP-2) and gelatinase B (MMP-9) activities in human brain after focal ischemia. Neurosci. Lett. *238*, 53–56.

Dobin, A., Davis, C.A., Schlesinger, F., Drenkow, J., Zaleski, C., Jha, S., Batut, P., Chaisson, M., and Gingeras, T.R. (2013). STAR: ultrafast universal RNA-seq aligner. Bioinformatics *29*, 15–21.

Garcia, F.J., Sun, N., Lee, H., Godlewski, B., Mathys, H., Galani, K., Zhou, B., Jiang, X., Ng, A.P., Mantero, J., et al. (2022). Single-cell dissection of the human brain vasculature. Nature *603*, 893–899.

Gorelick, P.B., Scuteri, A., Black, S.E., Decarli, C., Greenberg, S.M., Iadecola, C., Launer, L.J., Laurent, S., Lopez, O.L., Nyenhuis, D., et al. (2011). Vascular contributions to cognitive impairment and dementia: a statement for healthcare professionals from the american heart association/american stroke association. Stroke *42*, 2672–2713.

Gould, D.B., Phalan, F.C., Breedveld, G.J., van Mil, S.E., Smith, R.S., Schimenti, J.C., Aguglia, U., van der Knaap, M.S., Heutink, P., and John, S.W.M. (2005). Mutations in Col4a1 cause perinatal cerebral hemorrhage and porencephaly. Science *308*, 1167–1171.

Hollmann, E.K., Bailey, A.K., Potharazu, A. v, Neely, M.D., Bowman, A.B., and Lippmann, E.S. (2017). Accelerated differentiation of human induced pluripotent stem cells to blood-brain barrier endothelial cells. Fluids Barriers CNS *14*, 9.

Hussain, B., Fang, C., and Chang, J. (2021). Blood-Brain Barrier Breakdown: An Emerging Biomarker of Cognitive Impairment in Normal Aging and Dementia. Front. Neurosci. *15*, 688090.

Jeanne, M., Labelle-Dumais, C., Jorgensen, J., Kauffman, W.B., Mancini, G.M., Favor, J., Valant, V., Greenberg, S.M., Rosand, J., and Gould, D.B (2012). COL4A2 mutations impair COL4A1 and COL4A2 secretion and cause hemorrhagic stroke. Am. J. Hum. Genet. *90*, 91–101.

Jeanne, M., Jorgensen, J., and Gould, D.B. (2015). Molecular and Genetic Analyses of Collagen Type IV Mutant Mouse Models of Spontaneous Intracerebral Hemorrhage Identify Mechanisms for Stroke Prevention. Circulation *131*, 1555–1565.

Jones, F.E., Bailey, M.A., Murray, L.S., Lu, Y., McNeilly, S., Schlötzer-Schrehardt, U., Lennon, R., Sado, Y., Brownstein, D.G., Mullins, J.J., et al. (2016). ER stress and basement membrane defects combine to cause glomerular and tubular renal disease resulting from Col4a1 mutations in mice. Dis. Model. Mech. *9*, 165–176.

Jones, F.E., Murray, L.S., McNeilly, S., Dean, A., Aman, A., Lu, Y., Nikolova, N., Malomgré, R., Horsburgh, K., Holmes, W.M., et al. (2019). 4-Sodium phenyl butyric acid has both efficacy and counter-indicative effects in the treatment of Col4a1 disease. Hum. Mol. Genet. *28*, 628–638.

Joutel, A., and Faraci, F.M. (2014). Cerebral small vessel disease: insights and opportunities from mouse models of collagen IV-related small vessel disease and cerebral autosomal dominant arteriopathy with subcortical infarcts and leukoencephalopathy. Stroke *45*, 1215–1221.

Joutel, A., Haddad, I., Ratelade, J., and Nelson, M.T. (2016). Perturbations of the cerebrovascular matrisome: A convergent mechanism in small vessel disease of the brain? J. Cerebr. Blood Flow Metabol. *36*, 143–157.

Kuuluvainen, L., Mönkäre, S., Kokkonen, H., Zhao, F., Verkkoniemi-Ahola, A., Schleutker, J., Hakonen, A.H., Hartikainen, P., Pöyhönen, M., and Myllykangas, L. (2021). COL4A1 and COL4A2 Duplication Causes Cerebral Small Vessel Disease with Recurrent Early Onset Ischemic Strokes. Stroke *52*, E624–E625.

Lam, B.Y.K., Cai, Y., Akinyemi, R., Biessels, G.J., van den Brink, H., Chen, C., Cheung, C.W., Chow, K.N., Chung, H.K.H., Duering, M., et al. (2022). The global burden of cerebral small vessel disease in low- and middle-income countries: A systematic review and meta-analysis. Int. J. Stroke *18*, 15–27.

Lee, C.A.A., Seo, H.S., Armien, A.G., Bates, F.S., Tolar, J., and Azarin, S.M. (2018). Modeling and rescue of defective blood-brain barrier function of induced brain microvascular endothelial cells from childhood cerebral adrenoleukodystrophy patients. Fluids Barriers CNS *15*, 9.

Li, B.N., Chui, C.K., Chang, S., and Ong, S.H. (2011). RSEM: Accurate transcript quantification from RNA-Seq data with or without a reference genome. Comput. Biol. Med. *41*, 1–10.

Liu, J., Jin, X., Liu, K.J., and Liu, W. (2012). Matrix metalloproteinase-2-mediated occludin degradation and caveolin-1-mediated claudin-5 redistribution contribute to blood-brain barrier damage in early ischemic stroke stage. J. Neurosci. *32*, 3044–3057.

Love, M.I., Huber, W., and Anders, S. (2014). Moderated estimation of fold change and dispersion for RNA-seq data with DESeq2. Genome Biol. *15*, 550–621.

Lu, T.M., Houghton, S., Magdeldin, T., Durán, J.G.B., Minotti, A.P., Snead, A., Sproul, A., Nguyen, D.H.T., Xiang, J., Fine, H.A., et al. (2021). Pluripotent stem cell-derived epithelium misidentified as brain microvascular endothelium requires ETS factors to acquire vascular fate. Proc. Natl. Acad. Sci. USA *118*, e2016950118.

Malik, R., Chauhan, G., Traylor, M., Sargurupremraj, M., Okada, Y., Mishra, A., Rutten-Jacobs, L., Giese, A.-K., van der Laan, S.W., Gretarsdottir, S., et al. (2018). Multiancestry genome-wide association

study of 520,000 subjects identifies 32 loci associated with stroke and stroke subtypes. Nat. Genet. *51*, 1192–1193.

Mancuso, M., Arnold, M., Bersano, A., Burlina, A., Chabriat, H., Debette, S., Enzinger, C., Federico, A., Filla, A., Finsterer, J., et al. (2020). Monogenic cerebral small-vessel diseases: diagnosis and therapy. Consensus recommendations of the European Academy of Neurology. Eur. J. Neurol. *27*, 909–927.

Murray, L.S., Lu, Y., Taggart, A., Van Regemorter, N., Vilain, C., Abramowicz, M., Kadler, K.E., and Van Agtmael, T. (2014). Chemical chaperone treatment reduces intracellular accumulation of mutant collagen IV and ameliorates the cellular phenotype of a COL4A2 mutation that causes haemorrhagic stroke. Hum. Mol. Genet. *23*, 283–292.

Nichols, P., Urriola, J., Miller, S., Bjorkman, T., Mahady, K., Vegh, V., Nasrallah, F., and Winter, C. (2021). Blood-brain barrier dysfunction significantly correlates with serum matrix metalloproteinase-7 (MMP-7) following traumatic brain injury. Neuroimage: Clinical *31*, 102741.

Nitta, T., Hata, M., Gotoh, S., Seo, Y., Sasaki, H., Hashimoto, N., Furuse, M., and Tsukita, S. (2003). Size-selective loosening of the blood-brain barrier in claudin-5-deficient mice. J. Cell Biol. *161*, 653–660.

Orlova, V.v., Drabsch, Y., Freund, C., Petrus-Reurer, S., van den Hil, F.E., Muenthaisong, S., Dijke, P.T., and Mummery, C.L. (2014a). Functionality of endothelial cells and pericytes from human pluripotent stem cells demonstrated in cultured vascular plexus and zebrafish xenografts. Arterioscler. Thromb. Vasc. Biol. *34*, 177–186.

Orlova, V.v., van den Hil, F.E., Petrus-Reurer, S., Drabsch, Y., ten Dijke, P., and Mummery, C.L. (2014b). Generation, expansion and functional analysis of endothelial cells and pericytes derived from human pluripotent stem cells. Nat. Protoc. *9*, 1514–1531.

Pan, R., Yu, K., Weatherwax, T., Zheng, H., Liu, W., and Liu, K.J. (2017). Blood Occludin Level as a Potential Biomarker for Early Blood Brain Barrier Damage Following Ischemic Stroke. Sci. Rep. *7*, 40331.

Persyn, E., Hanscombe, K.B., Howson, J.M.M., Lewis, C.M., Traylor, M., and Markus, H.S. (2020). Genome-wide association study of MRI markers of cerebral small vessel disease in 42,310 participants. Nat. Commun. *11*, 2175.

Pires, P.W., Rogers, C.T., Mcclain, J.L., Garver, H.S., Fink, G.D., and Dorrance, A.M. (2011). Doxycycline, a matrix metalloprotease inhibitor, reduces vascular remodeling and damage after cerebral ischemia in stroke-prone spontaneously hypertensive rats. Am. J. Physiol. Heart Circ. Physiol. *301*, 87–97.

Pokhilko, A., Brezzo, G., Handunnetthi, L., Heilig, R., Lennon, R., Smith, C., Allan, S.M., Granata, A., Sinha, S., Wang, T., et al. (2021). Global proteomic analysis of extracellular matrix in mouse and human brain highlights relevance to cerebrovascular disease. J. Cerebr. Blood Flow Metabol. *41*, 2423–2438.

Rannikmäe, K., Davies, G., Thomson, P.A., Bevan, S., Devan, W.J., Falcone, G.J., Traylor, M., Anderson, C.D., Battey, T.W.K., Radmanesh, F., et al. (2015). Common variation in COL4A1/COL4A2 is associated with sporadic cerebral small vessel disease. Neurology *84*, 918–926.

Ratelade, J., Mezouar, N., Domenga-Denier, V., Rochey, A., Plaisier, E., and Joutel, A. (2018). Severity of arterial defects in the retina correlates with the burden of intracerebral haemorrhage in COL4A1-related stroke. J. Pathol. *244*, 408–420.

Ratelade, J., Klug, N.R., Lombardi, D., Angelim, M.K.S.C., Dabertrand, F., Dabertrand, F., Domenga-Denier, V., Salman, R.A.S., Smith, C., Gerbeau, J.F., et al. (2020). Reducing Hypermuscularization of the Transitional Segment between Arterioles and Capillaries Protects against Spontaneous Intracerebral Hemorrhage. Circulation *141*, 2078–2094.

Roach, D.M., Fitridge, R.A., Laws, P.E., Millard, S.H., Varelias, A., and Cowled, P.A. (2002). Up-regulation of MMP-2 and MMP-9 Leads to Degradation of Type IV Collagen During Skeletal Muscle Reperfusion Injury; Protection by the MMP Inhibitor, Doxycycline. Eur. J. Vasc. Endovasc. Surg. *23*, 260–269.

Rosell, A., Cuadrado, E., Ortega-Aznar, A., Hernández-Guillamon, M., Lo, E.H., and Montaner, J. (2008). MMP-9-positive neutrophil infiltration is associated to blood-brain barrier breakdown and basal lamina type IV collagen degradation during hemorrhagic transformation after human ischemic stroke. Stroke *39*, 1121–1126.

Serrano, F., Bernard, W.G., Granata, A., Iyer, D., Steventon, B., Kim, M., Vallier, L., Gambardella, L., and Sinha, S. (2019). A Novel Human Pluripotent Stem Cell-Derived Neural Crest Model of Treacher Collins Syndrome Shows Defects in Cell Death and Migration. Stem Cell. Dev. *28*, 81–100.

Shah, S., Kumar, Y., McLean, B., Churchill, A., Stoodley, N., Rankin, J., Rizzu, P., van der Knaap, M., and Jardine, P. (2010). A dominantly inherited mutation in collagen IV A1 (COL4A1) causing childhood onset stroke without porencephaly. Eur. J. Paediatr. Neurol. *14*, 182–187. https://doi.org/10.1016/J.EJPN.2009.04.010.

Siitonen, M., Börjesson-Hanson, A., Pöyhönen, M., Ora, A., Pasanen, P., Bras, J., Kern, S., Kern, J., Andersen, O., Stanescu, H., et al. (2017). Multi-infarct dementia of Swedish type is caused by a 3'UTR mutation of COL4A1. Brain *140*, e29.

Smith, E.E., and Markus, H.S. (2020). New Treatment Approaches to Modify the Course of Cerebral Small Vessel Diseases. Stroke *51*, 38–46.

Thomas, A.L., and Steward, W.P. (2005). Marimastat: the clinical development of a matrix metalloproteinase inhibitor. Expet Opin. Invest. Drugs *9*, 2913–2922. https://doi.org/10.1517/13543784.9.12.2913.

Traylor, M., Persyn, E., Tomppo, L., Klasson, S., Abedi, V., Bakker, M.K., Torres, N., Li, L., Bell, S., Rutten-Jacobs, L., et al. (2021). Genetic basis of lacunar stroke: a pooled analysis of individual patient data and genome-wide association studies. Lancet Neurol. *20*, 351–361.

Underly, R.G., Levy, M., Hartmann, D.A., Grant, R.I., Watson, A.N., and Shih, A.Y. (2017). Pericytes as Inducers of Rapid, Matrix Metalloproteinase-9-Dependent Capillary Damage during Ischemia. J. Neurosci. *37*, 129–140.

Verdura, E., Hervé, D., Bergametti, F., Jacquet, C., Morvan, T., Prieto-Morin, C., Mackowiak, A., Manchon, E., Hosseini, H., Cordonnier, C., et al. (2016). Disruption of a miR-29 binding site leading to COL4A1 upregulation causes pontine autosomal dominant

microangiopathy with leukoencephalopathy. Ann. Neurol. *80*, 741–753.

Wallin, A., Kapaki, E., Boban, M., Engelborghs, S., Hermann, D.M., Huisa, B., Jonsson, M., Kramberger, M.G., Lossi, L., Malojcic, B., et al. (2017). Biochemical markers in vascular cognitive impairment associated with subcortical small vessel disease - A consensus report. BMC Neurol. *17*, 102–112.

Wardlaw, J.M., Allerhand, M., Doubal, F.N., Valdes Hernandez, M., Morris, Z., Gow, A.J., Bastin, M., Starr, J.M., Dennis, M.S., and Deary, I.J. (2014). Vascular risk factors, large-artery atheroma, and brain white matter hyperintensities. Neurology *82*, 1331–1338.

Wardlaw, J.M., Smith, C., and Dichgans, M. (2019). Small vessel disease: mechanisms and clinical implications. Lancet Neurol. *18*, 684–696.

Wu, G., and Haw, R. (2017). Functional interaction network construction and analysis for disease discovery. Methods Mol. Biol. *1558*, 235–253.

Yang, Y., Estrada, E.Y., Thompson, J.F., Liu, W., and Rosenberg, G.A. (2007). Matrix metalloproteinase-mediated disruption of tight junction proteins in cerebral vessels is reversed by synthetic matrix metalloproteinase inhibitor in focal ischemia in rat. J. Cerebr. Blood Flow Metabol. *27*, 697–709.

Stem Cell Reports, Volume *18*

Supplemental Information

A novel human iPSC model of COL4A1/A2 small vessel disease unveils a key pathogenic role of matrix metalloproteinases

Maha Al-Thani, Mary Goodwin-Trotman, Steven Bell, Krushangi Patel, Lauren K. Fleming, Catheline Vilain, Marc Abramowicz, Stuart M. Allan, Tao Wang, M. Zameel Cader, Karen Horsburgh, Tom Van Agtmael, Sanjay Sinha, Hugh S. Markus, and Alessandra Granata

Supplemental Information

Supplemental Figures and Legends

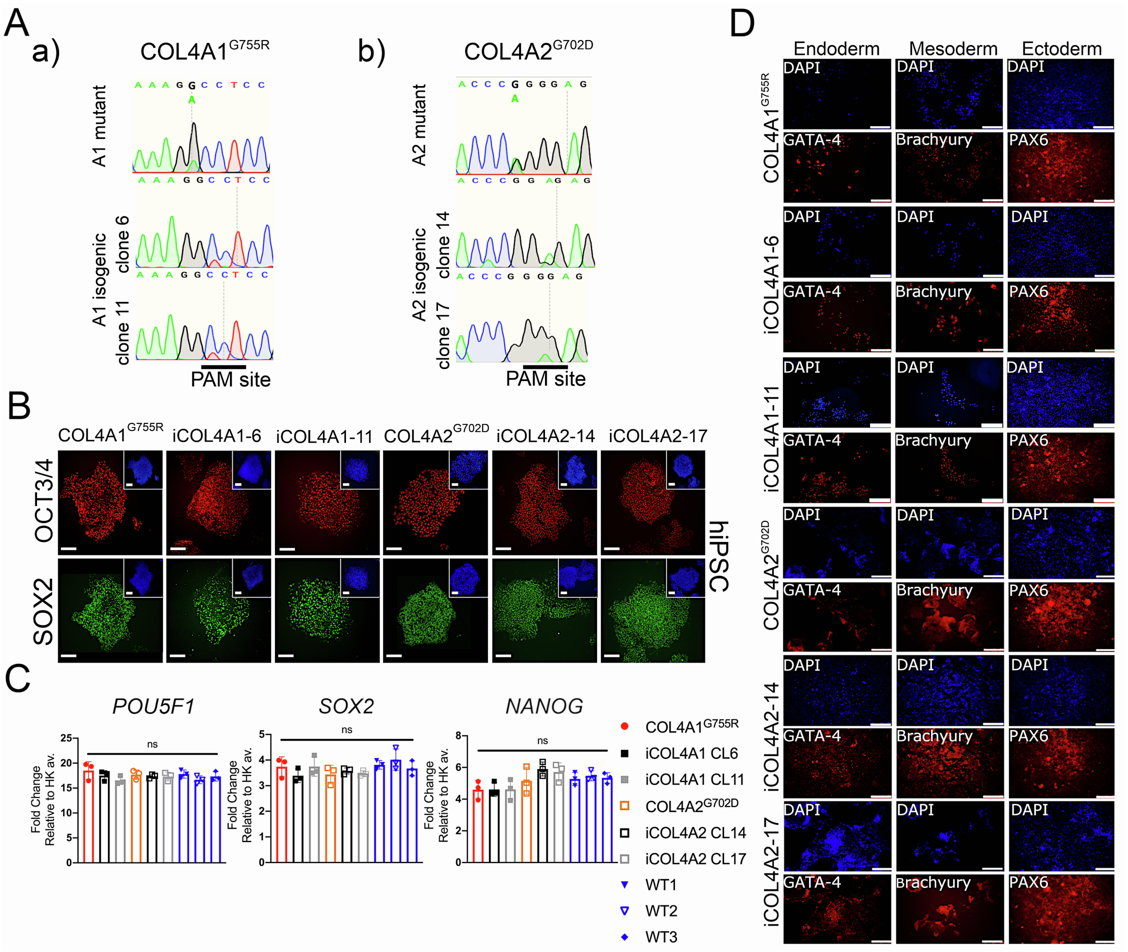

Figure S1. Characterization for COL4A1*^{G755R}*, COL4A2*^{G702D}* and isogenic hiPSC lines.

A) Sanger sequencing output for **(a)** *COL4A1* heterozygous mutation (G775R) and CRISPR-corrected isogenic A1 clone 6 and clone 11 and **(b)** *COL4A2* heterozygous mutation (G702D) and CRISPR-corrected isogenic A2 clone 14 and clone 17. **B)** Immunostaining analysis for hiPSC markers (OCT3/4 and SOX2) for *COL4A1^{G755R}*, *COL4A2^{G702D}* and isogenic A1 (iCOL4A1-6 and -11) and A2 (iCOL4A2-14 and -17); nuclei were stained with DAPI (insert); scale bar=100µm. **C)** Quantitative real-time PCR analysis for pluripotency markers expression (*POU5F1*, *SOX2* and *NANOG*); the results are presented as means ± SD of 3 independent experiments. **D)** Immunostaining analysis for each of the three germ layers (GATA-4, endoderm; BRACHYURY, mesoderm; PAX6, ectoderm); nuclei were stained with DAPI; scale bar=100µm. hiPSC= induced pluripotent stem cells. PAM site= protospacer adjacent motif sequence. The results are presented as means ± SD of 3 independent experiments; ns (not significant). Statistical analysis was performed by 2-way ANOVA with Tukey's multiple comparison test.

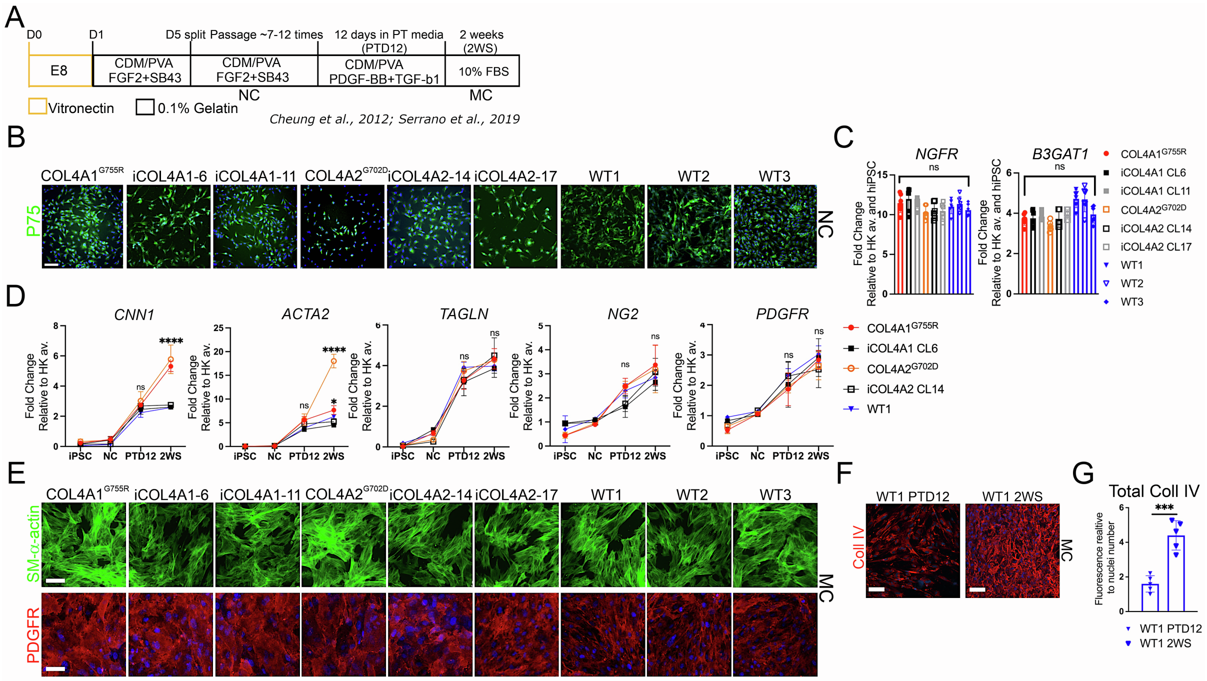

Figure S2. Neural-crest derived mural cells differentiation and characterization. A) Schematic of neural crest (NC) derived mural cells (MC) differentiation. Characterization of NC intermediate population by **(B)** immunostaining for specific marker (p75) and **(C)** quantitative real-time PCR for *NGFR* (P75) and *B3GAT1* (HNK1) for COL4A1^{G755R}, COL4A2^{G702D}, two isogenic sub-clones for A1 (iCOL4A1-CL6 and CL11) and A2 (iCOL4A2-CL14 and Cl-17) and three independent healthy controls (WT1, WT2 and WT3; see also **Table S1**) (n=6). **D)** Representative time-course quantitative real-time PCR of hiPSC-MC differentiation for specific markers: *CNN1, ACTA2, TAGLN, NG2, PDGFR* at hiPSC, NC and MC at day 12 of PDGFBB+TFG-β1 differentiation (PTD12) and after 2 weeks of culture in serum-containing media (2WS) for COL4A1/2, iCOL4A1-CL6, iCOL4A2-CL14 and WT1 (n=3). **E)** Immunostaining for smooth muscle alpha actin (SM α-actin) and PDGF Receptor Β (PDGFRB) in hiPSC-derived MC for COL4A1^{G755R}, COL4A2^{G702D}, 2 isogenic sub-clones and WT controls. **F)** Immunostaining for collagen IV in MC at early stage (PTD12) of differentiation and at late stage (2WS) and **(G)** quantification of n= 6 biological replicates. Nuclei were stained with DAPI; scale bar=100μm. NC=neural crest; MC=mural cells. The results are presented as means ± SD of n independent experiments; *P<0.05; ***P<0.001; ****P<0.0001; ns (not significant). Statistical analysis was performed by unpaired Student's t test for two-group comparisons or 2-way ANOVA with Tukey's multiple comparison test.

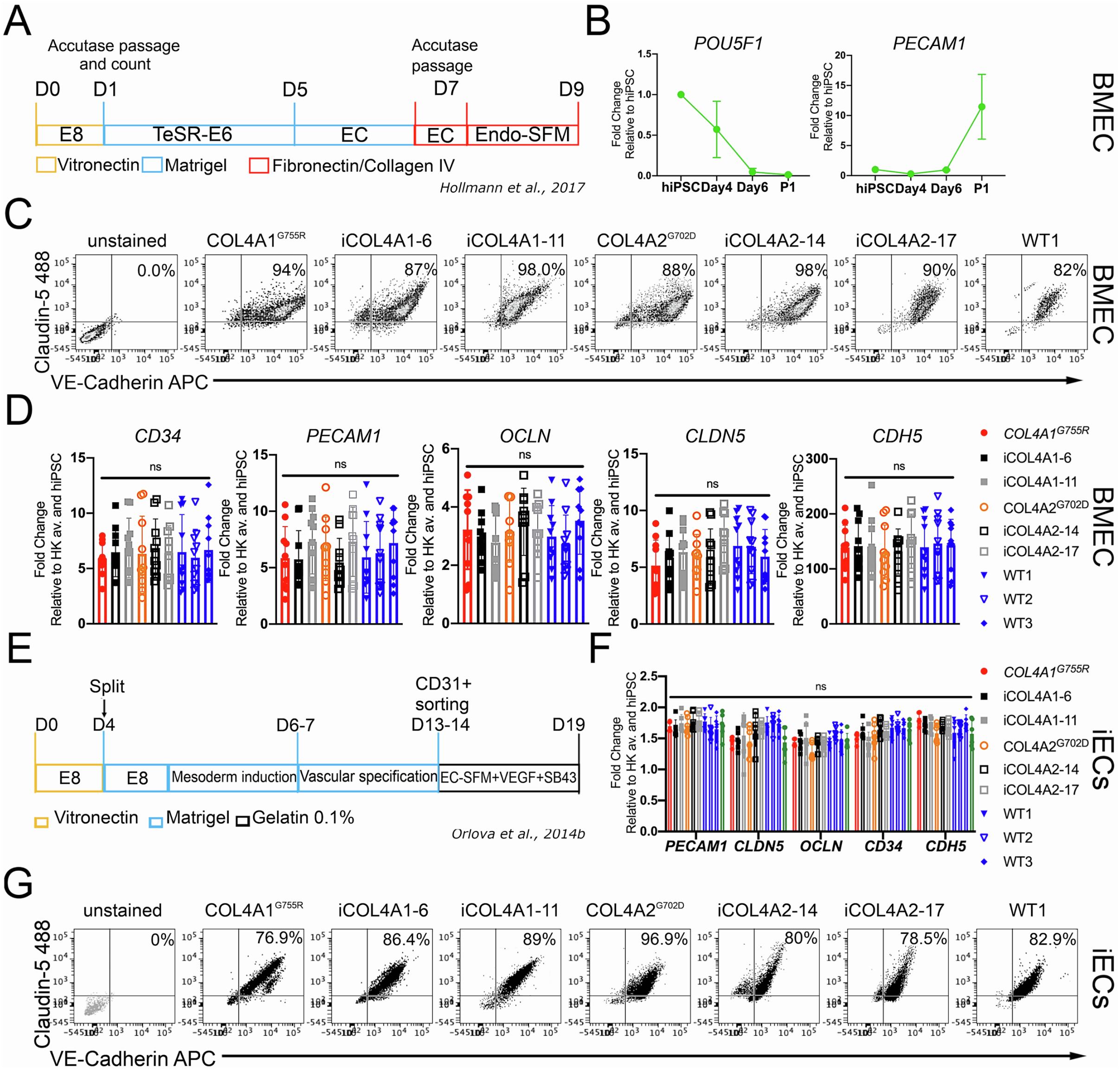

Figure S3. hiPSC-derived brain microvascular endothelial-like cells and endothelial cells differentiation and characterization.

A) Schematic of brain microvascular endothelial-like cells (BMEC) differentiation from hiPSC. **B)** Representative time-course quantitative real-time PCR of WT1 hiPSC-BMEC differentiation for pluripotent marker (*POU5F1*) and endothelial marker (*PECAM1*). **C)** Flow cytometric analysis of hiPSC-BMEC for VE-cadherin (APC conjugated) and claudin-5 (488 conjugated) for *COL4A1*^{G755R}, *COL4A2*^{G702D}, isogenic sub-clones and WT1. **D)** mRNA profile of hiPSC-BMEC by quantitative real-time PCR for specific markers (*CD34, PECAM1, OCLN, CDH5 and CLDN5*) (n=10). **E)** Schematic of hiPSC-endothelial cells (iECs) differentiation and characterization by **(F)** quantitative real-time PCR for endothelial markers: *PECAM1, CLDN5, OCLN, CD34* and *CDH5* for iECs lines and HUVEC (n=6) and **(G)** flow cytometry for VE-cadherin APC and claudin-5 488 double staining. HUVEC = Human umbilical vein endothelial cells. The results are presented as means ± SD of n independent experiments; ns (not significant).

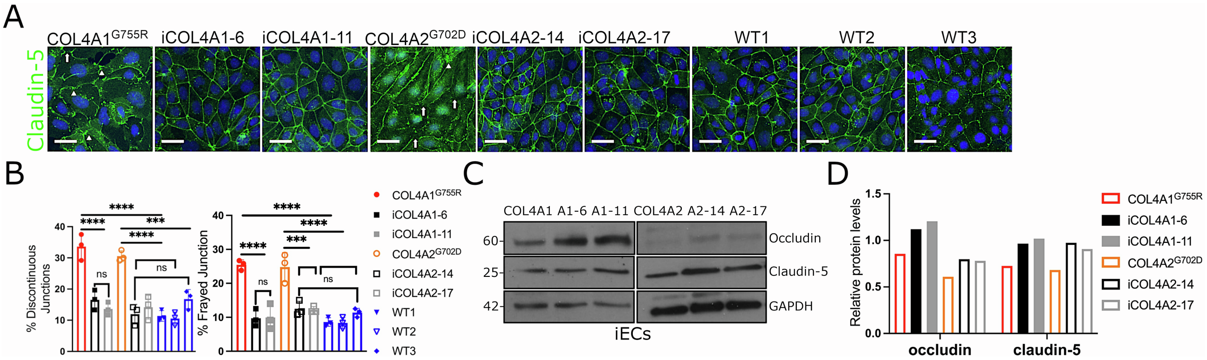

Figure S4. hiPSC-derived ECs showing tight junction abnormalities.
A-B) Immunostaining for claudin-5 shows increased discontinuity and frayed junctions in *COL4A1/A2* hiPSC-ECs compared to isogenic sub-clones and WT controls (n=3). Nuclei were stained with DAPI; scale bar=100µm. **C)** Western blotting for occludin and claudin-5 in iECs and (**D**) quantification relative to housekeeping (GAPDH) shows decreased protein levels in mutant lines compared to the isogenic control (representative blot of n=2). The results are presented as means ± SD of n independent experiments; ***P<0.001; ****P<0.0001; ns (not significant). Statistical analysis was performed by 2-way ANOVA with Tukey's multiple comparison test.

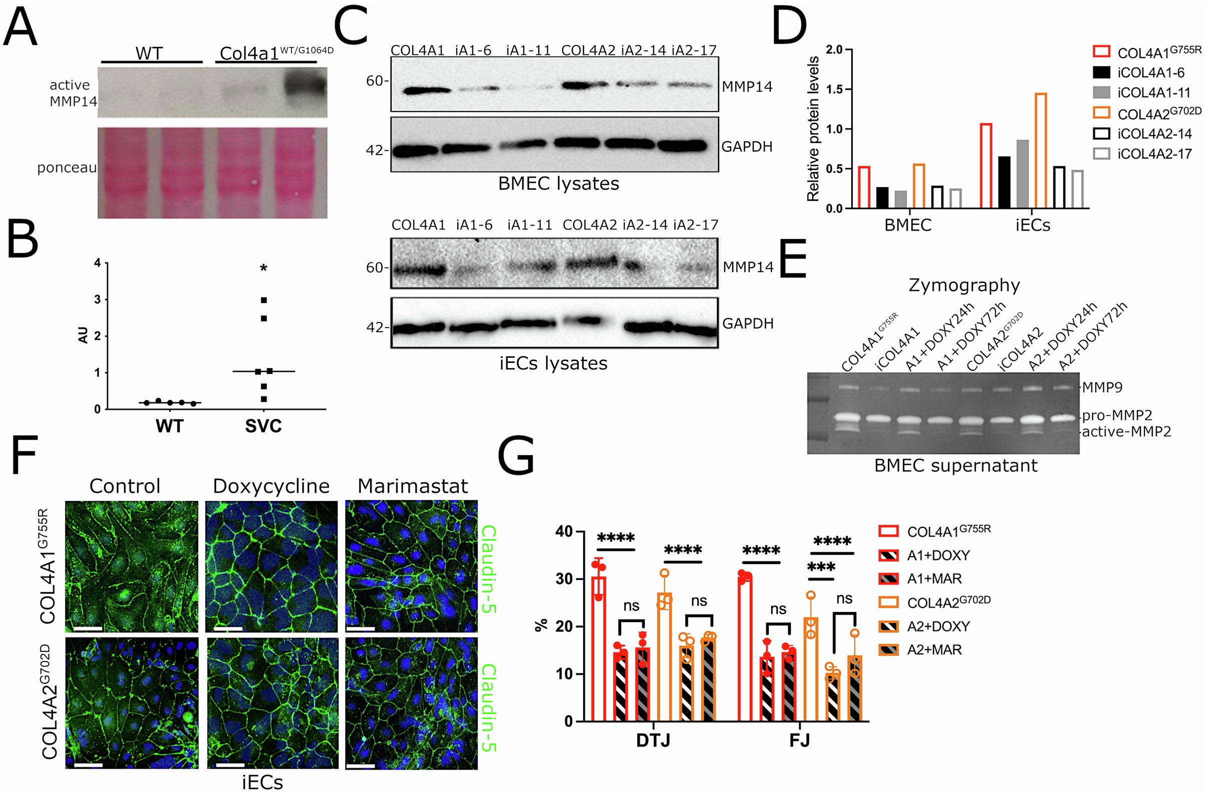

Figure S5. MMP14 is increased in Col4a1 mouse aorta and hiPSC-derived BMEC and iECs

A-B) Total Mmp14 protein level found higher in Col4a1 mice aorta (n=6) compared to WT mice (n=5). **C-D)** Protein blots and quantification showing MMP14 upregulation in *COL4A1/A2* BMEC and iECs compared to isogenic sub-clones (representative blots of n=4). **E)** Zymography analysis of BMEC supernatants show higher MMP9 and MMP2 activity levels in mutant *COL4A1/A2* compared to isogenics, which decrease in response to doxycycline (8 uM) mediated MMPs inhibition at 72 hours (DOXY72h) treatment. **F-G)** Discontinuity (DTJ) and frayed junctions (FJ) in *COL4A1/A2* iECs showed by staining for claudin-5 is reverted upon doxycycline and marimastat (MAR; n=3) treatment compared to control (DMSO). Nuclei were stained with DAPI; scale bar=100µm. The results are presented as means ± SD of n independent experiments *P<0.05; ***P<0.001; ****P<0.0001; ns (not significant). Statistical analysis was performed by 2-way ANOVA with Tukey's multiple comparison test.

Tables

Table S1. List of hiPSC lines used in this study.

Name	Vendor or Source	Sex and age	Individual	URL and Reference	Reprogram method/gene editing
WT1 (HPSI0414i-seru_7)	HIPSCI Consortium	F<br>65-69		https://www.hipsci.org/lines/#/lines/HPSI0414i-seru_7	Sendai virus
WT2 (HPSI0314i-sojd_3)	HIPSCI Consortium	F<br>45-49		https://www.hipsci.org/lines/#/lines/HPSI0314i-sojd_3	Sendai virus
WT3 (HPSI0214i-wibj_2)	HIPSCI Consortium	F<br>55-59		https://www.hipsci.org/lines/#/lines/HPSI0214i-wibj_2	Sendai virus
COL4A1^{G755R} Clone 4 and 5	iPS Core Facility, Cambridge	F<br>65	SVD patient	(Shah et al)	Sendai virus
COL4A2^{G702D}	-	M<br>75	Father of SVD patient	(Murray et al)	Sendai virus
iCOL4A1 clone 6 and 11	-	F<br>65		-	CRISPR/Cas9 edited
iCOL4A2 clone 14 and 17	-	M<br>75		-	CRISPR/Cas9 edited

Table S2. CRISPR sgRNA guide and ssODN related to Figure S1.

Gene/Mutation	Sequencing primers sequence 5'-3'	gRNA	Donor sequence 5'-3' (ssODN) Corrected base Mutated PAM
COL4A1^{G755R}	GCTTGAAAAGGGTTGAGCAG	CCGGCATTCCTGGCACACCC	G*A*C*TCAAAGGTTTGCCAGGTCTTCCCGGCATTCCTGGCACA CCC**G**G**A**GAGAAGGGGAGCATTGGGGTACCAGGCGTTCCTGGAGAAC*A*T*G
COL4A2^{G702D}	TCCAGTCCGTAAACAGGATTT	CGAAGCCUGGGAUUCCUCGG	G*C*C*TGATGTGGTTTGTGGTTTATTTGGTTATTTAGGTGCCAAAG**GT**CTCCGAGGAATCCCAGGCTTCGCAGGAGCTGATGGAGGAC*C*A*G

Table S3. Quantitative real-time PCR Primers set used in this study, related to Figure 1, Figure 4, Figure S1, Figure S2 and Figure S3.

**Gene Target**	**Forward Sequence 5'-3'**	**Reverse Sequence 5'-3'**
GAPDH	AACAGCCTCAAGATCATCAGC	GGATGATGTTCTGGAGAGCC
HMBS (PBGD)	GGAGCCATGTCTGGTAACGG	CCACGCGAATCACTCTCATCT
POU5F1 (OCT4)	AGGGCAAGCGATCAAGCA	GGAAAGGGACCGAGGAGTA
SOX2	ATGCACCGCTACGACGTGA	CTTTTGCACCCCTCCCATTT
NANOG	ACTAACATGAGTGTGGATCC	TCATCTTCACACGTCTTTCAG
PECAM1	CAGGCGCCGGGAGAAGTGAC	CGTCCAGTCCGGCAGGCTCT
CD34	CACAGGAGAAAGGCTGGGCGA	TGGCCGTTTCTGGAGGTGGC
OCLN	GGAGTGAACCCAACTGCTCA	CTCCTGGGGATCCACAACAC
CDH5	GGTCAAACTGCCCATACTTG	CGCAATAGACAAGGACATAACAC
CLDN5	CAGTACCGCAGGAAGAGGAG	ATCCCATGGCAAACAGAGAG
CHD5	CTCTGGGAGTGAGTGGAAGC	CCTGAGGATGATGGGAAAGA
MMP2	TCTCCTGACATTGACCTTGGC	CAAGGTGCTGGCTGAGTAGATC
MMP9	TTGACAGCGACAAGAAGTGG	GCCATTCACGTCGTCCTTAT
MMP14	CAGAGAAGGCACACAAACGA	CACTGGTGAGACAGGCTTGA
CNN1	GTCCACCCTCCTGGCTTT	AAACTTGTTGGTGCCCATCT
P75	ACAAGACCTCATAGCCAGCAC	CTGTTGGCTCCTTGCTTGTTC
CSPG4 (NG2)	TTCCAGCTGAGCATGTCTGA	TCCTCCCGATCTGAAACCAC
PDGFRB	GCTTAAATCCACAGCCCGCA	AGGTAGTCCACCAGGTCTC

Table S4. Primary antibodies list used in this study, related to Figure1, Figure 3, Figure 5, Figure S1, Figure S2, Figure S3, Figure S4 and Figure S5.

Target antigen	Species	Supplier	Catalogue n.	Use
OCT3/4	Mouse	Santa Cruz	SC-5279	Immunofluorescence
SOX2	Mouse	Abcam	sc-21705	Immunofluorescence
TRA-1-60	Rabbit	R&D Systems	AF2018-SP	Immunofluorescence
GATA-4	Mouse	Santa Cruz	sc-25310	Immunofluorescence
Brachyury	Mouse	Santa Cruz	sc-166962	Immunofluorescence
Occludin	Mouse	Thermo Fisher	331500	Immunofluorescence<br>Western blotting
claudin-5	Rabbit	Abcam	ab15106	Immunofluorescence<br>Western blotting
P75	Rabbit	Abcam	ab8874	Immunofluorescence
Smooth Muscle Actin	Mouse	Agilent	M085101-2	Immunofluorescence
SM22	Rabbit	Abcam	ab14106	Immunofluorescence
Calponin	Mouse	Sigma-Aldrich	C-2687	Immunofluorescence
NG2	Rabbit	Sigma-Aldrich	AB5320	Immunofluorescence
PDGF Receptor beta	Rabbit	Cell Signaling	3169	Immunofluorescence
Collagen IV	Rabbit	Abcam	ab6586	Immunofluorescence
claudin-5 pre-conjugated AF488	Mouse	Thermo Fisher	352588	Flow cytometry
CD144 (VE-cadherin) APC-conjugated	Mouse	Thermo Fisher	17-1441-80	Flow cytometry
IgG1 Isotype Control FITC-conjugated	Mouse	Thermo Fisher	GM4992	Flow cytometry
IgG1kappa Isotype Control APC-conjugated	Mouse	R&D Systems	IC002A	Flow cytometry
Annexin V-488	-	Life technologies	V13241	Flow cytometry
Propidium Iodide (PI)	-	Life technologies		Flow cytometry
β-Actin	Mouse	Sigma-Aldrich	A1978	Western blotting
MMP14	Rabbit	Abcam	ab51074	Western blotting
GAPDH	Mouse	Abcam	Ab8245	Western blotting

Table S5. List of identified ECM differentially expressed genes (DEGs) in *COL4A1/A2* vs isogenic MC, related to Figure 4.

GENE	log2FoldChange	LOG FDR	ECM
**ZNF536**	4.95529102016929	4.14E+00	1
**NCAM1**	4.11174320070316	3.82E+00	1
**MMP7**	3.97688493423821	1.69E+00	1
**ANKS1B**	3.88805632439505	3.68E+00	1
**DLGAP1**	3.87359008701633	3.60E+00	1
**PRODH**	3.35816928153035	2.14E+00	1
**LGR5**	3.34382833835286	1.86E+00	1
**LAMA3**	3.16127049352719	5.22E+00	1
**CADM2**	3.11452675789334	1.88E+00	1
**ELMO1**	3.04557087794061	3.14E+00	1
**KCNA2**	2.92787394337378	1.98E+00	1
**ABCB1**	2.80072080613374	1.71E+00	1
**GPR158**	2.67672768137061	1.98E+00	1
**CNTN1**	2.64145308178325	1.86E+00	1
**LIMCH1**	2.6119812320724	1.86E+00	1
**ALDH1L1**	2.52924831679259	1.83E+00	1
**TAGLN3**	2.40089796135524	1.79E+00	1
**MMP15**	2.3017540760174	2.77E+00	1
**CNNM1**	2.14528940341127	2.38E+00	1
**ATP1A2**	2.07014110537751	1.37644255215566	1
**SH3GL2**	2.02674427698518	3.32709887309457	1
**SH3GL3**	1.96493467263382	1.33503127079419	1
**AP3B2**	1.91281198134199	2.14678343169756	1
**LONRF2**	1.86610002336086	2.04730604450956	1
**LAMA1**	1.74955128715685	1.58968870650893	1
**MMP24**	1.72542160915493	1.36283160917736	1
**DOCK3**	1.7093082692694	1.66463712498211	1
**SBSPON**	1.62860413058366	1.50518527608545	1
**CLU**	1.61895548373455	1.58564688586111	1
**PPP1R9A**	1.48785411210524	1.79003207873103	1
**GAD1**	1.40416565564864	1.40405181192663	1
**COL4A6**	1.37761556652196	1.33709183759027	1
**SPTBN2**	1.34273321873787	1.46793184590654	1
**DCLK1**	1.30508213285984	1.40108978598792	1
**LGI3**	1.23212136740195	1.33709183759027	1
**RBFOX3**	1.2301953062032	1.84310680196793	1
**CORO2B**	1.15992146465967	3.55721027028743	1
**ANK2**	0.882039273617303	4.37869352390178	1
**CASK**	0.837906889789319	1.54614802467899	1
**NBEA**	0.708993913453233	1.326775846766	1
**ASAH1**	0.643114654921668	1.97718682074561	1
**ITPR2**	0.456699641255665	2.47578039450843	1
**DNAJC9**	0.431760286525865	3.43480323008621	1
**PHIP**	0.405822504148944	1.32937067329303	1
**PRDX6**	0.383723822	1.30369531898155	1
**ATP6V1F**	0.315755603918921	1.58314577498045	1
**NFS1**	0.220249262501158	1.78682423843528	1
**SHROOM2**	0.206055478537332	0.981122182373769	1
**STX12**	0.123459940152881	0.330434873025838	1
**RTN1**	0.0799153826051542	1.43005411626271	1
**MYOF**	0.275804043	1.66463713293907	1
**SELENBP1**	-2.96E-07	1.44731570190812	1

**LAMP5**	1.56E-06	1.85572241272765	1
**ELN**	-1.52E-06	2.47578039450843	1
**ICAM5**	-0.175606968002257	1.60858811632863	1
**MMP2**	-0.716063799342786	1.84392735543628	1
**MYL9**	-0.948504723731799	1.68499291515606	1
**ANGTP1**	-1.4204553	1.79003208	1
**PECAM1**	-2.30097954443201	1.51805900595662	1

Table S6. The Reactome pathways analysis of the identified ECM DEGs, related to Figure 4.

Pathway identifier	Pathway name	pValue	Gene ID
**R-HSA-1592389**	Activation of Matrix Metalloproteinases	1.7E-08	MMP24;MMP7;MMP15;MMP2
**R-HSA-1474244**	Extracellular matrix organization	3.3E-08	MMP24;MMP7;MMP15;LAMA1;MMP2;LAMA3; COL4A6;PECAM1;CASK;NCAM1
**R-HSA-1474228**	Degradation of the extracellular matrix	4.37E-07	MMP24;MMP7;MMP15;MMP2;LAMA3;COL4A6
**R-HSA-1442490**	Collagen degradation	8.81E-07	MMP7;MMP15;MMP2;COL4A6
**R-HSA-2022090**	Assembly of collagen fibrils and other multimeric structures	1.57E-05	MMP7;LAMA3;COL4A6
**R-HSA-373760**	L1CAM interactions	3.17E-05	LAMA1;CNTN1;NCAM1;ANK2;SH3GL2;SPTBN2
**R-HSA-1474290**	Collagen formation	1.24E-04	MMP7;LAMA3;COL4A6
**R-HSA-3000171**	Non-integrin membrane-ECM interactions	1.91E-04	LAMA1;LAMA3;COL4A6;CASK
**R-HSA-9022927**	MECP2 regulates transcription of genes involved in GABA signaling	3.68E-04	GAD1
**R-HSA-3000157**	Laminin interactions	4.16E-04	LAMA1;LAMA3;COL4A6
**R-HSA-6785807**	Interleukin-4 and Interleukin-13 signaling	4.33E-04	MMP7;MMP2
**R-HSA-3000178**	ECM proteoglycans	5.04E-04	LAMA1;LAMA3;COL4A6;NCAM1
**R-HSA-6806834**	Signaling by MET	7.52E-04	SH3GL3;LAMA1;LAMA3;SH3GL2
**R-HSA-9009391**	Extra-nuclear estrogen signaling	0.001760099	MMP7;MMP2
**R-HSA-70688**	Proline catabolism	0.001955051	PRODH
**R-HSA-9006934**	Signaling by Receptor Tyrosine Kinases	0.002171418	SH3GL3;DOCK3;LAMA1;LAMA3;ELMO1;ITPR2; SH3GL2;ATP6V1F
**R-HSA-2214320**	Anchoring fibril formation	0.002237685	LAMA3;COL4A6
**R-HSA-5578775**	Ion homeostasis	0.003286605	ITPR2;ATP1A2
**R-HSA-8875360**	InlB-mediated entry of Listeria monocytogenes into host cell	0.003548008	SH3GL3;SH3GL2
**R-HSA-422475**	Axon guidance	0.005344994	LAMA1;MMP2;CNTN1;NCAM1;ANK2; SH3GL2;SPTBN2
**R-HSA-6807004**	Negative regulation of MET activity	0.006034786	SH3GL3;SH3GL2
**R-HSA-8876384**	Listeria monocytogenes entry into host cells	0.006997575	SH3GL3;SH3GL2
**R-HSA-9675108**	Nervous system development	0.007565737	LAMA1;MMP2;CNTN1;NCAM1;ANK2;SH3GL2;SPTBN2
**R-HSA-8874081**	MET activates PTK2 signaling	0.009685522	LAMA1;LAMA3
**R-HSA-445095**	Interaction between L1 and Ankyrins	0.010270056	ANK2;SPTBN2
**R-HSA-182971**	EGFR downregulation	0.012759693	SH3GL3;SH3GL2

**R-HSA-6807878**	COPI-mediated anterograde transport	0.013357171	USO1;ANK2;SPTBN2
**R-HSA-9768919**	NPAS4 regulates expression of target genes	0.015484716	RBFOX3
**R-HSA-8875878**	MET promotes cell motility	0.01843598	LAMA1;LAMA3
**R-HSA-9609736**	Assembly and cell surface presentation of NMDA receptors	0.021604588	NBEA;CASK
**R-HSA-1500931**	Cell-Cell communication	0.02404666	CADM2;LAMA3;CASK
**R-HSA-1489509**	DAG and IP3 signaling	0.024981882	NBEA;ITPR2
**R-HSA-3928665**	EPH-ephrin mediated repulsion of cells	0.026746145	MMP2
**R-HSA-888568**	GABA synthesis	0.027188048	GAD1
**R-HSA-5576891**	Cardiac conduction	0.03044768	ITPR2;ATP1A2
**R-HSA-8939211**	ESR-mediated signaling	0.030796928	MMP7;MMP2
**R-HSA-6794361**	Neurexins and neuroligins	0.031369098	CASK;DLGAP1
**R-HSA-9634815**	Transcriptional Regulation by NPAS4	0.031369098	RBFOX3
**R-HSA-9032759**	NTRK2 activates RAC1	0.031647996	DOCK3
**R-HSA-177929**	Signaling by EGFR	0.032329074	SH3GL3;SH3GL2
**R-HSA-166665**	Terminal pathway of complement	0.036087788	CLU
**R-HSA-112043**	PLC beta mediated events	0.038326293	NBEA;ITPR2
**R-HSA-199977**	ER to Golgi Anterograde Transport	0.040039399	USO1;ANK2;SPTBN2
**R-HSA-2161517**	Abacavir transmembrane transport	0.040507513	ABCB1
**R-HSA-164944**	Nef and signal transduction	0.040507513	ELMO1
**R-HSA-447043**	Neurofascin interactions	0.040507513	CNTN1
**R-HSA-166520**	Signaling by NTRKs	0.041257591	SH3GL3;DOCK3;SH3GL2
**R-HSA-375165**	NCAM signaling for neurite out-growth	0.041472104	NCAM1;SPTBN2
**R-HSA-936837**	Ion transport by P-type ATPases	0.04362168	ATP1A2
**R-HSA-112040**	G-protein mediated events	0.044711834	NBEA;ITPR2
**R-HSA-446107**	Type I hemidesmosome assembly	0.049287121	LAMA3
**R-HSA-9032500**	Activated NTRK2 signals through FYN	0.049287121	DOCK3
**R-HSA-216083**	Integrin cell surface interactions	0.04976213	COL4A6;PECAM1

Supplemental Experimental procedures

HiPSC culture

All the hiPSC lines use for this study are listed in **Table S1.** Wild-type (WT) hiPSC lines were purchased from the HiPSci Human stem cell initiative cell bank (https://www.hipsci.org). *COL4A1*^{G755R} hiPSC line was generated from skin biopsy from a SVD patient, recruited at the Stroke Research Group at the University of Cambridge (Ethics REC NO 16/EE/0118) and reprogrammed by the Cambridge iPSC core. COL4A2^{G702D} hiPSC line was obtained by Professor Tom Van Agtmael (Murray et al., 2014). Isogenic control lines for *COL4A1*^{G755R} (iCOL4A1) and *COL4A2*^{G702D} (iCOL4A2) were generated by CRISPR-gene editing method as described and two independent clones were used for each line (**Figure S1A)**. *COL4A1/A2* mutant and isogenic hiPSC lines were characterized for pluripotency markers expression by immunostaining and quantitative real-time PCR (**Figure S1B,C)** and by formation of the three germ-layers (**Figure S1D).** All hiPSC lines were cultured in TeSR™-E8 media (STEMCELL Technologies) or E8 media (Dulbecco's Modified Eagle Medium/Nutrient Mixture F-12 (DMEM/F-12) with Insulin-Transferrin-Selenium (Thermo Fisher Scientific), Sodium Bicarbonate (Thermo Fisher Scientific), and L-ascorbic acid (Merck) supplemented with FGF2 (4 ug/mL; Biochemistry Department, University of Cambridge) and TGF- β1 (1.74 ug/mL; R&D Systems) using Vitronectin XF (STEMCELL Technologies) as chemically defined xenofree cell culture matrix. All hiPSC lines were validated by the Cambridge Biomedical Research Centre iPSC core and routinely tested for presence of mycoplasma contamination by Mycoplasma Experience LTD.

HiPSC differentiation into mural cells

For Neural Crest (NC) differentiation, hiPSC were detached from Vitronectin coated plates using ReLeSR (STEMCELL Technologies) as previously described (Cheung et al., 2012; Serrano et al., 2019). Clumps were plated at a density of 300 in 0.1% gelatin-coated six well plates in CDM-polyvinyl alcohol (PVA) for 4 days without splitting. CDM was composed of Iscove’s modified Dulbecco’s medium plus Ham’s F12 NUT-MIX (Thermo Fisher Scientific) medium in a 1:1 ratio, supplemented with chemically defined lipid concentrate (Thermo Fisher Scientific), transferrin (Roche Diagnostics), insulin (Roche Diagnostics), and monothioglycerol (Sigma) supplemented with FGF2 (12 ng/mL; R&D Systems) and SB-431542 (10 mmol/L; Tocris), referred as FSB. At day 4, hiPSC was dissociated using TrypLE Express (Thermo Fisher) and seeded as single cells at a 1:3 ratio on 0.1% gelatin-coated plates in FSB. NC cells were passaged every time reached confluence, up to 12 passages.

For mural cells (MC) differentiation, NC cells were dissociated using TrypLE Express and cultured in MC differentiation medium (CDM-PVA supplemented with PDGF-BB (10 ng/ml, Peprotech) and TGF-β1 (2ng/ml, Peprotech) for 12 days (PTD12). For long-term cultures, MC were subsequently grown in MEM (Sigma-Aldrich M5650) containing 10% fetal bovine serum (FBS; Sigma-Aldrich F7524) up to 4 weeks. The majority of the experiments were performed after mural cells were culture in serum-containing media for 2 weeks (2WS).

HiPSC differentiation into BMEC and iECs

hiPSCs were differentiated to brain microvascular endothelial-like cells (BMEC) as previously described, with minor modifications (Hollmann et al., 2017). hiPSCs were washed once with 1X PBS (Corning®), dissociated with StemPro™ Accutase™ Cell Dissociation Reagent (Thermo Fisher Scientific) for 4 minutes, and collected by

centrifugation. hiPSCs were then resuspended in E8 medium containing 10 μM Y27632 (Tocris Bioscience) and seeded onto Matrigel-coated 6-well plates at a density of $1.56x10^4$ /cm^2. The following day, the cells were switched to TeSR™-E6 medium (Stem Cell Technologies) to initiate the differentiation. Media was changed every day for 4 days. On day 5, the cells were switched to Endothelial media (EC), which consisted of a basal human endothelial serum-free media (SFM; Thermo Fisher Scientific), supplemented with B27 (Fisher Scientific), basic fibroblast growth factor (bFGF; 10ng/ml; R&D Systems) and all-trans retinoic acid (RA; 10μM; Sigma-Aldrich). Cells were then left to incubate for 48 hours in EC medium without a media exchange. On day 6, resultant BMEC cells were washed with PBS and dissociated with accutase to single cells and plated at a density of $1.1x10^6$ /well on 12 well culture plate or $3.3x10^5$ /well on 24-well Transwells (CLS3470, Corning®) coated with collagen IV (from human placenta, 1mg/ml, Bornstein, and Traub Type IV; C5533, Sigma Aldrich) and fibronectin (from bovine plasma, 1mg/ml, F1141, Sigma Aldrich). 24 hours after plating, media was refreshed to EC medium without bFGF and RA. Subsequent media changes were performed every 2 days for 6 days.

hiPSC-ECs (iECs) were differentiated using a previously reported protocol with minor modifications (Orlova et al., 2014b). Briefly, hiPSCs were maintained in TeSR™-E8 medium on vitronectin-coated 6-well plates and seeded at day-1. Twenty-four hours after seeding E8 medium was replaced with B(P)EL medium supplemented with 8 μM CHIR. On day 3, the medium was replaced with B(P)EL medium supplemented with VEGF-A (50 ng/ml; Peprotech) and SB431542 (10 μM; Tocris Bioscience) and refreshed on days 6–9. iECs were isolated on day 10 by sorting using MiniMACS separator and CD34 MicroBead kit (Miltenyi Biotec).
iECs from cryo-preserved batches were used in all further experiments.
hiPSC-EC cells were thawed, resuspended in complete Endothelial cell serum-free medium (Gibco), and plated on a 0.1% gelatine-coated culture flask, as previously described. Cells were used for experiments when nearly confluent by visual inspection, typically on day 4. Cells were harvested using TrypLE™ according to the manufacturer's instructions.

HUVEC
HUVEC (Gibco™ C0035C, Thermo Fisher) were plated at a density of $2.5x10^3/cm^2$ in culture basal media (Thermo Fisher) in a T75 flask pre-coated overnight with collagen I (0.1% Type 1 collagen from calf skin, MERCK). Medium was changed daily and cells were passaged every 4-7 days using TrypLE express.

CRISPR-mediated gene editing.
To generate the isogenic line for *COL4A1^{G755R}* (iCOL4A1) and *COL4A2^{G702D}* (iCOL4A2), a CRISPR-gene editing method was performed using single guide synthetic RNA (sgRNA; Synthego), SpCas9 protein (Biochemistry Department, University of Cambridge), and a 90-nt single-stranded oligodeoxynucleotide (ssODN; IDT) for homology-directed repair (**Table S2**). To avoid ssODN cleavage by Cas9, a silent mutation was introduced in the NGG codon upstream of the correction site (**Figure S1A**). For gene targeting, 200,000 cells were electroporated with Cas9/sgRNA together with ssODN using the Amaxa 4DNucleofector CA-137 program code (Lonza). Transfected cells were plated onto vitronectin coated-plates in TeSR™-E8 media with 10 μM Y-27632 and CloneR (STEMCELL Technologies). After 48h, the pool of transfected cells was sequenced to test recombination efficiency. Positive

clones were selected by serial dilution and manual selection. Two sub-clones for each isogenic line were used for this study (**Table S1**).

Quantitative real-time polymerase chain reaction.

Complementary DNA (cDNA) was synthesized from 250 ng total RNA using the Maxima First Strand cDNA Synthesis Kit (Thermo Fisher Scientific). Quantitative real-time polymerase chain reaction (PCR) mixtures were prepared with the FAST-SYBR Green Master Mix (Thermo Fisher Scientific) and analyzed using the QuantStudio 7 Flex (Applied Biosystems, Thermo Fisher). Data are expressed as fold change (ΔΔCT) relative to the mean of GAPDH and PBGD housekeeping genes and to the level in hiPSCs of the same cell line.

Primer sequences are listed in **Table S3.**

Immunofluorescence staining and quantification

Adherent cells were fixed using 4% PFA (Boster) for 5 minutes at RT (hiPSC and MC) or 100% ice cold Methanol (BMEC and iECs) for 15 minutes at -20°C and then washed 3 times with 1X PBS containing Calcium and Magnesium (Oxoid). Cells were permeabilized with 0.05% Triton X-100 (Sigma) in PBS and blocked with PBS +3% BSA or 10% FBS for 60 min at RT.

For the detection of collagen IV in the ECM, 250,000 mural cells were plated per wells in a 12-wells plate and after 2 weeks of culture in serum-containing media, ECM was isolated by performing decellularization using 20mM of ammonium hydroxide to lyse the cells. Upon decellularization, matrix was fixed with 4% PFA and then incubated in blocking solution without permeabilization. Primary antibodies (1:200; **Table S4**) incubations were performed at 4°C overnight and Alexa Fluor tagged secondary antibodies (1:400, Molecular Probes Invitrogen) and DAPI (Sigma-Aldrich) applied for 1 hour at room temperature the following day. Images were acquired on a Zeiss LSM 700 confocal and Leica TCS SP5 microscopes and analyzed with Fiji-ImageJ software.

Quantification of fluorescence intensity for collagen IV was performed by taking the mean pixel intensity (Integrated Density, threshold 75-170) from an average of 3-5 fields of view from the same well. For tight junctions quantification in hiPSC-BMEC and iECs, following immunostaining with occludin or claudin-5 antibodies, cells that lacked at least one continuous junction or show one frayed area were classified as discontinuous as previously described (Lee et al., 2018). Images were processed in Fiji-ImageJ software with a minimum of 5 fields with approximately 30 cells/field from three separate differentiations were quantified and all experimental groups remained blinded until completion of the study. All images are representative images.

Western blotting

Cells were lysed in RIPA buffer with added phosphatase inhibitor cocktail (Sigma) and protease inhibitor cocktail (Sigma) on ice for 15min. Protein content was quantified by Pierce Bicinchoninic Acid (BCA) Protein Assay Kit (Thermo Fisher Scientific). Samples (10-20ng) was resolved by electrophoresis on 10-15% Tris-HCl precast sodium dodecyl sulfate (SDS)-polyacrylamide gel (Bio-Rad), then transferred to polyvinylidine difluoride membranes (PVDF; Millipore). Membranes were blocked for 1 h at room temperature with 5% BSA in Tris-Buffered Saline containing 0.1% Tween-20 (TBS-T; Sigma) and incubated overnight with primary antibodies (**Table S4**) at 4°C. Membranes were washed with TBS-T, incubated with horseradish peroxidase (HRP)-conjugated secondary antibodies for 1 h at room temperature and developed with the

Pierce ECL2 western blotting substrate (Thermo Fisher Scientific) using X-ray Developer OPTIMAX (PROTEC GmbH & co) or the Gel Doc™ XR+ system (BioRad). The ImageLab™ Software (v5.2, BioRad) High Resolution programme with Signal Accumulation Mode was used to capture images at incremental exposure times. Anti-β-actin and GAPDH antibody was used as control for equal loading and transfer of the samples. Quantification of bands were performed by Fiji/ImageJ.

Zymography

Gelatin zymography was performed in 10% acrylamide gels containing gelatin (4mg/ml; Novex 10% Zymogram gels; ThermoFisher). SDS-PAGE was performed using Tris-glycine SDS sample and running buffers as described by the manufacturer. After electrophoresis, SDS was replaced by Triton X-100 (2.5%) to renaturate the gelatinases. Gels were incubated in Tris buffer containing NaCl and $ZnCl_2$ at 37°C for 24 h. Gels were then stained with Coomassie Blue solution, followed by de-staining (Methanol, Acetic Acid solution) and visualized by using Gel Doc™ XR+ system (BioRad).

BMEC/iECs Flow Cytometry

Confluent wells were disassociated using Accutase and filtered to a single cell solution through a 40μm cell strainer (Corning®, Fisher Scientific). The cell suspension was fixed using Fixation/Permeabilisation solution (BD Biosciences) at 4ºC for 10 minutes and washed twice in PBS + 10% FBS. Cells were re-suspended with primary antibodies or pre-conjugated antibodies and incubated for 30 minutes at 4ºC. Cells were then resuspended in PBS and measured with a BD LRSFortessa or Canto II Flow Cytometer (BD Bioscience). Flow cytometric data were analyzed with FCSalyzer 0.9.15-alpha software.

Annexin V apoptosis assay. 1 × 10^6 cells/ml were harvested and resuspended in 1× annexin-binding buffer and incubated with 5 μl of Annexin V–488 (Alexa Fluor 488 Annexin V/Dead Cell Apoptosis Kit; Life technologies) for 15 min at room temperature. Cells were then resuspended in PBS with the addition of propidium iodide (PI 1:300) and measured with a BD LRSFortessa Flow cytometer. Flow cytometric data were analyzed with FCSalyzer 0.9.15-alpha software.

Scratch migration assay

Cells were plated onto 12-well plates and allowed to form a confluent monolayer. The cell monolayer was then scratched in a straight line to make a ‘‘scratch wound’’ with a 1-mL pipette tip. Cells were maintained in DMEM and images of the closure of the scratch were captured at different time points as indicated. Cells were tracked using the Wound_healing_size_tool macro for Fiji/ImageJ.

Mouse aorta dissection and analysis

Animal studies were performed in accordance with UK Home Office regulations (Project license 70/8604). Animals were sacrificed using an increasing gradient of CO_2 according to UK Home Office guidelines, and the thoracic aorta was collected and snap frozen on dry ice. Tissue samples were homogenized using steel beads (Qiagen) in TissueLyser (Qiagen) in RIPA buffer containing protease (Complete Mini, Roche) and phosphatase inhibitors (PhosSTOP, Roche). Protein concentrations were assessed via Pierce BCA Protein Assay (ThermoFisher) and protein were separated

by SDS-PAGE (Mini-Protein Biorad). Membranes were blocked with 5% milk before incubation with primary and secondary antibodies and development using chemiluminescense (Millipore). Protein levels were corrected for Coomassie staining of total protein gels ran or protein stain on membrane (Memcode, Pierce). Densitometry was performed using Image Fiji/ImageJ.

Reference

Cheung, C., Bernardo, A.S., Trotter, M.W.B., Pedersen, R.A., and Sinha, S. (2012). Generation of human vascular smooth muscle subtypes provides insight into embryological origin-dependent disease susceptibility. Nat Biotechnol *30*, 165–173.

Hollmann, E.K., Bailey, A.K., Potharazu, A. v, Neely, M.D., Bowman, A.B., and Lippmann, E.S. (2017). Accelerated differentiation of human induced pluripotent stem cells to blood-brain barrier endothelial cells. Fluids Barriers CNS *14*, 9.

Lee, C.A.A., Seo, H.S., Armien, A.G., Bates, F.S., Tolar, J., and Azarin, S.M. (2018). Modeling and rescue of defective blood-brain barrier function of induced brain microvascular endothelial cells from childhood cerebral adrenoleukodystrophy patients. Fluids Barriers CNS *15,* 9.

Murray, L.S., Lu, Y., Taggart, A., Van Regemorter, N., Vilain, C., Abramowicz, M., Kadler, K.E., and Van Agtmael, T. (2014). Chemical chaperone treatment reduces intracellular accumulation of mutant collagen IV and ameliorates the cellular phenotype of a COL4A2 mutation that causes haemorrhagic stroke. Hum Mol Genet *23*, 283–292.

Orlova, V. v., Drabsch, Y., Freund, C., Petrus-Reurer, S., van den Hil, F.E., Muenthaisong, S., ten Dijke, P., and Mummery, C.L. (2014a). Functionality of endothelial cells and pericytes from human pluripotent stem cells demonstrated in cultured vascular plexus and zebrafish xenografts. Arterioscler Thromb Vasc Biol *34*, 177–186.

Serrano, F., Bernard, W.G., Granata, A., Iyer, D., Steventon, B., Kim, M., Vallier, L., Gambardella, L., and Sinha, S. (2019). A Novel Human Pluripotent Stem Cell-Derived Neural Crest Model of Treacher Collins Syndrome Shows Defects in Cell Death and Migration. Stem Cells Dev *28*.

Shah, S., Kumar, Y., McLean, B., Churchill, A., Stoodley, N., Rankin, J., Rizzu, P., van der Knaap, M., and Jardine, P. (2010). A dominantly inherited mutation in collagen IV A1 (COL4A1) causing childhood onset stroke without porencephaly. Eur J Paediatr Neurol *14*, 182–187. https://doi.org/10.1016/J.EJPN.2009.04.010.
